# Genomic Analysis of Spontaneous Abortion in Holstein Heifers and Primiparous Cows

**DOI:** 10.3390/genes10120954

**Published:** 2019-11-21

**Authors:** Kayleen F. Oliver, Alexandria M. Wahl, Mataya Dick, Jewel A. Toenges, Jennifer N. Kiser, Justine M. Galliou, Joao G. N. Moraes, Gregory W. Burns, Joseph Dalton, Thomas E. Spencer, Holly L. Neibergs

**Affiliations:** 1 Department of Animal Sciences and Center for Reproductive Biology, Washington State University, Pullman, WA 646310, USA; kayleen.oliver@wsu.edu (K.F.O.); alexandria.wahl@wsu.edu (A.M.W.); mataya.dick@wsu.edu (M.D.); jewel.toenges@wsu.edu (J.A.T.); jennifer.kiser@wsu.edu (J.N.K.); justine.galliou@wsu.edu (J.M.G.); 2 Animal Sciences Research Center, Division of Animal Sciences, University of Missouri, Columbia, MO S158A, USAgbvd6@mail.missouri.edu (G.W.B.); spencerte@missouri.edu (T.E.S.); 3 Department of Animal and Veterinary Sciences, University of Idaho, Caldwell, ID 1904 E, USA; jdalton@uidaho.edu

**Keywords:** cattle, spontaneous abortion, dairy, loci

## Abstract

Background: The objectives of this study were to identify loci, positional candidate genes, gene-sets, and pathways associated with spontaneous abortion (SA) in cattle and compare these results with previous human SA studies to determine if cattle are a good SA model for humans. Pregnancy was determined at gestation day 35 for Holstein heifers and cows. Genotypes from 43,984 SNPs of 499 pregnant heifers and 498 pregnant cows that calved at full term (FT) were compared to 62 heifers and 28 cows experiencing SA. A genome-wide association analysis, gene-set enrichment analysis–single nucleotide polymorphism, and ingenuity pathway analysis were used to identify regions, pathways, and master regulators associated with SA in heifers, cows, and a combined population. Results: Twenty-three loci and 21 positional candidate genes were associated (*p* < 1 × 10^−5^) with SA and one of these (KIR3DS1) has been associated with SA in humans. Eight gene-sets (NES > 3.0) were enriched in SA and one was previously reported as enriched in human SA. Four master regulators (*p* < 0.01) were associated with SA within two populations. Conclusions: One locus associated with SA was validated and 39 positional candidate and leading-edge genes and 2 gene-sets were enriched in SA in cattle and in humans.

## 1. Introduction

Pregnancy loss, primarily spontaneous abortion (SA), is not well understood. For women, 10–20% of all recognized pregnancies will result in an SA [1]. Especially devastating are the 1–2% of women that experience recurrent SA (three or more pregnancies lost prior to 20 weeks) [1]. Spontaneous abortions have been associated with factors such as chromosomal abnormalities, uterine anatomical defects, and immunological factors [1]. The limited availability of human samples has made it difficult to study the etiology of SA and reduce SA in humans. Mice are commonly used to study human gestation research due to low costs, a short 20 days gestation, and genetic engineering [2]. However, there are limitations in using mice as a model for human pregnancy. Mice are a litter bearing species, and their significantly shortened gestation period makes it difficult to measure individual fetal progress and placental development needed for human studies [2]. These disparities encourage the use of models other than murine species to study the etiology and genetic factors that contribute to SA. Cattle and humans have a similar gestation lengths, and usually bear a single offspring per gestation, suggesting that cattle may be a good model for pregnancy loss in humans even though cattle have morphological differences in anatomy and placental attachment to humans [3].

In the dairy industry, subfertility has been an issue of major economic impact for decades [4]. Reduced fertility limits productivity and profitability due to increased veterinary costs, additional inseminations, and elevated culling rates [5,6]. The dairy industry suffers economic losses reaching over one billion dollars annually as a result of this poor reproductive performance [7]. Although approximately 80% of pregnancy losses occur before day 35 post-insemination [8,9,10,11,12], an additional 7–10% of females will experience fetal loss after day 35. Fetal loss costs the industry between $437 and $484 million annually due to the cost of maintaining the animal during pregnancy, the loss of the calf and subsequent lactation, and the costs associated with either rebreeding the animal or replacement [7]. In order to reduce the economic burden of pregnancy loss, producers have been selecting for fertility traits such as conception rate (the percentage of inseminated females that become pregnant at each service). Consequently, in Holstein cattle, conception rate has improved approximately 6 percentage points in heifers and 11 percentage points in cows since its inclusion in selection indexes in 2010 [13,14]. Although more recent studies have focused on the more prevalent early embryonic loss [15,16,17,18], additional research investigating fetal loss is needed, as very little is known about the genomic structure and physiological mechanisms responsible. In order to improve upon that knowledge, it is necessary to understand which genomic regions, genes, gene-sets, and pathways are associated with SA. Therefore, the objectives of this study were to identify 1) loci associated with SA in US Holstein cattle using genome-wide association analysis (GWAA), 2) gene-sets and leading-edge genes enriched in SA through gene-set enrichment analysis–single nucleotide polymorphism (GSEA–SNP), and 3) pathways and regulators responsible for SA through an ingenuity pathway analysis (IPA). These results will provide a foundation for the identification of causal mutations associated with SA in dairy cattle.

## 2. Materials and Methods

### 2.1. Study Population and Phenotype

This study was performed with the approval of the Institutional Animal Care and Use Committee at Washington State University (4295). In this study, two populations were assessed: 3359 Holstein heifers from a commercial heifer raising facility in southern Idaho and 2015 primiparous Holstein cows from six central Washington dairies. Heifers were eligible for artificial insemination (AI) after reaching approximately 11–13 months of age, a height of 129 cm at the withers, and a weight of 390 kg. Heifers received AI at observed estrus using semen representing 51 different Holstein sires. Cows received AI at observed estrus or timed AI using 433 Holstein and 2 Angus sires. The conception rate between AI sires was not significant (*p* = 0.07) and sexed semen was not used. Heifers were bred by one of three AI technicians, and the conception rate did not differ between technicians (*p* > 0.05). For cows, AI was performed (depending on individual dairy practices) by one of 34 technicians, with no significant difference (*p* > 0.05) demonstrated in conception rates between technicians. Cattle were followed after AI to parturition to determine if any SA occurred.

Pregnancy was determined via transrectal palpation of uterine contents 35 days after AI. DairyComp305 (Valley Agricultural Software, Tulare, CA) health records were used to determine if cattle aborted a fetus, and to remove animals with cofounding ailments including metritis, fever, lameness, mastitis, metabolic issues, pink eye, and respiratory disease. Only cattle that were pregnant after the first AI were considered for this study, leaving 561 heifers and 526 cows available for genotyping. Cattle were classified as those that calved at full term (FT) or those that spontaneously aborted (SA) prior to their pregnancy reaching full term. The heifer population consisted of 499 FT and 62 SA heifers. The cow population consisted of 498 FT cows and 28 SA cows. The combined heifer and cow population consisted of 997 FT cattle and 90 SA cattle. 

### 2.2. DNA Extraction and Genotyping

Approximately 16 ml of whole blood was collected in ethylenediaminetetraacetic acid (EDTA) tubes from cattle via venipuncture of the tail vein. DNA was extracted from white blood cell pellets using the Puregene DNA extraction kit following manufacturer’s instructions (Gentra, Minneapolis, MN, USA). Following extraction, DNA was quantified using the Nanodrop 1000 spectrophotometer (Thermo Fisher Scientific, Wilmington, DE, USA) and genotyped at Neogen GeneSeek Laboratories (Lincoln, NE, USA) using the Illumina BovineHD BeadChip (San Diego, CA, USA) for FT cattle and using the GeneSeek Bovine GGP50K BeadChip (Lincoln, NE, USA) for SA cattle. The Illumina BovineHD BeadChip contains 778,962 single nucleotide polymorphism (SNPs) with an average distance of 3.34 kb between SNPs [19] and the GeneSeek Bovine GGP50K BeadChip contains 47,843 SNPs with an average distance of 59 kb between SNPs [20]. The GWAA was conducted using the 43,984 SNPs shared between the Illumina BovineHD BeadChip and the GeneSeek GGP50K BeadChip.

### 2.3. Quality Control

#### 2.3.1. Heifers

Prior to the GWAA, quality control filtering for SNPs and cattle was performed. The SA cattle represented a much smaller number than the FT cattle, which necessitated that SA cattle underwent quality control separately from the FT cattle. Only SNPs that were shared in SA and FT groups (after quality control) were used for analyses. 

Quality control was first completed for SNPs, where SNPs with >10% of genotypes missing were removed. For SA heifers, 715 SNPs were removed and for FT heifers, 4289 SNPs were removed. For SNPs with minor allele frequencies (MAF) <1%, 1444 SNPs were removed for the SA heifers and 1198 SNPs were removed for FT heifers. Hardy–Weinberg equilibrium testing (*p* < 1 × 10^−50^) removed no SNPs for the SA heifers, but an additional four SNPs were removed in the FT heifers. When the SA and FT groups were combined, 37,954 SNPs remained for the analyses. 

Quality control for heifers consisted of removing 29 FT heifers and 1 SA heifer due to a poor genotyping call rate, as >10% of their genotypes were missing. Two SA heifers were identified as duplicates using an identity by decent matrix and were removed from the analysis. A sex check to determine concordance with genotypic and phenotypic sex designation resulted in no heifers being removed. After quality control, 59 SA and 470 FT heifers remained available for analyses. 

#### 2.3.2. Cows

The thresholds for quality control filtering of SNPs and cattle were the same for the cows as for the heifers. As with the heifers, SA and FT cows were quality control filtered separately prior to being combined together for analyses due to the differences in sample sizes of the two groups. For SA cows, 1696 SNPs were removed for a genotyping call rate <90%, and 766 SNPs were removed for the FT cows. The number of SNPs removed due to an MAF <1% was 1524 for the SA cows and 1344 for the FT cows. Hardy–Weinberg equilibrium testing (*p* < 1 ×10^−50^) removed no SNPs for the SA cows, but removed 10 SNPs in the FT cows. When the SA and FT groups were combined, 40,193 SNPs remained for the analyses.

Quality control for heifers consisted of removing 9 FT cows due to a poor genotyping call rate, as >10% of their genotypes were missing, but no SA cows were removed due to a poor genotyping call rate. A sex check to determine concordance with genotypic and phenotypic sex designation resulted in no cows being removed. After quality control, 28 SA and 489 FT cows remained available for analyses. 

#### 2.3.3. Heifers and Cows Combined

The SA heifer and SA cow groups were combined and underwent quality control separately from the combined FT heifer and FT cow groups. Only SNPs passing quality control and shared in the SA and FT combined groups were used for the analyses. SNPs with a poor genotyping call rate (<90%) resulted in an additional 1763 SNPs being removed from the SA combined group, and 2452 SNPs being removed from the FT combined group. The evaluation of SNPs with an MAF <1% resulted in the removal of 1311 SNPs from the SA combined group and 1243 SNPs from the FT combined group. Hardy–Weinberg equilibrium testing (*p* < 1 × 10^−100^) removed no SNPs in the SA combined group, but removed an additional seven SNPs in the FT combined group. When the SA and FT groups were combined, 39,019 SNPs remained for the analyses. 

Quality control for genotyping call rate (<90%) in the combined cattle groups resulted in the removal of 1 SA (1 heifer) and 38 FT (29 heifers and 9 cows) cattle. Two SA heifers were identified as duplicates using an identity by decent matrix and were removed from the analysis. A sex check to determine concordance with genotypic and phenotypic sex designation resulted in no heifers or cows being removed. After quality control, 87 SA and 959 FT females remained available for analyses in the combined heifer and cow population. 

### 2.4. Genome-Wide Association Analysis

Three GWAA were performed to identify loci associated with SA in heifers, cows, and the combined population. The GWAA were performed using an efficient mixed-model associated eXpedited (EMMAX) method with a genomic relationship matrix [21] and an additive model in the SNP and Variation Suite (SVS) software version 9.1 (Golden Helix, Bozeman, MT) [22]. The additive model identifies associations that depend on values of the alleles having an additive effect, where having two minor alleles (aa) is twice as likely to impact the phenotype (SA) compared to having no minor alleles (AA), and half as likely to have an impact as when one minor (Aa) is present. The general mixed model was described as y=Xβ+Zu+ϵ, where y describes the *n* × 1 vector of observed phenotypes, X was an *n* × *f* matrix of fixed effects (*f*), β was an *f* × 1 vector containing the fixed effect coefficients, Z was an *n* × *t* matrix relating the random effects (*t*) to the phenotype, and u was the random effect of the mixed model [23]. This model assumes residuals to be independent with an identical distribution where $ar\left(u\right)=\sigma_{g}{}^{2}K$ and $\left(\epsilon \right)=\sigma_{e}{}^{2}I$, and $Var\left(y\right)=\sigma_{g}{}^{2}ZKZ^{\prime }+\sigma_{e}{}^{2}I$. This study used K as a matrix of pairwise genomic relationships and Z as the identity matrix, I [23]. 

The pseudo-heritability estimated by EMMAX can be over-inflated with limited sample sizes, thus heritability was estimated using a genomic best linear unbiased predictor (GBLUP) analysis [24] with the average information algorithm (AI–REML), which is a bivariate restrict maximum likelihood analysis [25,26]. The AI–REML GBLUP method is often used to calculate heritability at the expense of increased computational time. Additional documentation of SVS methods for EMMAX, pseudo-heritability, and GBLUP with AI–REML are available at 

Loci were considered associated with SA based on the Wellcome Trust Case Control Consortium recommendation where unadjusted *P*-values between 1 × 10^−5^ and 5 × 10^−7^ indicated a moderate association and unadjusted *P*-values less than 5 × 10^−7^ provided evidence for a strong association [27,28]. Positional candidate genes were identified within a 34 kb region (17 kb 5′ and 3′) of associated SNPs based on the average haplotype block size for Holstein cattle using the method previously described by Gabriel et al. (2002) [29] and based on a large Holstein population (*n*  =  2700), genotyped using the Illumina BovineHD BeadChip [30]. 

### 2.5. Gene-Set Enrichment Analysis–Single Nucleotide Polymorphism 

The GSEA–SNP analysis was performed with the GenGen software package [30], and SNPs were mapped to corresponding genes based on the UMD 3.1 assembly. Gene-set analyses were generated from five databases: Gene Ontology (GO) (*n*  =  3147), Reactome (R) (*n*  =  674), BioCarta (*n*  =  217Kyoto encyclopedia of gene and genomes biological pathways (KEGG) (*n*  =  186), and Protein Analysis Through Evolutionary Relationships (PANTHER) (*n*  =  165). All five of the databases are a collection of biological pathways (gene-sets) that focus on human and other organisms. Gene-sets are comprised of genes that have been identified by the literature to interact with one another. These gene-sets identify molecular interactions and relationships for genes including, but not limited to, those involved in metabolism, genetic information processing, environmental information processing, cellular processes, organismal systems, human diseases, and drug development. These gene-sets serve as a model for the relationships of genes present in cattle where less information is known than in some model organisms. Each gene in a gene-set was represented by the single most significant SNP located either within a gene or near the gene (within a haplotype block that the gene was present in). A single SNP was assigned to represent a gene as it removed the risk of weight inflation, which may arise when multiple SNPs are used to represent a single gene. The most significant SNP for each gene was used as the proxy for 19,723 genes. 

All SNPs were ranked by their significance of association with SA. Running sum statistics and enrichment scores (ES) were calculated for each gene-set. Enrichment scores increase when a ranked gene associated with SA is encountered in the gene-set, and scores decrease when the gene within the gene-set is not associated with SA. The ES statistics were calculated much like the weighted Kolmogorov–Smirnov statistic [31]. Leading-edge genes, the genes associated with SA within the gene-set that positively contribute to the peak ES, were also identified. The permuted *P*-value was calculated using GenABEL in R with 10,000 phenotype-based permutations for each gene-set [32]. The ES were normalized (normalized enrichment scores, NES) to account for variances in the number of genes within each gene-set. The thresholds for significance for NES range from 1.5 to 3.0 [33,34] with previous fertility studies using an NES >3.0 [16]. For this study, a gene-set was significant if the NES was >3.0. 

### 2.6. Network Analysis

Ingenuity pathways analysis (IPA; Ingenuity Systems) was used to identify relationships between the positional candidate and leading-edge genes from the GWAA and GSEA–SNP, respectively. Gene network analyses included identifying canonical pathways and upstream and master regulators, with positional candidate genes from the GWAA and leading-edge genes from the GSEA–SNP. Canonical pathways are gene networks that include genes associated with SA and that interact with genes in the pathway. Canonical pathways identify how altering the gene expression of one gene may impact many other genes that are downstream or upstream in the network. Upstream regulators are genes that control multiple genes through direct or indirect relationships, whereas master regulators are the molecules indirectly connected to genes within the dataset via upstream regulators [35]. For a full review of IPA methodology see Krämer and colleagues [35] Significance for the IPA was determined using a Benjamini–Hochberg corrected *p* < 0.01.

## 3. Results

### 3.1. Genome-Wide Association Analyses

In the heifer population, seven loci were strongly associated (*p* < 5 ×10^–7^) and four loci were moderately associated (1 × 10^−5^ > *p* < 5 × 10^−7^) with SA (Table 1). Of these associated loci, seven loci contained 11 positional candidate genes. The estimated heritability for SA in heifers was 0.75 (±0.01). 

For cows, four loci were strongly (*p* < 5 × 10^−7^) and three loci were moderately associated (1 × 10^−5^ > *p* < 5 × 10^−7^) with SA (Table 1). Four of the seven loci associated with SA in cows contained nine positional candidate genes. Two loci on BTA11 (*rs41822060*) and BTAX (*rs137198342*) were associated in the heifer and cow populations. The estimated heritability for SA in cows was 0.09 (±0.08). This estimate varies from the heritability report for SA in heifers but is similar to that reported in humans 0.015 (±0.4) [36]. The number of SA cows was lower than the number of heifers that had an SA. The smaller population results in an increase in cow variability, lowering the heritability estimate. A second confounding factor is the preselection of the primiparous cow population. For primiparous cows to be bred, only animals that conceived and calved at full term as a heifer were bred as a primiparous cows. This will also reduce the heritability estimate. 

The combined population had eight loci strongly associated (*p* < 5 × 10^−7^) and seven loci moderately associated (1 × 10^−5^ > *p* < 5 × 10^−7^) with SA. Eight of the 15 loci contained 12 positional candidate genes (Table 1). The estimated heritability for SA in the combined population was intermediate between the two individual populations at 0.31 (±0.08). Seven loci (BTA2, BTA10, BTA24, BTA27, and BTA28) were associated (*p* < 1 × 10^−5^) with SA in the heifer and combined populations (Table 1). While a single locus on BTA14 (*rs41822060*) was associated (*p* < 1 × 10^−5^) with SA in the cow and combined populations, and two loci, on BTA11 and BTAX, were associated (*p* < 1 × 10^−5^) with SA in all three populations. 

### 3.2. Gene-Set Enrichment Analyses–Single Nucleotide Polymorphisms

The gene-set chromosome organization was enriched (NES = 3.13, GO: 0051276) for SA in the heifer population (Table 2). This gene-set included 54 genes with 17 leading-edge genes. For the cow population, two gene-sets, lipid catabolic process (NES = 3.37, GO: 0016042) and glycine serine and threonine metabolism (NES = 3.01, KEGG: hsa00260), were enriched in the SA cohort (Table 2). The lipid catabolic process gene-set included 46 genes and identified 14 leading-edge genes associated with SA, whereas the glycine serine and threonine metabolism gene-set contained 21 genes and identified five leading-edge genes associated with SA. Five gene-sets were enriched in the combined population and 48 unique leading-edge genes were identified as associated with SA (Table 2). Two leading-edge genes, *PRKCB* and *CAMK2G,* were shared between the heifer and combined populations. One of these, *CAMK2G,* was also a positional candidate gene for both the heifer and combined populations in the GWAA. Additionally, the leading-edge gene *PLCB1* was associated with SA in the cow and combined populations.

### 3.3. Ingenuity Pathway Analysis

For the IPA analyses, 23 positional candidate genes and 83 leading-edge genes were used. In the heifers, no canonical pathways or upstream regulators were associated (*p* < 0.01) with SA. However, 138 master regulators (Table S1) were identified (*p* < 0.01). In the cows, 10 canonical pathways (*p* < 0.01, Table S2) were associated with SA and 102 master regulators (*p* < 0.01; Table S3) were identified as regulating genes associated with SA. No upstream regulators that regulated 266 SA positional candidate or leading-edge genes were found (*p* < 0.01). In the combined population, 223 canonical pathways (Table S4) were associated with SA (*p* < 0.01). In addition, 86 upstream regulators (Table S5) and 224 master regulators (Table S6) that regulated SA positional candidate or leading-edge genes were identified. 

Canonical pathways, upstream regulators, and master regulators were evaluated to determine if they were shared across the cattle populations. Six canonical pathways (Table 3) were shared in the cows and combined populations and four master regulators (NMB, FGF3, CAB39, PLCB4) (Table 4) were shared between two of the three cattle populations. 

## 4. Discussion

### 4.1. Positional Candidate Genes

The GWAA identified 23 loci containing 21 positional candidate genes that were associated with SA in Holstein cattle (Table 1). The 21 positional candidate genes are involved in a variety of physiological functions spanning from cell signaling cascades [45] to regulating lymphatic development in mammals [46]. The loci on BTA11 (*rs41822060*) and BTAX (*rs137198342*) contained four positional candidate genes that were associated with SA in all three populations suggesting that these loci are important in SA regardless of parity and will be further discussed below. 

Three of the four positional candidate genes on BTA11 (*GAB3*, *KIR3DS1*, *LOC786275*) had functions involved in the immune response. The genes *KIR3DS1* and *LOC786275* produce killer cell immunoglobulin cell receptors. The killer cell immunoglobulin-like receptors (KIR) are a family of inhibitory or activating cell surface receptors expressed on natural killer cells and some T lymphocyte subpopulations [47]. Natural killer cells are granular cells that account for 10–15% of lymphocytes in the blood and serve a vital role in immune regulations and defense [48,49]. Prior to implantation, natural uterine killer cells accumulate and represent the majority of decidua lymphocytes in the endometrium during early pregnancy in humans [48,50]. Multiple studies have identified associations with recurrent SA and killer cell immunoglobulin-like receptor proteins in humans [51,52,53,54,55,56,57,58,59,60,61,62], although some studies have refuted these associations [63,64]. While associations have been identified with *KIR3DS1* and SA in cattle and humans, functional studies in cattle are needed to confirm the role of *KIR3DS1* in pregnancy loss. 

The positional candidate gene *GAB3* is a member of the GRB2 binder family that includes scaffolding/docking proteins involved in signaling pathways mediated by growth factors, cytokines, and antigen receptors [65], and has roles in immune response and tumor cell proliferation [66,67]. These proteins are located within the cytosol until tyrosine phosphorylation occurs, when they migrate to the plasma membrane and function as adaptor proteins [68]. The GRB2 binder family is also highly expressed in T cells, B cells, macrophages, and mast cells [66,68,69]. Murine studies have shown that the loss of Gab3 in *Gab3^–/–^* mice results in normal embryonic, hematopoiesis, and macrophage development into adulthood [65]. The exact role of *GAB3* in cattle is uncertain, but it may function in maternal recognition and maintenance of pregnancy [68]. 

The fourth positional candidate gene, *AFF3*, is a lymphoid-specific gene expressed in B cells and is commonly involved in oncogenesis [70]. B cells are a major component of the immune system, but limited research has been done to investigate their role in pregnancy. Studies that have investigated B cell interaction with SA have suggested that in humans, women experiencing SA have a lower proportion of regulatory B cells and asymmetric antibodies when compared to women undergoing normal pregnancies [71,72]. The reduced levels of antibodies needed to block immune responses may lead to the inability of the fetus to protect itself from a maternal attack, thus terminating pregnancy. In cattle, there has been evidence that *AFF3* has a functional role in fertility as it was differentially expressed in the endometrium of high fertile pregnant and non-pregnant heifers [17]. While these studies were aimed towards identifying genetic factors influencing early pregnancy success, this overlap with SA suggests *AFF3* may also have biological functions that impact fetal loss. 

### 4.2. Gene-Sets Enriched for Spontaneous Abortions

The GSEA–SNP analysis identified four KEGG, two GO, and two R gene-sets (Table 2) that were enriched for SA in Holstein cattle. Two gene-sets shared functions associated with the mitotic cell cycle (R: REACT_21391, R: REACT_15296) and chromosomal organization (GO: 0051276), and three gene-sets shared functions associated with neurological functions (KEGG: hsa04720, KEGG: hsa04912, KEGG: hsa05214). The two remaining gene-sets were involved with lipid (GO: 0016042) and protein (KEGG: hsa00260) metabolism. The functions of all gene-sets enriched for this study are present in Table 2. Identified leading-edge genes were compared to external studies investigating cattle fertility, and 36 leading-edge genes overlapped with one or more studies (Table 5). Since KEGG: hsa00260 was previously associated with SA, this paper will focus on metabolism gene-sets with respect to the leading-edge genes shared across two or more populations.

Metabolism has an integral impact on pregnancy, primarily through lipid catabolic processes (GO: 0016042) [75,76] and amino acids such as serine, glycine, and threonine (KEGG: hsa00260) [39]. Fatty acids, which include long-chain polyunsaturated fats for nervous system components [77], are important nutrients for embryonic development. Placental metabolism of eicosanoids is important for nutrient transfer from the maternal side to the fetus [75,78]. Li and coworkers (2018) investigated the association of fatty acids and lipids in the placenta with SA in early pregnancy loss (4–10 weeks) in humans, and concluded that as eicosanoid synthesis and essential fatty acid levels were altered, there was an increased risk for early spontaneous pregnancy loss. In cattle, six leading-edge genes (*PLCB1*, *ENPP2*, *PLA2G2A*, *PAFAH1B1*, *PAFAH2*, and *SMPDL3A*) in the lipid catabolic process gene-set have previously been associated with fertility (Table 5). The *ENPP2* gene encodes ENPP2 that can act as a phosphodiesterase and phospholipase, which is involved in catalyzing lysophosphatidic acid (LPA) in extracellular fluids [79]. The production of LPA via *ENNP2* stimulates motility in various cell types that induce cellular responses such as platelet aggregation and smooth muscle contraction [80], which are important uterine responses during pregnancy. Additionally, one unique leading-edge gene in this gene-set, *LIPE*, is involved in adipose tissue mobilization during pregnancy in Holstein cattle [73], suggesting that this gene-set may play a crucial role in pregnancy maintenance.

In humans, the glycine, serine, and threonine metabolic pathways were identified as key biological pathways contributing to reoccurring SA [38], as altered amino acid profiles may contribute to restricted fetal growth through their roles in cellular metabolism and proliferation [39]. In cattle, research regarding lipid and amino acid metabolism in SA is lacking; however, two leading-edge genes (*SARDH* and *DAO*) were both differentially expressed in the endometrium of highly fertile pregnant heifers when compared to highly fertile open heifers, just as *SARDH* was differentially expressed in the endometrium of subfertile heifers by pregnancy [17].

Two leading-edge genes (*PLCB1* and *PRKCB*) were shared across two populations. The leading-edge gene *PLCB1* was enriched in the cow and combined populations, while the second leading-edge gene, *PRKCB,* was enriched in the heifer and combined populations. The leading-edge gene, *PLCB1*, encodes phospholipase C-beta proteins [42]. Phospholipase C-beta catalyzes inositol 1,4,5-trisphophaate (IP3) and diacylglycerol (DAG) from phosphatidylinositol 4,5-bisphosphate (IP2), which are key steps for intracellular transduction of various extracellular signals [42] in the GnRH signaling pathway (KEGG: hsa04912). The activation of the GnRH signaling pathway occurs as the G–protein–protein coupled GnRH receptor binds GnRH and activates phospholipase C [42]. The PLCB1 protein is a downstream effector of the G–protein–protein coupled GnRH receptor [81]. The G–protein–protein coupled receptors (GPCRs) have central roles in mediating fertility as hormones such as GnRH, FSH, LH, and oxytocin all signal through GPCRs [81]. Thus, GPCR disruptions can have adverse effects on fertility through the disruption of the hypothalamic–pituitary–gonadal axis. Filis and colleagues (2013) investigated implantation failure of Plcb1 knockout mice and concluded that disruptions in *Plcb1* mice negatively impact uterine steroid responses, luminal epithelial differentiation, and endocannabinoid metabolism. Additionally, *PLCB1* was previously identified as a positional candidate gene for primary ovarian failure and ovarian dysfunction in humans [82], suggesting that *PLCB1* may have a crucial role in early pregnancy success. 

The *PRKCB* gene is a member of the protein kinase C family of serine/threonine kinases that have functions in signal transduction pathways involving hormone release, mitogenesis, tumor progression [83], and regulation of uterine smooth muscle contractions [84,85,86,87]. Abnormalities in uterine contractility can lead to various clinical problems including pre-term labor in humans [87]. Parco and coworkers (2007) investigated the role of the protein kinase C family and concluded that the isoform of the protein kinase C-pathway (aPRKC) is involved with uterine contractility during the end of gestation, and linked aPRKC to inflammatory signaling within the myometrium [88]. Other studies investigating cattle fertility and uterine receptivity had identified a role for *PRKCB* (Table 5). However, further studies are needed to better understand the controversial role of PRKC isoforms in uterine contractility, as previous findings have had contradictory conclusions [89,90].

### 4.3. Master Regulators Shared in Two or More Populations

Master regulators are molecules that indirectly control gene expression of positional candidate or leading-edge genes through upstream regulators. For this study, only four master regulators, FGF3, NMB, CAB39, and PLCB4, were shared for all three populations. 

The FGF3 protein was identified as a master regulator associated with SA in the heifer and combined populations. In the heifer population, FGF3 is predicted to regulate five positional candidate genes and 10 leading-edge genes associated with SA. For the combined population, three positional candidate and 29 leading-edge genes were predicted to be affected (Table 4). The FGF3 master regulator is a member of the fibroblast growth factor family and plays a critical role in embryonic development by regulating cell proliferation, differentiation, and migration [91,92]. While no direct link to SA has been identified, an interaction between the *FGF3* regulatory promoter and *SOX6* causes repression of transcription of the *FGF3* gene [93], which may play a role in improper embryonic development as cellular regulation controlled through *FGF3* would be impaired. 

The NMB peptide is a master regulator associated with SA in the heifer and cow populations. The *NMB* gene is a member of the bombesin-like peptide family and is involved in smooth muscle contraction [94,95], stress responses [96], and cellular growth and differentiation [97,98]. In the heifer population, NMB is predicted to regulate three positional candidate genes and 10 leading-edge genes associated with SA in heifers (Table 3). For the cow population, NMB is predicted to regulate three positional candidate genes and 10 leading-edge genes associated with SA (Table 3). While research regarding its impact on fetal loss is limited, Zhang and colleagues (2012) investigated Nmb and its receptor’s (Nmbr) roles in pre-term pregnancies in mice. The authors suggested that the interaction between *Nmb/Nmbr* may influence myometrial cells via the Rela/p65 pathway [99]. The Rela/p65 pathway is an important upstream regulator of the labor-induced processes involving uterine smooth muscle through regulation of phospholipase C/protein kinase C, myosin light-chain phosphorylation, and free intracellular calcium [99,100]. Mutations or dysregulation of this NMB may induce an SA or pre-term birth due to its involvement in regulating genes involved in calcium cascades (*NCLAD*, *CAMK2G, PLCB1*, *PRKCB*) and its previous association with uterine smooth muscle contractions [97,99]. 

The final two master regulators, CAB39 and PLCB4, were shared between the cow and combined populations. In the cow population, CAB39 is predicted to regulate two positional candidate and 11 leading-edge genes associated with SA (Table 4). In the combined populations, two positional candidate and 28 leading-edge genes were predicted to be regulated. The master regulator CAB39, previously identified as MO25, regulates protein kinases, and has been identified to be involved in cellular invasion and in both hepatic [101] and pancreatic cancers [102]. Further investigation of the role of CAB39 is needed to understand its impact on pregnancy loss. 

The master regulator, PLCB4, is a member of the phospholipase C-beta family, and is related to the same family as the leading-edge, *PLCB1*. As a master regulator, PLCB4 is predicted to regulate one leading-edge gene (*PLCB1*) and is involved in multiple physiological processes and pathways, most commonly involved in calcium signaling [103,104,105], which is crucial for proper uterine quiescence and smooth muscle contractions during gestation. While research regarding PLCB4 in SA is lacking, it has been suggested to be involved in the embryonic development of internal and external facial structures [106], with interruptions potentially leading to fetal death if development is compromised.

## 5. Conclusions

Loci, positional candidate genes, gene-sets, leading-edge genes, pathways, and upstream and master regulators associated with SA in Holstein heifers and primiparous cattle were identified in this study. Many of the positional candidate and leading-edge genes and gene-sets that were identified in cattle are also important in SA in humans. Therefore, this research sets a foundation for further investigation into the mechanisms associated with SA in cattle and may also be beneficial in understanding similar mechanisms in human SA. Enhancing our understanding of the underlying factors contributing to SA in cattle will provide an opportunity to use bovine models to identify alternative approaches and functional studies that can be used to reduce the onset of spontaneous abortions in women. 

In cattle, fetal loss is a financial burden for producers. To the authors’ knowledge, this is the first study investigating the genomic foundations of SA in cattle. By identifying genomic regions and genes contributing to SA, SNPs associated with SA can be used for genomic selection. The validation of one locus, two gene-sets, and 39 positional candidate or leading-edge genes from human studies and cattle populations from different parities, dairies, and human studies supports these findings as robust predictors of SA in cattle. However, due to the limited sample size of this study, further validation of these results is warranted, and correlations with production traits should be investigated. Future studies should focus on the identification of causal mutations leading to SA to increase the accuracy of genomic selection and identify potential therapies to reduce pregnancy loss. 

## Figures and Tables

**Table 1:** Loci associated with spontaneous abortions in Holstein cattle.

Chromosome (Location in Mb) ^1^	SNP ID ^2^	Population ^3^	*p-*Value ^4^	Positional Candidate Gene(s) ^5^
1 (9–10)	*rs110416429*	C	2.32 × 10^−7^	-
2 (88–89)	*rs41640492*	H H + C	2.70 × 10^−8^ 1.11 × 10^−13^	-
7 (15–16)	*rs137810199*	H + C	1.23 × 10^−6^	*LOC100300764, LOC100300806*
7 (83–84)	*rs109588489*	H	4.55 × 10^−7^	***ACOT12**, LOC104968970*
7 (97–98)	*rs110673622*	H + C	5.58 × 10^−7^	-
8 (31–32)	*rs41659609*	H + C	1.85 × 10^−6^	*LOC104969317, LOC782470*
10 (42–43)	*rs41618585*	H H + C	1.15 × 10^−14^ 4.21 × 10^−20^	-
10 (56–57)	*rs43014621*	H + C	7.35 × 10^−7^	*WDR72*
10 (98–99)	*rs42814099*	C	6.22 × 10^−6^	-
11 (5–6)	*rs41822060*	H C H + C	1.39 × 10^−7^ 2.18 × 10^−7^ 1.15 × 10^−14^	***AFF3***
14 (64–65)	*rs43430961*	C H + C	1.26 × 10^−9^ 2.83 × 10^−9^	***NCALD***
16 (70–71)	*rs109993299*	H	9.68 × 10^−6^	***PTPN14***
18 (4–5)	*rs43002722*	H	8.55 × 10^−8^	-
18 (21–22)	*rs41872094*	H	8.61 × 10^−8^	***AKTIP**, RBL2*
20 (27–28)	*rs109855946*	H + C	6.31 × 10^−11^	-
21 (53–54)	*rs133762978*	H + C	5.46 × 10^−6^	-
24 (36–37)	*rs42941005*	H H + C	1.18 × 10^−6^ 6.03 × 10^−8^	-
25 (39–40)	*rs133562920*	C	8.76 × 10^−6^	*ZNF12, LOC100140207, LOC100847388, LOC618542*
26 (9–10)	*rs133222015*	C	7.05 × 10^−6^	-
27 (108–109)	*rs133726820*	H H + C	5.29 × 10^−6^ 7.15 × 10^−6^	***LOC104970060***
28 (29–30)	*rs109459492*	H H + C	8.63 × 10^−7^ 2.45 × 10^−9^	***CAMK2G***
X (39–40)	*rs137198342*	H C H + C	2.22 × 10^−8^ 1.04 × 10^−14^ 1.46 × 10^−21^	*GAB3, KIR3DS1, LOC786275*
X (134–135)	*rs137331944*	H + C	2.38 × 10^−6^	-

^1^ Chromosome location of the associated locus followed by the location of the SNP on the chromosome in megabases (Mb), as measured by numbered nucleotides in the UMD3.1 reference genome assembly. ^2^ Each SNP was identified by the *rs* number which is a reference number assigned to markers submitted to the National Center for Biotechnology Information SNP database. The number of SNP associated with each quantitative trait loci (QTL) is listed in parentheses. ^3^ Association identified in heifers (H), cows (C), and a combined heifer and cow (H + C) population. ^4^ Significance (*p*-value) for the most significant SNP associated with spontaneous abortion. ^5^ Positional candidate genes were defined as genes located within 17 kb on either side of the associated SNP(s). Bolded gene names represent genes where SNP is located within the gene.

**Table 2:** Enriched gene-sets and leading-edge genes associated with spontaneous abortion in Holstein heifers and cows.

Gene-Set name (ID) ^1^	# Genes ^2^ (# LEG) ^3^	NES ^4^	Leading-Edge Genes (LEGs)	Gene-Set Function
**Holstein Heifers**				
Chromosome organization (GO: 0051276)	54 (17)	3.13	*BRE, TEX14, EYA2, BANP, RRS1, USP15, VPS72, ASCC3, MCM3, RNF20, SUDS3, PRKCB, OBFC1, POLE3, AURKA, CENPH, HMG20B*	The assembly or disassembly of chromosomes at the cellular level (Ashburner et al., 2000 [37])
**Holstein Cows**				
Lipid catabolic process (GO: 0016042)	46 (14)	3.37	*PLCB1, PAFAH1B3, IAH1, ENPP2, LIPE, NCEH1, PNLIPRP2, PLA2G2A, NEU3, PAFAH1B1, PAFAH2, DDHD1, PEX13, SMPDL3A*	Involved in the breakdown of lipids (Li et al., 2018 [38])
Glycine serine and threonine metabolism (KEGG: hsa00260)	21 (5)	3.01	*PSPH, CHDH, SARDH, DAO, CTH*	Involved in amino acid metabolism necessary for proper cellular regulation (Kalhan, 2016 [39])
**Combined Heifer and Cow Populations**				
Mitotic G2 G2/M phases (R: REACT_21391)	41 (14)	3.39	*PLK4, TUBGCP5, CEP164, PPP2R1A, CEP70, HSP90AA1, YWHAG, CEP76, NUMA1, CDK1, MNAT1, E2F3, CEP192, AKAP9*	Involved in the second growth phase/check-point (G2) and the transition from G2 to mitosis (M) during the cell cycle (Hung et al., 1996 [40])
Long term potentiation (KEGG: hsa04720)	43 (24)	3.31	*CAMK2G, GNAQ, ITPR1, PRKCB, RPS6KA3, PRKX, GRIN2A, RAF1, GRIA1, CAMK2A, CAMK2B, CAMK4, CALML5, PRKCA, RAP1A, PLCB1, RPS6KA2, GRM1, PPP3CC, PPP3CA, GRM5, ITPR2, GRIN2B, CAMK2D*	Involved in calcium signaling pathways in the hippocampus triggered by a postsynaptic rise in intracellular calcium concentrations (Neveu and Zucker, 1996 [41])
GnRH Signaling pathway (KEGG: hsa04912)	57 (16)	3.29	*CAMK2G, ADCY2, GNAQ, ITPR1, PRKCB, PLA2G5, PRKX, RAF1, ADCY9, CAMK2A, EGFR, PLA2G6, CAMK2B, CALML5, PRKCA, PLCB1*	Gonadotropin-releasing hormone (GnRH) secretion from the hypothalamus acts upon its receptor in the anterior pituitary to regulate the production and release of the gonadotropins, LH and FSH (Kanehisa and Goto, 2000 [42])
Recruitment of mitotic centrosome proteins and complexes (R: REACT_15296)	35 (12)	3.11	*PLK4, TUBGCP5, CEP164, PPP2R1A, CEP70, HSP90AA1, YWHAG, CEP76, NUMA1, CDK1, CEP192, AKAP9*	Produces proteins that serve as the microtubule organization center and is closely associated with the nucleus until the G2/M transition where the microtubule organization center matures into the division component center for the mitotic poles (Schatten, 2008 [43])
Glioma (KEGG: hsa05214)	43 (14)	3.03	*CAMK2G, PRKCB, PDGFRB, PTEN, RAF1, CDK6, CAMK2A, EGFR, PIK3CG, CAMK2B, CALML5, PRKCA, E2F3, PIK3CA*	Gene pathway is involved with one of the most common primary brain tumors, glioma (Gladson et al., 2010 [44])

^1^ Database source; GO = Gene Ontology, KEGG = Kyoto Encyclopedia of Genes and Genomes, and R = Reactome. ^2^ Number of genes associated with SA. ^3^ Number of leading-edge genes (LEGs) associated with SA in the gene-set. Leading-edge genes are those genes in the gene-set that contribute positively to the normalized enrichment score. ^4^ NES is the normalized enrichment score, which is the enrichment score adjusted by the number of genes in the gene-set.

**Table 3:** Canonical pathways associated with spontaneous abortion identified by ingenuity pathway analysis with inputs of positional candidates and leading-edge genes associated with spontaneous abortion in two or more cattle populations.

Ingenuity Canonical Pathways ^1^	Population	B–H *p-*Value ^2^	Number of Target Molecules ^3^
Synaptic Long Term Potentiation	Cows Heifers and Cows	7.41 × 10^−5^ 2.00 × 10^−31^	5 21
Phospholipases	Cows Heifers and Cows	6.15 × 10^−5^ 8.32 × 10^−4^	4 3
Antioxidant Action of Vitamin C	Cows Heifers and Cows	1.82 × 10^−4^ 3.39 × 10^−3^	4 3
Endothelin-1 Signaling	Cows Heifers and Cows	1.17 × 10^−3^ 3.98 × 10^−16^	4 14
Role of MAPK Signaling in the Pathogenesis of Influenza	Cows Heifers and Cows	1.23 × 10^−3^ 3.89 × 10^−6^	3 5
Sperm Motility	Cows Heifers and Cows	1.23 × 10^−3^ 3.16 × 10^−11^	4 11

^1^ Name of canonical pathways identified by ingenuity pathway analysis for fetal loss in Holstein cattle. ^2^ Significance expressed as a Benjamini–Hochberg (B–H) corrected *p*-value for an association with spontaneous abortion. ^3^ Positional candidate genes from the genome-wide association analysis and leading-edge genes from the gene-set enrichment analysis–SNP genes present in the canonical pathway.

**Table 4:** Master regulators identified by ingenuity pathway analysis and shared in two populations with inputs of positional candidates and leading-edge genes associated with spontaneous abortion in Holstein cattle.

Master Regulator^1^	Molecule Type ^2^	Population	*p-*Value ^3^	Positional Candidate and Leading-Edge Genes ^4^ (#) ^5^
FGF3	Growth Factor	Heifers Heifers and Cows	1.07 × 10^−3^ 4.40 × 10^−3^	***ACOT12**, **AFF3**, AURKA, BABAM2, BANP, **CAMK2G**, CENPH, EYA2, **GAB3**, HMG20B, MCM3, PRKCB, **RBL2**, USP15, VPS72* (18) *ADCY2, ADCY9, **AFF3**, CAMK2A, CAMK2B, CAMK2D, **CAMK2G**, CDK1, CDK6, CEP164, CEP70, EGFR, **GAB3**, GNAQ, GRM5, HSP90AA1, ITPR2, PDGFRB, PLA2G5, PLA2G6, PLCB1, PLK4, PPP3CA, PRKCA, PRKCB, PRKX, PTEN, RAF1, RPS6KA2, RPS6KA3, YWHAG* (18)
NMB	Other	Heifers Cows	2.60 × 10^−3^ 2.30 × 10^−3^	***AFF3**, ASCC3, AURKA, BABAM2, BANP, **CAMK2G**, EYA2, GAB3, HMG20B, PRKCB, RRS1, USP15, VPS72* (14) ***AFF3**, DAO, **GAB3**, LIPE, **NCALD**, NCEH1, NEU3, PAFAH1B1, PAFAH1B3, PLCB1, PSPH, SARDH, SMPDL3A* (16)
CAB39	Enzyme	Cows Heifers and Cows	7.00 × 10^−4^ 1.00 × 10^–4^	***AFF3**, CHDH, CTH, ENPP2, LIPE, **NCALD**, NCEH1, PAFAH1B1, PAFAH1B3, PLA2G2A, PLCB1, SARDH, SMPDL3A* (12) ***AFF3**, AKAP9, CALML5, CAMK2A, CAMK2D, CAMK4, CEP164, CEP70, E2F3, GNAQ, GRIA1, GRIN2A, GRIN2B, GRM1, ITPR1, ITPR2, **NCALD**, PIK3CG, PLA2G5, PLA2G6, PLCB1, PLK4, PPP2R1A, PRKX, PTEN, RAF1, RAP1A, RPS6KA2, RPS6KA3, YWHAG* (23)
PLCB4	Enzyme	Cows Heifers and Cows	9.00 × 10^–4^ 7.00 × 10^–3^	*PLCB1* (1) *PLCB1* (1)

^1^ Master regulators are molecules that indirectly control multiple genes in a pathway within the ingenuity pathway analysis. ^2^ Molecule type of the regulator as defined by the ingenuity pathway analysis. ^3^ Network bias corrected *p*-value calculated by ingenuity pathway analysis. ^4^ List of the positional candidate genes from the genome-wide association analysis (in bold) and leading-edge genes from the gene-set enrichment analysis–SNP regulated by the master regulator. ^5^ Total number of genes regulated by each master regulator.

**Table 5:** Positional candidate and leading-edge genes associated with spontaneous abortion that are shared with previous studies.

Previous Study ^1^	Phenotype ^2^	Breed ^3^	Shared Genes ^4^
[17]	HF pregnant vs. HF open endometrium	Angus crossbred heifers	***AFF3**, GRIN2A, CAMK4, RPS6KA2, PDGFRB, ENPP2, PAFAH2, SMPDL3A, PSPH, SARDH, DAO, BANP, OBFC1*
	SF pregnant vs. SF open endometrium	Angus crossbred heifers	***LOCI100300806**, RPS6KA3, PLCB1, ENPP2, SARDH*
	HF vs. SF conceptus	Angus crossbred heifers	*MNAT1, CAMK4, RPS6KA2, ADCY9, EGFR, E2F3, OBFC1, EYA2, ENPP2*
	HF open endometrium vs. IF open endometrium	Angus crossbred heifers	***LOCI100300806***
[16]	HF vs. SF pregnancy establishment	Angus crossbred heifers	*PLA2G5, PLA2G6, RAF1, PIK3CA, ENPP2, PLA2G2A, PAFAH1B1, PRKCB*
[18]	HF vs. SF endometrial biopsies	Angus crossbred heifers	***LOCI100300806***
	SF vs. IF endometrial biopsies	Angus crossbred heifers	***LOCI100300806***
[73]	Fertil– and Fertil +	Holstein cows	*LIPE*
[74]	HF vs. SF endometrium (immune response)	Holstein heifers	*ADCY2, ITPR1, RAP1A, PPP3CA, ITPR2, CAMK2D, **CAMK2G**, PRKCA, PIK3CA*
	HF vs. SF endometrium (carbuncular endometrium biopsy)	Holstein heifers	*PIK3CG*

^1^ Authors of external study investigating fertility in cattle populations. ^2^ Phenotype of external study used for comparison of identified genes. Phenotypes included high fertile (HF), sub fertile (SF), and infertile (IF) followed by the tissue the gene expression was tested in, and female fertility (Fertil– and Fertil +) using quantitative trait loci. ^3^ Breed of cattle provided in external studies. ^4^ List of the positional candidate genes from the genome-wide association analysis (in bold) and leading-edge genes from previous studies.

## Supplementary Materials

The following are available online at https://www.mdpi.com/2073-4425/10/12/954/s1, Table S1: Master regulators of positional candidate genes and leading-edge genes associated with spontaneous abortion in the Holstein heifer population., Table S2: Canonical pathways identified by ingenuity pathway analysis with inputs of positional candidate genes and leading edge genes associated with spontaneous abortion in a Holstein primiparous cow population, Table S3: Master regulators of positional candidate genes and leading edge genes associated with spontaneous abortion in the Holstein cow population, Table S4. Canonical pathways associated with spontaneous abortion for the combined Holstein heifer and primiparous cow population, Table S5: Upstream regulators identified by ingenuity pathway analysis with inputs of positional candidates and leading edge genes associated with spontaneous abortion in the combined Holstein heifer and cow population, Table S6: Master regulators identified by ingenuity pathway analysis with inputs of positional candidate genes and leading edge genes associated with spontaneous abortion in a combined Holstein heifer and cow population.
