# Supplementary material for: Genomic Analysis of Spontaneous Abortion in Holstein Heifers and Primiparous Cows
Source: Genes (Basel). 2019 Nov 21;10(12):954. doi: 10.3390/genes10120954 (PMC6969913; doi:10.3390/genes10120954)
Supplement: Supplementary file 1 [file genes-10-00954-s001.zip › Supplemental Tables/Supplemental table 4- Combined Population Pathways.docx]

**Table S4.** Canonical pathways associated with spontaneous abortion for the combined Holstein heifer and primiparous cow population.

| **Ingenuity Canonical Pathways^1^** | **BH *P* –value^2^** | **Positional Candidate and Leading Edge Genes^3^** |
| --- | --- | --- |
| Synaptic Long Term Potentiation | 2.00 × 10^-31^ | *GRIN2B, RAF1, GRIN2A, CALML5, CAMK4, GRM1, ITPR2, GRIA1, GNAQ, ITPR1, PPP3CC, RAP1A, GRM5, CAMK2D, CAMK2A, PLCB1, PPP3CA, CAMK2B, PRKCA,* ***CAMK2G****, PRKCB* |
| CREB Signaling in Neurons | 2.51 × 10^-30^ | *GRIN2B, RAF1, ADCY2, PIK3CA, GRIN2A, CALML5, CAMK4, GRM1, ITPR2, GRIA1, GNAQ, ITPR1, RAP1A, GRM5, ADCY9, CAMK2D, CAMK2A, PIK3CG, PLCB1, CAMK2B, PRKCA,* ***CAMK2G****, PRKCB* |
| Opioid Signaling Pathway | 2.00 × 10^-27^ | *GRIN2B, RAF1, ADCY2, GRIN2A, CALML5, CAMK4, ITPR2, RPS6KA3, ITPR1, PPP3CC, RAP1A, ADCY9, CAMK2D, CAMK2A, PIK3CG, PLCB1, RPS6KA2, PPP3CA, CAMK2B, PRKCA,* ***CAMK2G****, PRKCB* |
| Breast Cancer Regulation by Stathmin1 | 3.98 × 10^-27^ | *RAF1, ADCY2, PIK3CA, CALML5, CAMK4, ITPR2, GNAQ, ITPR1, E2F3, RAP1A, CDK1, ADCY9, PPP2R1A, CAMK2D, CAMK2A, PIK3CG, PLCB1, CAMK2B, PRKCA,* ***CAMK2G****, PRKCB* |
| Role of NFAT in Cardiac Hypertrophy | 7.94 × 10^-25^ | *RAF1, ADCY2, PIK3CA, CALML5, CAMK4, ITPR2, GNAQ, ITPR1, PPP3CC, RAP1A, ADCY9, CAMK2D, CAMK2A, PIK3CG, PLCB1, PPP3CA, CAMK2B, PRKCA,* ***CAMK2G****, PRKCB* |
| Neuropathic Pain Signaling In Dorsal Horn Neurons | 7.94 × 10^-25^ | *GRIN2B, PIK3CA, GRIN2A, CAMK4, GRM1, ITPR2, GRIA1, ITPR1, GRM5, CAMK2A, CAMK2D, PIK3CG, PLCB1, CAMK2B, PRKCA,* ***CAMK2G****, PRKCB* |
| Glioma Signaling | 2.51 × 10^-24^ | *RAF1, PIK3CA, CALML5, CAMK4, CDK6, E2F3, RAP1A, PTEN, CAMK2A, CAMK2D, PIK3CG, CAMK2B, EGFR, PDGFRB, PRKCA,* ***CAMK2G****, PRKCB* |
| PI3K Signaling in B Lymphocytes | 1.26 × 10^-23^ | *RAF1, PIK3CA, CALML5, CAMK4, ITPR2, ITPR1, PPP3CC, RAP1A, PTEN, CAMK2A, CAMK2D, PIK3CG, PLCB1, PPP3CA, CAMK2B,* ***CAMK2G****, PRKCB* |
| GNRH Signaling | 6.31 × 10^-22^ | *RAF1, ADCY2, CALML5, CAMK4, ITPR2, GNAQ, ITPR1, RAP1A, ADCY9, CAMK2A, CAMK2D, PLCB1, CAMK2B, EGFR, PRKCA,* ***CAMK2G****, PRKCB* |
| Thrombin Signaling | 7.94 × 10^-22^ | *RAF1, ADCY2, PIK3CA, CAMK4, ITPR2, GNAQ, ITPR1, RAP1A, ADCY9, CAMK2A, CAMK2D, PIK3CG, PLCB1, CAMK2B, EGFR, PRKCA,* ***CAMK2G****, PRKCB* |
| Protein Kinase A Signaling | 7.94 × 10^-22^ | *RAF1, ADCY2, CALML5, CAMK4, YWHAG, ITPR2, AKAP9, GNAQ, ITPR1, PPP3CC, RAP1A, PTEN, ADCY9, CAMK2D, CAMK2A, PLCB1, PPP3CA, CAMK2B, PRKCA,* ***CAMK2G****, PRKCB* |
| Cardiac Hypertrophy Signaling (Enhanced) | 6.31 × 10^-20^ | *RAF1, ADCY2, PIK3CA, CALML5, CAMK4, ITPR2, GNAQ, ITPR1, PPP3CC, RAP1A, PTEN, ADCY9, CAMK2D, CAMK2A, PIK3CG, PLCB1, PPP3CA, CAMK2B, PRKCA,* ***CAMK2G****, PRKCB* |
| Chemokine Signaling | 1.26 × 10^-19^ | *RAF1, CALML5, CAMK4, GNAQ, RAP1A, CAMK2D, CAMK2A, PIK3CG, PLCB1,* ***CAMK2G****, PRKCB, CAMK2B, PRKCA* |
| Phospholipase C Signaling | 3.98 × 10^-19^ | *RAF1, ADCY2, CALML5, CAMK4, ITPR2, GNAQ, RPS6KA3, ITPR1, PPP3CC, RAP1A, ADCY9, PLA2G6, PLA2G5, PLCB1, PPP3CA, PRKCA, PRKCB* |
| Dopamin × 10^-^DARPP32 Feedback in cAMP Signaling | 5.01 × 10^-19^ | *GRIN2B, ADCY2, CALML5, GRIN2A, CAMK4, ITPR2, GNAQ, ITPR1, PPP3CC, ADCY9, PPP2R1A, PLCB1, PPP3CA, PRKCB, PRKCA* |
| Gap Junction Signaling | 5.01 × 10^-19^ | *RAF1, ADCY2, PIK3CA, ITPR2, GRIA1, GNAQ, ITPR1, PPP3CC, RAP1A, ADCY9, PIK3CG, PLCB1, PPP3CA, PRKCA, EGFR, PRKCB* |
| Synaptogenesis Signaling Pathway | 5.01 × 10^-19^ | *GRIN2B, RAF1, ADCY2, PIK3CA, GRIN2A, CALML5, CAMK4, GRM1, GRIA1, ITPR1, RAP1A, GRM5, ADCY9, CAMK2D, CAMK2A, PIK3CG, CAMK2B,* ***CAMK2G*** |
| GÎ±q Signaling | 1.00 × 10^-18^ | *RAF1, CALML5, PIK3CA, CAMK4, GRM1, ITPR2, GNAQ, ITPR1, PPP3CC, GRM5, PIK3CG, PLCB1, PPP3CA, PRKCB, PRKCA* |
| G-Protein Coupled Receptor Signaling | 1.58 × 10^-18^ | *RAF1, ADCY2, PIK3CA, CAMK4, GRM1, GNAQ, RAP1A, GRM5, ADCY9, CAMK2A, CAMK2D, PIK3CG, PLCB1, CAMK2B, PRKCA,* ***CAMK2G****, PRKCB* |
| iCOS-iCOSL Signaling in T Helper Cells | 3.16 × 10^-17^ | *CALML5, PIK3CA, CAMK4, ITPR2, ITPR1, PPP3CC, PTEN, CAMK2D, CAMK2A, PIK3CG, PPP3CA,* ***CAMK2G****, CAMK2B* |
| fMLP Signaling in Neutrophils | 6.31 × 10^-17^ | *RAF1, CALML5, PIK3CA, CAMK4, ITPR2, ITPR1, PPP3CC, RAP1A, PIK3CG, PLCB1, PPP3CA, PRKCB, PRKCA* |
| CCR3 Signaling in Eosinophils | 1.26 × 10^-16^ | *RAF1, PIK3CA, CALML5, CAMK4, ITPR2, ITPR1, RAP1A, PLA2G6, PIK3CG, PLA2G5, PLCB1, PRKCA, PRKCB* |
| Synaptic Long Term Depression | 1.58 × 10^-16^ | *RAF1, GRM1, ITPR2, GRIA1, GNAQ, ITPR1, RAP1A, GRM5, PLA2G6, PPP2R1A, PLA2G5, PLCB1, PRKCB, PRKCA* |
| Melatonin Signaling | 1.58 × 10^-16^ | *RAF1, CALML5, CAMK2D, CAMK4, CAMK2A, GNAQ, PLCB1, CAMK2B, PRKCA,* ***CAMK2G****, PRKCB* |
| B Cell Receptor Signaling | 2.51 × 10^-16^ | *RAF1, CALML5, PIK3CA, CAMK4, PPP3CC, RAP1A, PTEN, CAMK2D, CAMK2A, PIK3CG, PPP3CA, CAMK2B,* ***CAMK2G****, PRKCB* |
| Molecular Mechanisms of Cancer | 3.98 × 10^-16^ | *RAF1, ADCY2, PIK3CA, CDK6, GNAQ, E2F3, RAP1A, CDK1, ADCY9, CAMK2A, CAMK2D, PIK3CG, PLCB1, CAMK2B, PRKCA,* ***CAMK2G****, PRKCB* |
| Role of Macrophages, Fibroblasts and Endothelial Cells in Rheumatoid Arthritis | 3.98 × 10^-16^ | *RAF1, PIK3CA, CALML5, CAMK4, GNAQ, PPP3CC, RAP1A, CAMK2D, CAMK2A, PIK3CG, PLCB1, PPP3CA, CAMK2B, PRKCA,* ***CAMK2G****, PRKCB* |
| Endothelin-1 Signaling | 3.98 × 10^-16^ | *RAF1, ADCY2, PIK3CA, ITPR2, GNAQ, ITPR1, RAP1A, ADCY9, PLA2G6, PIK3CG, PLA2G5, PLCB1, PRKCB, PRKCA* |
| Calcium Signaling | 3.98 × 10^-16^ | *GRIN2B, CALML5, GRIN2A, CAMK4, ITPR2, GRIA1, ITPR1, PPP3CC, RAP1A, CAMK2D, CAMK2A, PPP3CA, CAMK2B,* ***CAMK2G*** |
| GM-CSF Signaling | 7.94 × 10^-16^ | *RAF1, PIK3CA, CAMK2D, CAMK2A, PIK3CG, PPP3CC, RAP1A, PPP3CA, CAMK2B,* ***CAMK2G****, PRKCB* |
| Endocannabinoid Neuronal Synapse Pathway | 1.58 × 10^-15^ | *GRM5, ADCY9, GRIN2B, GRIN2A, ADCY2, GRM1, GRIA1, GNAQ, PLCB1, ITPR1, PPP3CC, PPP3CA* |
| Î±-Adrenergic Signaling | 3.16 × 10^-15^ | *RAF1, ADCY9, CALML5, ADCY2, CAMK4, ITPR2, GNAQ, ITPR1, RAP1A, PRKCA, PRKCB* |
| nNOS Signaling in Neurons | 1.58 × 10^-14^ | *GRIN2B, GRIN2A, CALML5, CAMK4, CAMK2A, PPP3CC, PPP3CA, PRKCA, PRKCB* |
| cAMP-mediated signaling | 5.01 × 10^-14^ | *RAF1, CALML5, ADCY2, CAMK4, AKAP9, PPP3CC, RAP1A, ADCY9, CAMK2D, CAMK2A, PPP3CA,* ***CAMK2G****, CAMK2B* |
| eNOS Signaling | 6.31 × 10^-14^ | *ADCY9, PIK3CA, CALML5, ADCY2, CAMK4, ITPR2, PIK3CG, GNAQ, HSP90AA1, ITPR1, PRKCA, PRKCB* |
| Glioblastoma Multiforme Signaling | 1.00 × 10^-13^ | *RAF1, PIK3CA, ITPR2, PIK3CG, CDK6, PLCB1, ITPR1, E2F3, RAP1A, EGFR, PDGFRB, PTEN* |
| Apelin Endothelial Signaling Pathway | 1.00 × 10^-13^ | *RAF1, ADCY9, PIK3CA, CALML5, ADCY2, CAMK4, PIK3CG, PLCB1, RAP1A, PRKCA, PRKCB* |
| CXCR4 Signaling | 1.00 × 10^-13^ | *RAF1, ADCY9, PIK3CA, ADCY2, ITPR2, PIK3CG, GNAQ, PLCB1, ITPR1, RAP1A, PRKCA, PRKCB* |
| Renin-Angiotensin Signaling | 1.26 × 10^-13^ | *RAF1, ADCY9, PIK3CA, ADCY2, ITPR2, PIK3CG, GNAQ, ITPR1, RAP1A, PRKCA, PRKCB* |
| Role of NFAT in Regulation of the Immune Response | 2.51 × 10^-13^ | *RAF1, PIK3CA, CALML5, CAMK4, ITPR2, PIK3CG, GNAQ, PLCB1, ITPR1, PPP3CC, RAP1A, PPP3CA* |
| Corticotropin Releasing Hormone Signaling | 2.51 × 10^-13^ | *RAF1, ADCY9, CALML5, ADCY2, CAMK4, ITPR2, GNAQ, ITPR1, RAP1A, PRKCA, PRKCB* |
| p70S6K Signaling | 2.51 × 10^-13^ | *RAF1, PIK3CA, PPP2R1A, YWHAG, PIK3CG, GNAQ, PLCB1, RAP1A, EGFR, PRKCA, PRKCB* |
| Adrenomedullin signaling pathway | 5.01 × 10^-13^ | *RAF1, ADCY9, PIK3CA, CALML5, ADCY2, CAMK4, ITPR2, PIK3CG, GNAQ, PLCB1, ITPR1, RAP1A* |
| Xenobiotic Metabolism Signaling | 1.58 × 10^-12^ | *RAF1, PIK3CA, CAMK4, RAP1A, PPP2R1A, CAMK2A, CAMK2D, PIK3CG, HSP90AA1, CAMK2B, PRKCB,* ***CAMK2G****, PRKCA* |
| Non-Small Cell Lung Cancer Signaling | 3.98 × 10^-12^ | *RAF1, PIK3CA, ITPR2, PIK3CG, CDK6, ITPR1, RAP1A, EGFR, PRKCA* |
| Cardiac Hypertrophy Signaling | 5.01 × 10^-12^ | *RAF1, ADCY9, PIK3CA, CALML5, ADCY2, CAMK4, PIK3CG, GNAQ, PLCB1, PPP3CC, RAP1A, PPP3CA* |
| Role of Tissue Factor in Cancer | 5.01 × 10^-12^ | *PIK3CA, PIK3CG, GNAQ, RPS6KA3, PLCB1, RPS6KA2, RAP1A, EGFR, PTEN, PRKCA* |
| P2Y Purigenic Receptor Signaling Pathway | 1.00 × 10^-11^ | *RAF1, ADCY9, PIK3CA, ADCY2, PIK3CG, GNAQ, PLCB1, RAP1A, PRKCA, PRKCB* |
| Calcium-induced T Lymphocyte Apoptosis | 2.00 × 10^-11^ | *CALML5, CAMK4, ITPR2, ITPR1, PPP3CC, PPP3CA, PRKCB, PRKCA* |
| Sperm Motility | 3.16 × 10^-11^ | *PLA2G6, CALML5, CAMK4, ITPR2, PLA2G5, PLCB1, ITPR1, EGFR, PDGFRB, PRKCA, PRKCB* |
| Nitric Oxide Signaling in the Cardiovascular System | 3.98 × 10^-11^ | *PIK3CA, CALML5, CAMK4, ITPR2, PIK3CG, HSP90AA1, ITPR1, PRKCA, PRKCB* |
| Cholecystokinin/Gastrin-mediated Signaling | 6.31 × 10^-11^ | *RAF1, ITPR2, GNAQ, PLCB1, ITPR1, RAP1A, EGFR, PRKCA, PRKCB* |
| Huntington's Disease Signaling | 1.00 × 10^-10^ | *GRM5, GRIN2B, PIK3CA, GRM1, PIK3CG, GNAQ, PLCB1, ITPR1, EGFR, PRKCA, PRKCB* |
| IL-3 Signaling | 3.02 × 10^-10^ | *RAF1, PIK3CA, PIK3CG, PPP3CC, RAP1A, PPP3CA, PRKCB, PRKCA* |
| ERK/MAPK Signaling | 3.24 × 10^-10^ | *RAF1, PLA2G6, PIK3CA, PPP2R1A, YWHAG, PIK3CG, PLA2G5, RAP1A, PRKCA, PRKCB* |
| Glutamate Receptor Signaling | 3.89 × 10^-10^ | *GRM5, GRIN2B, GRIN2A, CALML5, CAMK4, GRM1, GRIA1* |
| VEGF Family Ligand-Receptor Interactions | 4.79 × 10^-10^ | *PLA2G6, RAF1, PIK3CA, PIK3CG, PLA2G5, RAP1A, PRKCB, PRKCA* |
| Melanocyte Development and Pigmentation Signaling | 9.55 × 10^-10^ | *RAF1, ADCY9, ADCY2, PIK3CA, PIK3CG, RPS6KA3, RPS6KA2, RAP1A* |
| PKCÎ¸ Signaling in T Lymphocytes | 1.26 × 10^-9^ | *PIK3CA, CAMK2D, CAMK2A, PIK3CG, PPP3CC, RAP1A, PPP3CA, CAMK2B,* ***CAMK2G*** |
| GPCR-Mediated Nutrient Sensing in Enteroendocrine Cells | 1.26 × 10^-9^ | *ADCY9, ADCY2, ITPR2, GNAQ, PLCB1, ITPR1, PRKCB, PRKCA* |
| UVA-Induced MAPK Signaling | 1.38 × 10^-9^ | *PIK3CA, PIK3CG, RPS6KA3, PLCB1, RPS6KA2, RAP1A, EGFR, PRKCA* |
| Aldosterone Signaling in Epithelial Cells | 1.48 × 10^-9^ | *RAF1, PIK3CA, ITPR2, PIK3CG, PLCB1, HSP90AA1, ITPR1, PRKCA, PRKCB* |
| EGF Signaling | 1.51 × 10^-9^ | *RAF1, PIK3CA, ITPR2, PIK3CG, ITPR1, EGFR, PRKCA* |
| T Cell Receptor Signaling | 2.00 × 10^-9^ | *RAF1, CALML5, PIK3CA, CAMK4, PIK3CG, PPP3CC, RAP1A, PPP3CA* |
| G Beta Gamma Signaling | 2.24 × 10^-9^ | *RAF1, ADCY2, PIK3CG, GNAQ, RAP1A, EGFR, PRKCA, PRKCB* |
| PPARÎ±/RXRÎ± Activation | 2.95 × 10^-9^ | *RAF1, ADCY9, ADCY2, GNAQ, PLCB1, HSP90AA1, RAP1A, PRKCA, PRKCB* |
| Fc Epsilon RI Signaling | 3.63 × 10^-9^ | *PLA2G6, RAF1, PIK3CA, PIK3CG, PLA2G5, RAP1A, PRKCB, PRKCA* |
| PI3K/AKT Signaling | 4.07 × 10^-9^ | *RAF1, PPP2R1A, PIK3CA, YWHAG, PIK3CG, HSP90AA1, RAP1A, PTEN* |
| CD28 Signaling in T Helper Cells | 4.47 × 10^-9^ | *CALML5, PIK3CA, CAMK4, ITPR2, PIK3CG, ITPR1, PPP3CC, PPP3CA* |
| 14-3-3-mediated Signaling | 7.08 × 10^-9^ | *RAF1, PIK3CA, YWHAG, PIK3CG, PLCB1, RAP1A, PRKCA, PRKCB* |
| Neuregulin Signaling | 1.29 × 10^-8^ | *RAF1, HSP90AA1, RAP1A, PRKCB, PRKCA, PTEN, EGFR* |
| HER-2 Signaling in Breast Cancer | 1.58 × 10^-8^ | *PIK3CA, PIK3CG, CDK6, RAP1A, EGFR, PRKCA, PRKCB* |
| PDGF Signaling | 1.78 × 10^-8^ | *RAF1, PIK3CA, PIK3CG, RAP1A, PDGFRB, PRKCA, PRKCB* |
| RANK Signaling in Osteoclasts | 2.14 × 10^-8^ | *RAF1, CALML5, PIK3CA, CAMK4, PIK3CG, PPP3CC, PPP3CA* |
| ErbB Signaling | 2.95 × 10^-8^ | *RAF1, PIK3CA, PIK3CG, RAP1A, EGFR, PRKCA, PRKCB* |
| UVB-Induced MAPK Signaling | 5.37 × 10^-8^ | *PIK3CA, PIK3CG, RPS6KA3, PRKCB, EGFR, PRKCA* |
| RAR Activation | 6.31 × 10^-8^ | *ADCY9, PIK3CA, ADCY2, PIK3CG, MNAT1, PTEN, PRKCA, PRKCB* |
| Telomerase Signaling | 6.31 × 10^-8^ | *RAF1, PIK3CA, PPP2R1A, PIK3CG, HSP90AA1, RAP1A, EGFR* |
| ERK5 Signaling | 7.41 × 10^-8^ | *YWHAG, GNAQ, RPS6KA3, RPS6KA2, RAP1A, EGFR* |
| PTEN Signaling | 7.59 × 10^-8^ | *RAF1, PIK3CA, PIK3CG, RAP1A, EGFR, PDGFRB, PTEN* |
| GPCR-Mediated Integration of Enteroendocrine Signaling Exemplified by an L Cell | 7.76 × 10^-8^ | *ADCY9, ADCY2, ITPR2, GNAQ, PLCB1, ITPR1* |
| CNTF Signaling | 8.51 × 10^-8^ | *RAF1, PIK3CA, PIK3CG, RPS6KA3, RPS6KA2, RAP1A* |
| Thrombopoietin Signaling | 9.12 × 10^-8^ | *RAF1, PIK3CA, PIK3CG, RAP1A, PRKCB, PRKCA* |
| Cellular Effects of Sildenafil (Viagra) | 9.55 × 10^-8^ | *ADCY9, ADCY2, CALML5, CAMK4, ITPR2, PLCB1, ITPR1* |
| Sphingosin × 10^-^1-phosphate Signaling | 1.10 × 10^-7^ | *ADCY9, PIK3CA, ADCY2, PIK3CG, GNAQ, PLCB1, PDGFRB* |
| GP6 Signaling Pathway | 1.15 × 10^-7^ | *PIK3CA, CALML5, CAMK4, PIK3CG, ITPR1, PRKCA, PRKCB* |
| Androgen Signaling | 1.20 × 10^-7^ | *CALML5, CAMK4, GNAQ, HSP90AA1, MNAT1, PRKCA, PRKCB* |
| ErbB4 Signaling | 1.58 × 10^-7^ | *RAF1, PIK3CA, PIK3CG, RAP1A, PRKCB, PRKCA* |
| mTOR Signaling | 1.74 × 10^-7^ | *PIK3CA, PPP2R1A, PIK3CG, RPS6KA3, RPS6KA2, RAP1A, PRKCA, PRKCB* |
| Neuroinflammation Signaling Pathway | 1.74 × 10^-7^ | *PLA2G6, GRIN2B, GRIN2A, PIK3CA, GRIA1, PIK3CG, PLA2G5, PPP3CC, PPP3CA* |
| Growth Hormone Signaling | 1.86 × 10^-7^ | *PIK3CA, PIK3CG, RPS6KA3, RPS6KA2, PRKCB, PRKCA* |
| GDNF Family Ligand-Receptor Interactions | 2.00 × 10^-7^ | *RAF1, PIK3CA, ITPR2, PIK3CG, ITPR1, RAP1A* |
| Regulation of IL-2 Expression in Activated and Anergic T Lymphocytes | 2.29 × 10^-7^ | *RAF1, CALML5, CAMK4, PPP3CC, RAP1A, PPP3CA* |
| Erythropoietin Signaling | 2.57 × 10^-7^ | *RAF1, PIK3CA, PIK3CG, RAP1A, PRKCB, PRKCA* |
| Prolactin Signaling | 3.09 × 10^-7^ | *RAF1, PIK3CA, PIK3CG, RAP1A, PRKCB, PRKCA* |
| FLT3 Signaling in Hematopoietic Progenitor Cells | 3.24 × 10^-7^ | *RAF1, PIK3CA, PIK3CG, RPS6KA3, RPS6KA2, RAP1A* |
| LPS-stimulated MAPK Signaling | 3.80 × 10^-7^ | *RAF1, PIK3CA, PIK3CG, RAP1A, PRKCB, PRKCA* |
| NF-ÎºB Activation by Viruses | 3.80 × 10^-7^ | *RAF1, PIK3CA, PIK3CG, RAP1A, PRKCB, PRKCA* |
| UVC-Induced MAPK Signaling | 4.68 × 10^-7^ | *RAF1, RAP1A, PRKCB, EGFR, PRKCA* |
| Prostate Cancer Signaling | 6.03 × 10^-7^ | *RAF1, PIK3CA, PIK3CG, HSP90AA1, RAP1A, PTEN* |
| FAK Signaling | 7.41 × 10^-7^ | *RAF1, PIK3CA, PIK3CG, RAP1A, EGFR, PTEN* |
| Axonal Guidance Signaling | 7.76 × 10^-7^ | *RAF1, PIK3CA, PIK3CG, GNAQ, PLCB1, PPP3CC, RAP1A, PPP3CA, PRKCA, PRKCB* |
| Cancer Drug Resistance By Drug Efflux | 7.76 × 10^-7^ | *RAF1, PIK3CA, PIK3CG, RAP1A, PTEN* |
| Amyotrophic Lateral Sclerosis Signaling | 8.51 × 10^-7^ | *GRIN2B, GRIN2A, PIK3CA, GRIA1, PIK3CG, PPP3CA* |
| VEGF Signaling | 8.71 × 10^-7^ | *RAF1, PIK3CA, PIK3CG, RAP1A, PRKCB, PRKCA* |
| Apelin Cardiomyocyte Signaling Pathway | 9.12 × 10^-7^ | *PIK3CA, PIK3CG, PLCB1, ITPR1, PRKCB, PRKCA* |
| Chronic Myeloid Leukemia Signaling | 9.55 × 10^-7^ | *RAF1, PIK3CA, PIK3CG, CDK6, E2F3, RAP1A* |
| NF-ÎºB Signaling | 9.55 × 10^-7^ | *RAF1, PIK3CA, PIK3CG, RAP1A, EGFR, PDGFRB, PRKCB* |
| HGF Signaling | 1.38 × 10^-6^ | *RAF1, PIK3CA, PIK3CG, RAP1A, PRKCB, PRKCA* |
| Melanoma Signaling | 1.41 × 10^-6^ | *RAF1, PIK3CA, PIK3CG, RAP1A, PTEN* |
| NGF Signaling | 1.66 × 10^-6^ | *RAF1, PIK3CA, PIK3CG, RPS6KA3, RPS6KA2, RAP1A* |
| Endocannabinoid Developing Neuron Pathway | 1.86 × 10^-6^ | *RAF1, ADCY9, ADCY2, PIK3CA, PIK3CG, RAP1A* |
| Natural Killer Cell Signaling | 2.04 × 10^-6^ | *RAF1, PIK3CA, PIK3CG, RAP1A, PRKCB, PRKCA* |
| IL-8 Signaling | 2.04 × 10^-6^ | *RAF1, PIK3CA, PIK3CG, RAP1A, EGFR, PRKCA, PRKCB* |
| Endometrial Cancer Signaling | 2.57 × 10^-6^ | *RAF1, PIK3CA, PIK3CG, RAP1A, PTEN* |
| ErbB2-ErbB3 Signaling | 3.55 × 10^-6^ | *RAF1, PIK3CA, PIK3CG, RAP1A, PTEN* |
| Cyclins and Cell Cycle Regulation | 3.72 × 10^-6^ | *RAF1, PPP2R1A, CDK6, E2F3, CDK1* |
| Role of MAPK Signaling in the Pathogenesis of Influenza | 3.89 × 10^-6^ | *PLA2G6, RAF1, PLA2G5, RAP1A, PRKCA* |
| Superpathway of Inositol Phosphate Compounds | 4.07 × 10^-6^ | *PIK3CA, PIK3CG, PLCB1, PPP3CA, PTEN, PDGFRB, EGFR* |
| Glucocorticoid Receptor Signaling | 4.27 × 10^-6^ | *RAF1, PIK3CA, PIK3CG, HSP90AA1, PPP3CC, MNAT1, RAP1A, PPP3CA* |
| SPINK1 General Cancer Pathway | 4.57 × 10^-6^ | *RAF1, PIK3CA, PIK3CG, RAP1A, EGFR* |
| Hereditary Breast Cancer Signaling | 4.57 × 10^-6^ | *PIK3CA, PIK3CG, CDK6, RAP1A, CDK1, PTEN* |
| Ovarian Cancer Signaling | 4.57 × 10^-6^ | *RAF1, PIK3CA, PIK3CG, RAP1A, EGFR, PTEN* |
| Leptin Signaling in Obesity | 5.25 × 10^-6^ | *ADCY9, ADCY2, PIK3CA, PIK3CG, PLCB1* |
| Endocannabinoid Cancer Inhibition Pathway | 5.25 × 10^-6^ | *RAF1, ADCY9, ADCY2, PIK3CA, PIK3CG, GNAQ* |
| Relaxin Signaling | 6.03 × 10^-6^ | *ADCY9, ADCY2, PIK3CA, PIK3CG, GNAQ, RAP1A* |
| FGF Signaling | 6.31 × 10^-6^ | *RAF1, PIK3CA, PIK3CG, ITPR1, PRKCA* |
| Macropinocytosis Signaling | 6.31 × 10^-6^ | *PIK3CA, PIK3CG, RAP1A, PRKCB, PRKCA* |
| FcÎ³ Receptor-mediated Phagocytosis in Macrophages and Monocytes | 6.92 × 10^-6^ | *PLA2G6, PIK3CG, PRKCB, PTEN, PRKCA* |
| JAK/Stat Signaling | 6.92 × 10^-6^ | *RAF1, PIK3CA, PIK3CG, GNAQ, RAP1A* |
| Ephrin Receptor Signaling | 1.02 × 10^-5^ | *RAF1, GRIN2B, GRIN2A, PIK3CG, GNAQ, RAP1A* |
| Ceramide Signaling | 1.12 × 10^-5^ | *RAF1, PPP2R1A, PIK3CA, PIK3CG, RAP1A* |
| CDK5 Signaling | 1.35 × 10^-5^ | *RAF1, ADCY9, PPP2R1A, ADCY2, RAP1A* |
| PAK Signaling | 1.58 × 10^-5^ | *RAF1, PIK3CA, PIK3CG, RAP1A, PDGFRB* |
| Regulation of the Epithelial-Mesenchymal Transition Pathway | 1.86 × 10^-5^ | *RAF1, PIK3CA, PIK3CG, RAP1A, EGFR, PDGFRB* |
| IGF-1 Signaling | 1.86 × 10^-5^ | *RAF1, PIK3CA, YWHAG, PIK3CG, RAP1A* |
| Production of Nitric Oxide and Reactive Oxygen Species in Macrophages | 2.00 × 10^-5^ | *PPP2R1A, PIK3CA, PIK3CG, RAP1A, PRKCB, PRKCA* |
| 3-phosphoinositide Biosynthesis | 2.04 × 10^-5^ | *PIK3CA, PIK3CG, PPP3CA, EGFR, PDGFRB, PTEN* |
| NRF2-mediated Oxidative Stress Response | 2.09 × 10^-5^ | *RAF1, PIK3CA, PIK3CG, RAP1A, PRKCB, PRKCA* |
| Virus Entry via Endocytic Pathways | 2.24 × 10^-5^ | *PIK3CA, PIK3CG, RAP1A, PRKCB, PRKCA* |
| Pancreatic Adenocarcinoma Signaling | 2.51 × 10^-5^ | *RAF1, PIK3CA, PIK3CG, E2F3, EGFR* |
| Nur77 Signaling in T Lymphocytes | 2.57 × 10^-5^ | *CALML5, CAMK4, PPP3CC, PPP3CA* |
| Wnt/Ca+ pathway | 3.09 × 10^-5^ | *CAMK2A, PLCB1, PPP3CA, PRKCA* |
| Mitotic Roles of Polo-Like Kinase | 3.89 × 10^-5^ | *PLK4, PPP2R1A, HSP90AA1, CDK1* |
| Regulation of Cellular Mechanics by Calpain Protease | 3.89 × 10^-5^ | *CDK6, RAP1A, CDK1, EGFR* |
| Role of Osteoblasts, Osteoclasts and Chondrocytes in Rheumatoid Arthritis | 4.27 × 10^-5^ | *PIK3CA, CALML5, CAMK4, PIK3CG, PPP3CC, PPP3CA* |
| Phagosome Formation | 4.27 × 10^-5^ | *PIK3CA, PIK3CG, PLCB1, PRKCB, PRKCA* |
| Insulin Receptor Signaling | 6.03 × 10^-5^ | *RAF1, PIK3CA, PIK3CG, RAP1A, PTEN* |
| IL-2 Signaling | 6.31 × 10^-5^ | *RAF1, PIK3CA, PIK3CG, RAP1A* |
| Myc Mediated Apoptosis Signaling | 8.13 × 10^-5^ | *PIK3CA, YWHAG, PIK3CG, RAP1A* |
| Colorectal Cancer Metastasis Signaling | 8.32 × 10^-5^ | *ADCY9, ADCY2, PIK3CA, PIK3CG, RAP1A, EGFR* |
| Regulation of eIF4 and p70S6K Signaling | 1.00 × 10^-4^ | *RAF1, PPP2R1A, PIK3CA, PIK3CG, RAP1A* |
| IL-15 Signaling | 1.10 × 10^-4^ | *RAF1, PIK3CA, PIK3CG, RAP1A* |
| Small Cell Lung Cancer Signaling | 1.10 × 10^-4^ | *PIK3CA, PIK3CG, CDK6, PTEN* |
| Neurotrophin/TRK Signaling | 1.15 × 10^-4^ | *RAF1, PIK3CA, PIK3CG, RAP1A* |
| Crosstalk between Dendritic Cells and Natural Killer Cells | 1.17 × 10^-4^ | *CAMK2D, CAMK2A,* ***CAMK2G****, CAMK2B* |
| Estrogen-Dependent Breast Cancer Signaling | 1.23 × 10^-4^ | *PIK3CA, PIK3CG, RAP1A, EGFR* |
| Tec Kinase Signaling | 1.32 × 10^-4^ | *PIK3CA, PIK3CG, GNAQ, PRKCB, PRKCA* |
| Renal Cell Carcinoma Signaling | 1.38 × 10^-4^ | *RAF1, PIK3CA, PIK3CG, RAP1A* |
| CCR5 Signaling in Macrophages | 1.41 × 10^-4^ | *CALML5, CAMK4, PRKCB, PRKCA* |
| PEDF Signaling | 1.66 × 10^-4^ | *RAF1, PIK3CA, PIK3CG, RAP1A* |
| Apoptosis Signaling | 1.74 × 10^-4^ | *RAF1, RAP1A, CDK1, PRKCA* |
| Acute Myeloid Leukemia Signaling | 2.00 × 10^-4^ | *RAF1, PIK3CA, PIK3CG, RAP1A* |
| PPAR Signaling | 2.04 × 10^-4^ | *RAF1, HSP90AA1, RAP1A, PDGFRB* |
| Leukocyte Extravasation Signaling | 2.82 × 10^-4^ | *PIK3CA, PIK3CG, RAP1A, PRKCB, PRKCA* |
| Role of IL-17F in Allergic Inflammatory Airway Diseases | 3.09 × 10^-4^ | *RAF1, RPS6KA3, RPS6KA2* |
| Mouse Embryonic Stem Cell Pluripotency | 3.16 × 10^-4^ | *RAF1, PIK3CA, PIK3CG, RAP1A* |
| p38 MAPK Signaling | 3.24 × 10^-4^ | *PLA2G6, PLA2G5, RPS6KA3, RPS6KA2* |
| Integrin Signaling | 3.89 × 10^-4^ | *RAF1, PIK3CA, PIK3CG, RAP1A, PTEN* |
| GÎ±i Signaling | 4.07 × 10^-4^ | *RAF1, ADCY9, ADCY2, RAP1A* |
| Rac Signaling | 4.27 × 10^-4^ | *RAF1, PIK3CA, PIK3CG, RAP1A* |
| HIF1Î± Signaling | 4.47 × 10^-4^ | *PIK3CA, PIK3CG, HSP90AA1, RAP1A* |
| Role of NANOG in Mammalian Embryonic Stem Cell Pluripotency | 5.01 × 10^-4^ | *RAF1, PIK3CA, PIK3CG, RAP1A* |
| STAT3 Pathway | 5.25 × 10^-4^ | *RAF1, RAP1A, PDGFRB, EGFR* |
| Role of CHK Proteins in Cell Cycle Checkpoint Control | 5.89 × 10^-4^ | *PPP2R1A, E2F3, CDK1* |
| IL-6 Signaling | 5.89 × 10^-4^ | *RAF1, PIK3CA, PIK3CG, RAP1A* |
| Cardiac Î²-adrenergic Signaling | 6.03 × 10^-4^ | *ADCY9, PPP2R1A, ADCY2, AKAP9* |
| GÎ±12/13 Signaling | 6.92 × 10^-4^ | *RAF1, PIK3CA, PIK3CG, RAP1A* |
| IL-12 Signaling and Production in Macrophages | 7.24 × 10^-4^ | *PIK3CA, PIK3CG, PRKCA, PRKCB* |
| Gustation Pathway | 8.13 × 10^-4^ | *ADCY9, ADCY2, ITPR2, ITPR1* |
| Pyridoxal 5'-phosphate Salvage Pathway | 8.32 × 10^-4^ | *PRKX, CDK6, CDK1* |
| Phospholipases | 8.32 × 10^-4^ | *PLA2G6, PLA2G5, PLCB1* |
| Type II Diabetes Mellitus Signaling | 8.51 × 10^-4^ | *PIK3CA, PIK3CG, PRKCA, PRKCB* |
| Role of Pattern Recognition Receptors in Recognition of Bacteria and Viruses | 1.17 × 10^-3^ | *PIK3CA, PIK3CG, PRKCA, PRKCB* |
| MSP-RON Signaling Pathway | 1.20 × 10^-3^ | *PIK3CA, PIK3CG, RPS6KA2* |
| Dopamine Receptor Signaling | 1.35 × 10^-3^ | *ADCY9, PPP2R1A, ADCY2* |
| Acute Phase Response Signaling | 1.41 × 10^-3^ | *RAF1, PIK3CA, PIK3CG, RAP1A* |
| IL-17A Signaling in Airway Cells | 1.48 × 10^-3^ | *PIK3CA, PIK3CG, PTEN* |
| Role of JAK1 and JAK3 in Î³c Cytokine Signaling | 1.51 × 10^-3^ | *PIK3CA, PIK3CG, RAP1A* |
| Hepatic Cholestasis | 1.55 × 10^-3^ | *ADCY9, ADCY2, PRKCB, PRKCA* |
| BMP signaling pathway | 1.74 × 10^-3^ | *RAF1, CAMK4, RAP1A* |
| T Cell Exhaustion Signaling Pathway | 1.74 × 10^-3^ | *PIK3CA, PPP2R1A, PIK3CG, RAP1A* |
| Angiopoietin Signaling | 1.95 × 10^-3^ | *PIK3CA, PIK3CG, RAP1A* |
| FcÎ³RIIB Signaling in B Lymphocytes | 2.00 × 10^-3^ | *PIK3CA, PIK3CG, RAP1A* |
| Antiproliferative Role of Somatostatin Receptor 2 | 2.00 × 10^-3^ | *PIK3CA, PIK3CG, RAP1A* |
| Glioma Invasiveness Signaling | 2.00 × 10^-3^ | *PIK3CA, PIK3CG, RAP1A* |
| IL-1 Signaling | 2.04 × 10^-3^ | *ADCY9, ADCY2, GNAQ* |
| ILK Signaling | 2.19 × 10^-3^ | *PIK3CA, PPP2R1A, PIK3CG, PTEN* |
| Clathrin-mediated Endocytosis Signaling | 2.34 × 10^-3^ | *PIK3CA, PIK3CG, PPP3CC, PPP3CA* |
| IL-17 Signaling | 2.34 × 10^-3^ | *PIK3CA, PIK3CG, RAP1A* |
| Bladder Cancer Signaling | 2.40 × 10^-3^ | *RAF1, RAP1A, EGFR* |
| Salvage Pathways of Pyrimidine Ribonucleotides | 2.40 × 10^-3^ | *PRKX, CDK6, CDK1* |
| IL-4 Signaling | 2.51 × 10^-3^ | *PIK3CA, PIK3CG, RAP1A* |
| Estrogen-mediated S-phase Entry | 2.57 × 10^-3^ | *E2F3, CDK1* |
| TR/RXR Activation | 2.57 × 10^-3^ | *PIK3CA, CAMK4, PIK3CG* |
| CTLA4 Signaling in Cytotoxic T Lymphocytes | 2.63 × 10^-3^ | *PIK3CA, PPP2R1A, PIK3CG* |
| GÎ±s Signaling | 3.09 × 10^-3^ | *ADCY9, ADCY2, RAP1A* |
| EIF2 Signaling | 3.31 × 10^-3^ | *RAF1, PIK3CA, PIK3CG, RAP1A* |
| Actin Cytoskeleton Signaling | 3.39 × 10^-3^ | *RAF1, PIK3CA, PIK3CG, RAP1A* |
| Antioxidant Action of Vitamin C | 3.39 × 10^-3^ | *PLA2G6, PLA2G5, PLCB1* |
| G Protein Signaling Mediated by Tubby | 3.55 × 10^-3^ | *GNAQ, PLCB1* |
| p53 Signaling | 3.55 × 10^-3^ | *PIK3CA, PIK3CG, PTEN* |
| Systemic Lupus Erythematosus Signaling | 3.63 × 10^-3^ | *PIK3CA, CAMK4, PIK3CG, RAP1A* |
| SAPK/JNK Signaling | 3.72 × 10^-3^ | *PIK3CA, PIK3CG, RAP1A* |
| Circadian Rhythm Signaling | 3.89 × 10^-3^ | *GRIN2B, GRIN2A* |
| MIF-mediated Glucocorticoid Regulation | 4.17 × 10^-3^ | *PLA2G6, PLA2G5* |
| Paxillin Signaling | 4.47 × 10^-3^ | *PIK3CA, PIK3CG, RAP1A* |
| Signaling by Rho Family GTPases | 4.68 × 10^-3^ | *RAF1, PIK3CA, PIK3CG, GNAQ* |
| Cell Cycle Regulation by BTG Family Proteins | 4.79 × 10^-3^ | *PPP2R1A, E2F3* |
| Mechanisms of Viral Exit from Host Cells | 5.89 × 10^-3^ | *PRKCA, PRKCB* |
| nNOS Signaling in Skeletal Muscle Cells | 5.89 × 10^-3^ | *CALML5, CAMK4* |
| Estrogen Receptor Signaling | 5.89 × 10^-3^ | *RAF1, MNAT1, RAP1A* |
| MIF Regulation of Innate Immunity | 6.03 × 10^-3^ | *PLA2G6, PLA2G5* |
| Oncostatin M Signaling | 6.31 × 10^-3^ | *RAF1, RAP1A* |
| Serotonin Receptor Signaling | 6.31 × 10^-3^ | *ADCY9, ADCY2* |
| iNOS Signaling | 6.92 × 10^-3^ | *CALML5, CAMK4* |
| Role of p14/p19ARF in Tumor Suppression | 6.92 × 10^-3^ | *PIK3CA, PIK3CG* |
| Human Embryonic Stem Cell Pluripotency | 6.92 × 10^-3^ | *PIK3CA, PIK3CG, PDGFRB* |
| IL-9 Signaling | 7.41 × 10^-3^ | *PIK3CA, PIK3CG* |
| Epithelial Adherens Junction Signaling | 7.59 × 10^-3^ | *RAP1A, EGFR, PTEN* |
| Cell Cycle: G2/M DNA Damage Checkpoint Regulation | 7.94 × 10^-3^ | *YWHAG, CDK1* |
| D-myo-inositol-5-phosphate Metabolism | 8.91 × 10^-3^ | *PLCB1, PPP3CA, PTEN* |
| Docosahexaenoic Acid (DHA) Signaling | 9.55 × 10^-3^ | *PIK3CA, PIK3CG* |

^1^Name of canonical pathways identified by Ingenuity Pathway Analysis for late term loss in combined Holstein heifer and primiparous cows.

^2^Significance expressed as a Benjamini-Hochberg corrected *P*-value for an association with late term spontaneous abortion.

^3^ List of the positional candidate genes from the genome-wide association analysis (in **bold**) and leading edge genes from the gene-set enrichment analysis-SNP present in the canonical pathway.
