# Supplementary material for: Genomic Analysis of Spontaneous Abortion in Holstein Heifers and Primiparous Cows
Source: Genes (Basel). 2019 Nov 21;10(12):954. doi: 10.3390/genes10120954 (PMC6969913; doi:10.3390/genes10120954)
Supplement: Supplementary file 1 [file genes-10-00954-s001.zip › Supplemental Tables/Supplemental table 2- Cow Population Pathways.docx]

**Table S2:** Canonical pathways identified by Ingenuity Pathway Analysis with inputs of positional candidate genes and leading edge genes associated with spontaneous abortion in a Holstein primiparous cow population.

| **Ingenuity Canonical Pathways^1^** | **BH *P*-value^2^** | **Positional Candidate and Leading Edge Genes^3^** |
| --- | --- | --- |
| Phospholipases | 6.31 × 10^-5^ | *PLCB1, PAFAH1B1, PLA2G2A, PAFAH1B3* |
| Synaptic Long Term Depression | 7.41 × 10^-5^ | *PLCB1, CTH, PAFAH1B1, PLA2G2A, PAFAH1B3* |
| Antioxidant Action of Vitamin C | 1.82 × 10^-4^ | *PLCB1, PAFAH1B1, PLA2G2A, PAFAH1B3* |
| Endothelin-1 Signaling | 1.17 × 10^-3^ | *PLCB1, PAFAH1B1, PLA2G2A, PAFAH1B3* |
| Eicosanoid Signaling | 1.17 × 10^-3^ | *PAFAH1B1, PLA2G2A, PAFAH1B3* |
| Role of MAPK Signaling in the Pathogenesis of Influenza | 1.23 × 10^-3^ | *PAFAH1B1, PLA2G2A, PAFAH1B3* |
| Sperm Motility | 1.23 × 10^-3^ | *PLCB1, PAFAH1B1, PLA2G2A, PAFAH1B3* |
| Atherosclerosis Signaling | 3.98 × 10^-3^ | *PAFAH1B1, PLA2G2A, PAFAH1B3* |
| Retinol Biosynthesis | 9.55 × 10^-3^ | *PNLIPRP2, LIPE* |
| L-cysteine Degradation II | 9.77 × 10^-3^ | *CTH* |

^1^Name of canonical pathways identified by Ingenuity Pathway Analysis for fetal loss in Holstein cows

^2^Significance expressed as a Benjamini-Hochberg corrected *P*-value for an association with late term spontaneous abortion.

^3^List of the positional candidate genes from the genome-wide association analysis and leading edge genes from the gene-set enrichment analysis-SNP present in the canonical pathway.
