# Supplementary material for: Genomic Analysis of Spontaneous Abortion in Holstein Heifers and Primiparous Cows
Source: Genes (Basel). 2019 Nov 21;10(12):954. doi: 10.3390/genes10120954 (PMC6969913; doi:10.3390/genes10120954)
Supplement: Supplementary file 1 [file genes-10-00954-s001.zip › Supplemental Tables/Supplemental table 6- Combined Population Master Regulators.docx]

**Table S6:** Master regulators identified by Ingenuity Pathway Analysis with inputs of positional candidate genes and leading edge genes associated with spontaneous abortion in a combined Holstein heifer and cow population.

| **Master Regulator^1^** | **Molecule Type^2^** | ***P*-value^3^** | **Positional Candidate and Leading Edge Genes^4^** |
| --- | --- | --- | --- |
| CAB39 | enzyme | 1.00 × 10^-4^ | ***AFF3****, AKAP9, CALML5, CAMK2A, CAMK2D, CAMK4, CEP164, CEP70, E2F3, GNAQ, GRIA1, GRIN2A, GRIN2B, GRM1, ITPR1, ITPR2,* ***NCALD****, PIK3CG, PLA2G5, PLA2G6, PLCB1, PLK4, PPP2R1A, PRKX, PTEN, RAF1, RAP1A, RPS6KA2, RPS6KA3, YWHAG* |
| SLITRK5 | other | 1.00 × 10^-4^ | *GRIA1, GRIN2A, GRIN2B* |
| HOMER2 | other | 1.00 × 10^-4^ | *GRIN2A, GRM1, GRM5* |
| neurotransmitter | chemical - other | 1.00 × 10^-4^ | *CAMK2A, EGFR, GRIA1, GRIN2A* |
| mir-132 | microRNA | 1.00 × 10^-4^ | *GRIA1, GRIN2A, GRIN2B, PTEN* |
| methylanthraniloyl guanosine triphosphate | chemical reagent | 1.00 × 10^-4^ | *ADCY2, ADCY9, GRIA1* |
| 3'-(N-methylanthraniloyl)-2'-deoxy-ATP | chemical reagent | 1.00 × 10^-4^ | *ADCY2, ADCY9, GRIA1* |
| 2'-(or-3')-O-(N-methylanthraniloyl)ATP | chemical reagent | 1.00 × 10^-4^ | *ADCY2, ADCY9, GRIA1* |
| 2'/3'-O-N-methylanthraniloyl-GTP-gamma-S | chemical reagent | 1.00 × 10^-4^ | *ADCY2, ADCY9, GRIA1* |
| 2'/3'-O-N-methylanthraniloyl-ITP-gamma-S | chemical reagent | 1.00 × 10^-4^ | *ADCY2, ADCY9, GRIA1* |
| epalrestat | chemical drug | 1.00 × 10^-4^ | *PDGFRB, PRKCB* |
| ABCB4 | transporter | 2.00 × 10^-4^ | *CDK1, CDK6, E2F3, EGFR, GRIA1, GRIN2A, GRIN2B, GRM1, GRM5, NUMA1, PDGFRB, PIK3CG, PLA2G5, PLA2G6, PPP2R1A, PRKCA, PRKCB, PTEN, RAF1, RAP1A, RPS6KA3* |
| ergotamine | chemical drug | 2.00 × 10^-4^ | *CALML5, CAMK2A, CAMK4, CDK1, CDK6, E2F3, EGFR, GRIA1, GRIN2A, GRM1, ITPR1, ITPR2, PDGFRB, PIK3CA, PIK3CG, PLA2G5, PLCB1, PPP2R1A, PRKCA, PTEN, TUBGCP5, YWHAG* |
| DEVD-FMK | chemical reagent | 2.00 × 10^-4^ | *CAMK2A, CAMK2B, CAMK2D, CAMK4, CDK1, CDK6, CEP164, CEP70, E2F3, GNAQ, GRIN2A, GRM5, ITPR2, PIK3CA, PIK3CG, PLA2G5, PLCB1, PLK4, PPP2R1A, PPP3CA, PRKX, PTEN, RAF1, RAP1A, RPS6KA2* |
| p38 MAP kinase inhibitor | chemical drug | 2.00 × 10^-4^ | *CDK1, CDK6, GRIA1, GRIN2A, GRIN2B, GRM1, GRM5, PTEN, RAF1* |
| L-homocysteic acid | chemical - endogenous mammalian | 2.00 × 10^-4^ | *CAMK2A, EGFR, GRIN2A* |
| miR-132-3p (and other miRNAs w/seed AACAGUC) | mature microRNA | 2.00 × 10^-4^ | *GRIA1, GRIN2A, GRIN2B* |
| BAIAP2 | kinase | 2.00 × 10^-4^ | *GRIN2A, GRIN2B* |
| EID3 | other | 3.00 × 10^-4^ | *CAMK2D,* ***CAMK2G****, CDK1, CDK6, CEP70, EGFR, GNAQ, GRIA1, GRIN2A, GRIN2B, GRM5, HSP90AA1, PLCB1, PLK4, PPP2R1A, PRKX, PTEN, RAF1* |
| FCGR2C | transmembrane receptor | 3.00 × 10^-4^ | ***AFF3****, CAMK2A, CAMK2B, CAMK2D, CDK1, CEP70, E2F3, GNAQ, GRM1, GRM5, HSP90AA1, ITPR1, NUMA1, PIK3CA, PIK3CG, PLA2G5, PLCB1, PLK4, PPP2R1A, PPP3CA, PRKCA, PRKCB, PRKX, RAF1, RAP1A, RPS6KA3* |
| dihydroergotamine | chemical drug | 3.00 × 10^-4^ | *ADCY2, ADCY9, CALML5, CAMK2A, CAMK4, CDK6, EGFR, GRIN2A, GRM1, GRM5, ITPR1, PDGFRB, PLA2G5, PLCB1, PPP2R1A, PRKCA, TUBGCP5, YWHAG* |
| lurasidone | chemical drug | 3.00 × 10^-4^ | *ADCY2, ADCY9, CAMK2A, CDK6, E2F3, EGFR, GRIA1, GRIN2A, GRM1, GRM5, ITPR1, PDGFRB, PIK3CA, PIK3CG, PPP2R1A, PRKCA, PTEN, YWHAG* |
| dihydrexidine | chemical drug | 3.00 × 10^-4^ | *CAMK2A, GRIA1, GRIN2A* |
| NMDA Receptor | complex | 3.00 × 10^-4^ | *CAMK2A, GRIA1, GRIN2A* |
| D-2-amino-5-phosphonovaleric acid | chemical reagent | 3.00 × 10^-4^ | *CAMK2B, GRIN2B* |
| ZNF335 | transcription regulator | 4.00 × 10^-4^ | *CAMK2B, CDK1, CDK6, CEP70, EGFR, GNAQ, GRIA1, GRIN2A, GRIN2B, GRM1, GRM5, HSP90AA1, PLCB1, PLK4, PPP2R1A, PRKX, PTEN, RAF1* |
| aminoglutethimide | chemical drug | 4.00 × 10^-4^ | *CAMK2A, CAMK4, EGFR, GRIA1, GRIN2A, PTEN* |
| MECP2 | transcription regulator | 4.00 × 10^-4^ | *CAMK2A, CAMK2B, GRIA1, GRIN2A* |
| DLG3 | kinase | 4.00 × 10^-4^ | *GRIN2A, GRIN2B* |
| ADCY5 | enzyme | 4.00 × 10^-4^ | *ADCY2, ADCY9* |
| PQR309 | chemical drug | 5.00 × 10^-4^ | ***AFF3****, CAMK2B, CAMK2D, CAMK4, CDK6, CEP70, EGFR, GNAQ, GRIA1, GRM1, GRM5, ITPR2, PDGFRB, PIK3CG, PLA2G6, PLCB1, PLK4, PPP2R1A, PPP3CA, PRKCA, PRKCB, PRKX, PTEN, RAF1, RAP1A, RPS6KA3* |
| SP1 | transcription regulator | 5.00 × 10^-4^ | ***AFF3****, CAMK2B, CAMK2D,* ***CAMK2G****, CDK1, CDK6, CEP70, EGFR, GNAQ, GRIN2A, GRM1, GRM5, ITPR1, NUMA1, PDGFRB, PIK3CA, PLA2G5, PLA2G6, PLCB1, PLK4, PPP2R1A, PRKCA, PRKCB, PRKX, PTEN, RAF1, RPS6KA3, TUBGCP5* |
| PKI-179 | chemical drug | 6.00 × 10^-4^ | ***AFF3****, CAMK2B, CAMK4, CDK6, CEP70, EGFR, GNAQ, GRIA1, GRM1, GRM5, PDGFRB, PIK3CA, PIK3CG, PLA2G6, PLCB1, PLK4, PPP2R1A, PPP3CA, PRKCA, PRKCB, PRKX, PTEN, RAF1, RPS6KA3* |
| PWT33597 | chemical drug | 6.00 × 10^-4^ | ***AFF3****, CAMK2B, CAMK4, CDK6, CEP70, EGFR, GNAQ, GRIA1, GRM1, GRM5, PDGFRB, PIK3CA, PIK3CG, PLA2G6, PLCB1, PLK4, PPP2R1A, PPP3CA, PRKCA, PRKCB, PRKX, PTEN, RAF1, RPS6KA3* |
| N-methyl-(R)-salsolinol | chemical - endogenous mammalian | 6.00 × 10^-4^ | *CAMK4, CDK1, CDK6, PTEN* |
| D-glutamine | chemical - endogenous mammalian | 6.00 × 10^-4^ | *CAMK4, CDK1, CDK6, PTEN* |
| ZDHHC21 | enzyme | 7.00 × 10^-4^ | *CAMK2A, CAMK2B, CAMK2D, CDK1, CEP70, E2F3, GNAQ, GRM1, GRM5, HSP90AA1, ITPR1, NUMA1, PDGFRB, PIK3CA, PIK3CG, PLA2G5, PLA2G6, PLCB1, PLK4, PPP2R1A, PRKCB, PRKX, RAP1A, RPS6KA3* |
| NCR2 | transmembrane receptor | 8.00 × 10^-4^ | ***AFF3****, CALML5, CAMK2A, CAMK2B,* ***CAMK2G****, CDK1, CEP70, E2F3, EGFR, GNAQ, GRIA1, GRM5, HSP90AA1, ITPR1, NUMA1, PIK3CA, PLA2G5, PLCB1, PLK4, PPP2R1A, PPP3CA, PRKCB, PRKX, RAF1, RAP1A, RPS6KA3* |
| nitrite | chemical - endogenous mammalian | 8.00 × 10^-4^ | *CDK1, CDK6, EGFR, PTEN* |
| PDE2A | enzyme | 9.00 × 10^-4^ | *ADCY2, ADCY9, CAMK2B, CAMK2D, CAMK4, CDK1, CDK6, CEP164, CEP70, GNAQ, GRIA1, GRIN2A, GRIN2B, ITPR1, ITPR2, PDGFRB, PIK3CG, PLA2G5, PLA2G6, PLCB1, PLK4, PPP2R1A, PPP3CA, PRKCA, PRKX, RPS6KA2, YWHAG* |
| P2RY12 | G-protein coupled receptor | 9.00 × 10^-4^ | *ADCY2, ADCY9,* ***AFF3****, CALML5, CAMK2A, CAMK2D,* ***CAMK2G****, CAMK4, CEP164, CEP70, EGFR, GNAQ, GRM5,* ***NCALD****, PIK3CG, PLA2G5, PLA2G6, PLCB1, PLK4, PPP2R1A, PPP3CA, PRKCA, PRKCB, PRKX, RAF1, RAP1A, RPS6KA2, RPS6KA3, YWHAG* |
| AKAP9 | other | 9.00 × 10^-4^ | *CAMK2A, GRIA1, GRIN2A, HSP90AA1, ITPR1, PLA2G5* |
| BDNF | growth factor | 1.00 × 10^-3^ | *CAMK2A, GNAQ, GRIA1, GRIN2B, ITPR1, PTEN, YWHAG* |
| BLID | other | 1.00 × 10^-3^ | *CAMK4, CDK1, CDK6, PTEN* |
| miR-17-3p (and other miRNAs w/seed CUGCAGU) | mature microRNA | 1.00 × 10^-3^ | *CAMK4, CDK1, CDK6, PTEN* |
| APOPT1 | other | 1.00 × 10^-3^ | *CAMK4, CDK1, CDK6, PTEN* |
| CYGB | transporter | 1.00 × 10^-3^ | *CAMK4, CDK1, CDK6, PTEN* |
| maslinic acid | chemical - endogenous non-mammalian | 1.00 × 10^-3^ | *CDK6, RPS6KA2, YWHAG* |
| IAP | group | 1.10 × 10^-3^ | *CAMK4, CDK1, CDK6, PTEN* |
| GRIN1 | ion channel | 1.10 × 10^-3^ | *CAMK2A, GRIN2A* |
| DRD4 | G-protein coupled receptor | 1.20 × 10^-3^ | *CAMK2A, EGFR, GRIA1, GRIN2A* |
| latrepirdine | chemical drug | 1.20 × 10^-3^ | *CAMK2A, GRIA1, GRIN2A, ITPR1, ITPR2* |
| emricasan | chemical drug | 1.30 × 10^-3^ | *CAMK4, CDK1, CDK6, PTEN* |
| MAGEA3/MAGEA6 | other | 1.30 × 10^-3^ | *CAMK4, CDK1, CDK6, PTEN* |
| CRYAA/CRYAA2 | other | 1.30 × 10^-3^ | *CAMK4, CDK1, CDK6, PTEN* |
| BOK | other | 1.30 × 10^-3^ | *ITPR1, ITPR2* |
| PLA2 | group | 1.40 × 10^-3^ | *EGFR, GRIA1, GRIN2A, GRIN2B, GRM1, GRM5, PLA2G5, PLA2G6, PRKCA, PRKCB, PTEN, RAF1* |
| clobetasol propionate | chemical drug | 1.40 × 10^-3^ | *CAMK4, GRIA1, GRIN2A, GRIN2B, GRM1, GRM5, PTEN, RAF1* |
| HOXA10 | transcription regulator | 1.40 × 10^-3^ | *CAMK2B, CAMK2D,* ***CAMK2G****, EGFR, GRIN2A, GRM1, HSP90AA1, YWHAG* |
| isoflurane | chemical drug | 1.40 × 10^-3^ | ***AFF3****, AKAP9, CALML5,* ***CAMK2G****, CAMK4, CDK1, CDK6, CEP70, EGFR, GRIA1, GRIN2A, GRM1,* ***NCALD****, PIK3CG, PLCB1, PPP3CC, PRKX, PTEN, RAF1, RPS6KA3, TUBGCP5, YWHAG* |
| caspase | group | 1.40 × 10^-3^ | *CDK1, CDK6, PTEN* |
| miR-29b-3p (and other miRNAs w/seed AGCACCA) | mature microRNA | 1.50 × 10^-3^ | ***AFF3****, CALML5, CAMK2A, CAMK2B, CAMK2D, CAMK4, CDK6, CEP164, E2F3, GNAQ, GRIA1, GRIN2A, GRIN2B, GRM1, GRM5, ITPR1, ITPR2, PDGFRB, PIK3CA, PLA2G6, PPP2R1A, PPP3CA, PRKCA, PTEN, RAP1A, RPS6KA2, RPS6KA3, YWHAG* |
| PKN1 | kinase | 1.60 × 10^-3^ | *AKAP9, CALML5, CAMK2A, CAMK2B,* ***CAMK2G****, CDK1, CDK6, CEP164, CEP70, EGFR, GNAQ, GRIN2B, HSP90AA1, PDGFRB, PIK3CG, PLA2G5, PLA2G6, PLCB1, PLK4, PPP2R1A, PPP3CA, PRKCA, PRKCB, PRKX, RAF1, RPS6KA2, RPS6KA3, YWHAG* |
| HTR2A | G-protein coupled receptor | 1.60 × 10^-3^ | *EGFR, GRM1* |
| incyclinide | chemical drug | 1.70 × 10^-3^ | *EGFR, GRIA1, GRIN2A, GRIN2B, PIK3CG, PPP2R1A* |
| mir-8 | microRNA | 1.80 × 10^-3^ | ***AFF3****, CAMK2A, CAMK2B, CAMK2D,* ***CAMK2G****, CDK1, CEP164, CEP70, E2F3, EGFR, GNAQ, GRM5, HSP90AA1, ITPR2, PDGFRB, PLA2G5, PLA2G6, PLCB1, PLK4, PPP2R1A, PPP3CA, PPP3CC, PRKCA, PRKCB, PRKX, PTEN, RAF1, RAP1A, RPS6KA2, RPS6KA3, YWHAG* |
| loperamide | chemical drug | 1.80 × 10^-3^ | *CAMK4, EGFR, ITPR1, ITPR2* |
| CD37 | other | 1.90 × 10^-3^ | *CAMK2A, CAMK2B, CAMK2D, CDK1, CEP164, CEP70, GNAQ, GRIN2A, GRIN2B, GRM1, GRM5, NUMA1, PDGFRB, PIK3CA, PIK3CG, PLA2G5, PLA2G6, PLCB1, PLK4, PPP2R1A, PPP3CA, PRKCA, PRKCB, PRKX, RAP1A, RPS6KA2, RPS6KA3* |
| CRNDE | other | 1.90 × 10^-3^ | *E2F3, EGFR, PDGFRB* |
| adaphostin | chemical drug | 1.90 × 10^-3^ | *CDK6, RAF1* |
| TARBP2 | other | 2.00 × 10^-3^ | ***AFF3****, CAMK2A, CAMK2B,* ***CAMK2G****, CAMK4, CDK1, CEP164, CEP70, E2F3, GNAQ, GRIA1, GRIN2A, GRIN2B, GRM1, GRM5, HSP90AA1, ITPR1, ITPR2, PLA2G5, PLCB1, PLK4, PPP2R1A, PPP3CA, PRKX, PTEN, RAF1, RPS6KA2* |
| PND-1186 | chemical drug | 2.10 × 10^-3^ | *AKAP9, CAMK2A, CAMK2B, CAMK2D, CDK1, CEP164, CEP70, E2F3, GNAQ, GRIN2B, GRM1, GRM5, NUMA1, PDGFRB, PIK3CA, PIK3CG, PLA2G5, PLCB1, PLK4, PPP2R1A, PPP3CA, PRKCA, PRKCB, PRKX, RAP1A, RPS6KA2, RPS6KA3* |
| SLIRP | other | 2.10 × 10^-3^ | *CAMK2A, CAMK2B, CDK6, CEP70, EGFR, GNAQ, GRIA1, GRIN2A, GRIN2B, GRM1, GRM5, HSP90AA1, PLCB1, PLK4, PPP2R1A, PRKCA, PRKX, RAF1* |
| PHB2 | transcription regulator | 2.20 × 10^-3^ | ***AFF3****, CAMK2B, CAMK2D, CAMK4, CDK1, CDK6, CEP164, CEP70, E2F3, EGFR, GNAQ, GRIA1, GRIN2A, GRIN2B, GRM1, GRM5, HSP90AA1, ITPR1, PIK3CG, PLA2G5, PLA2G6, PLCB1, PLK4, PPP2R1A, PPP3CA, PRKCA, PRKX, PTEN, RPS6KA2* |
| D-2-amino-5-phosphonovaleric acid | chemical reagent | 2.20 × 10^-3^ | *CAMK2A, CAMK2B, GRIN2A, GRIN2B, ITPR1* |
| ganetespib | chemical drug | 2.20 × 10^-3^ | *CDK1, EGFR* |
| PDYN | transporter | 2.20 × 10^-3^ | *GRM1, GRM5* |
| miR-142-3p (and other miRNAs w/seed GUAGUGU) | mature microRNA | 2.20 × 10^-3^ | *ADCY9, PRKCA* |
| CD247 | transmembrane receptor | 2.30 × 10^-3^ | *HSP90AA1, ITPR1, PLA2G5* |
| oxyquinoline | chemical toxicant | 2.40 × 10^-3^ | *CAMK2A, CAMK4, CDK1, GRIA1, GRIN2B, GRM1, HSP90AA1, PTEN* |
| mir-19 | microRNA | 2.50 × 10^-3^ | *CAMK2A, CAMK2B, CAMK2D, CDK1, CDK6, CEP164, CEP70, E2F3, EGFR, GNAQ, GRIA1, GRIN2A, GRIN2B, HSP90AA1, PDGFRB, PIK3CA, PIK3CG, PLA2G5, PLA2G6, PLCB1, PLK4, PPP2R1A, PRKCB, PRKX, PTEN, RAP1A, RPS6KA2, RPS6KA3* |
| NHLRC1 | enzyme | 2.50 × 10^-3^ | *CAMK4, CDK1, EGFR, GRIA1, PRKCA, PTEN, RAF1* |
| GRIN2C | ion channel | 2.50 × 10^-3^ | *CAMK2A, CAMK2D, GRIN2A, ITPR1* |
| COMT | enzyme | 2.50 × 10^-3^ | *CAMK2A, CAMK4* |
| Esr1-Estrogen-Sp1 | complex | 2.60 × 10^-3^ | ***AFF3****, CAMK2B, CAMK2D,* ***CAMK2G****, CAMK4, CDK1, CDK6, CEP70, EGFR, GNAQ, GRIA1, GRIN2A, GRIN2B, GRM1, GRM5, HSP90AA1, ITPR1, PDGFRB, PIK3CG, PLA2G5, PLA2G6, PLCB1, PLK4, PPP2R1A, PPP3CA, PRKCA, PRKX, PTEN, RAF1* |
| brexpiprazole | chemical drug | 2.60 × 10^-3^ | *ADCY2, ADCY9, CALML5, CAMK2A, CAMK4, CDK6, EGFR, GRIN2A, GRM1, GRM5, ITPR1, PIK3CA, PIK3CG, PLA2G5, PLCB1, PPP2R1A, PRKCA, TUBGCP5* |
| phenylephrine | chemical drug | 2.60 × 10^-3^ | *E2F3, EGFR, ITPR2, PRKCB* |
| DRD4 | G-protein coupled receptor | 2.70 × 10^-3^ | *ADCY2, ADCY9, CAMK2A, CAMK2B, CAMK2D, CEP164, EGFR, GRIA1, GRIN2A, GRM5, HSP90AA1, ITPR1, ITPR2, PDGFRB, PIK3CG, PLA2G5, PLA2G6, PPP2R1A, PPP3CA, PRKCA, RPS6KA2, YWHAG* |
| CD44 | other | 2.70 × 10^-3^ | *ADCY2, ADCY9,* ***AFF3****, AKAP9, CAMK2B,* ***CAMK2G****, CAMK4, CEP70, E2F3, EGFR,* ***GAB3****, GNAQ, GRIA1, GRIN2A, GRIN2B, HSP90AA1, ITPR1, ITPR2,* ***NCALD****, PIK3CA, PLA2G5, PLCB1, PLK4, PPP2R1A, PPP3CA, PPP3CC, PRKCA, PRKX, RAP1A, RPS6KA3, TUBGCP5* |
| NR3C1 | ligand-dependent nuclear receptor | 2.70 × 10^-3^ | *GRIA1, GRIN2A, GRIN2B, GRM1, GRM5, PTEN, RAF1* |
| fluoxymesterone | chemical drug | 2.80 × 10^-3^ | ***AFF3****, CAMK2B, CAMK2D, CAMK4, CDK1, CDK6, CEP164, CEP70, E2F3, EGFR, GNAQ, GRIA1, GRIN2A, GRIN2B, GRM1, GRM5, HSP90AA1, ITPR1, ITPR2, PIK3CG, PLA2G6, PLCB1, PLK4, PPP2R1A, PPP3CA, PRKX, PTEN, RPS6KA2, YWHAG* |
| DENND3 | other | 2.80 × 10^-3^ | ***AFF3****, AKAP9, CAMK2A, CAMK2B, CAMK2D, CAMK4, CEP164, CEP70, E2F3, GNAQ, GRIA1, GRIN2A, GRM1, GRM5, ITPR2, PDGFRB, PIK3CG, PLA2G5, PLA2G6, PLCB1, PLK4, PPP2R1A, PPP3CA, PRKCB, PRKX, RAF1, RPS6KA2, RPS6KA3* |
| ML-7 | chemical - kinase inhibitor | 2.80 × 10^-3^ | *EGFR, ITPR1, ITPR2* |
| HSPA8 | enzyme | 2.90 × 10^-3^ | ***AFF3****, CALML5, CAMK2B, CAMK2D, CDK1, CEP164, CEP70, GNAQ, GRM1, GRM5, HSP90AA1, ITPR1, ITPR2, PIK3CG, PLA2G5, PLA2G6, PLCB1, PLK4, PPP2R1A, PPP3CA, PRKCA, PRKX, RAF1, RAP1A, RPS6KA2, RPS6KA3, TUBGCP5* |
| PHLPP2 | enzyme | 2.90 × 10^-3^ | *PRKCA, PRKCB* |
| PSMD10 | transcription regulator | 2.90 × 10^-3^ | *PIK3CA, PTEN* |
| PELP1 | other | 3.00 × 10^-3^ | ***AFF3****, CAMK2B, CAMK2D, CAMK4, CDK1, CDK6, CEP164, CEP70, E2F3, EGFR, GNAQ, GRIA1, GRIN2A, GRIN2B, GRM1, GRM5, HSP90AA1, ITPR1, PIK3CG, PLA2G5, PLA2G6, PLCB1, PLK4, PPP2R1A, PPP3CA, PRKCA, PRKX, PTEN, RPS6KA2* |
| dieldrin | chemical toxicant | 3.00 × 10^-3^ | *CAMK2A, CAMK2B, CDK1, CDK6, CEP70, GNAQ, GRIA1, GRIN2A, HSP90AA1, PLCB1, PLK4, PPP2R1A, PRKX, PTEN* |
| NCR2 | transmembrane receptor | 3.00 × 10^-3^ | *CDK1, EGFR, GRIA1, HSP90AA1, ITPR1, PIK3CA* |
| HTT | transcription regulator | 3.10 × 10^-3^ | *CAMK2A, CAMK4, GRIN2A, GRIN2B, GRM1* |
| 8, 9-epoxyeicosatrienoic acid | chemical - endogenous mammalian | 3.10 × 10^-3^ | *EGFR, PIK3CG* |
| NYAP1 | other | 3.20 × 10^-3^ | ***AFF3****, CALML5, CAMK2D,* ***CAMK2G****, CAMK4, CEP164, CEP70, EGFR, GNAQ, GRM5,* ***NCALD****, PDGFRB, PIK3CA, PIK3CG, PLA2G5, PLA2G6, PLCB1, PLK4, PPP2R1A, PPP3CA, PRKCB, PRKX, PTEN, RAF1, RAP1A, RPS6KA2, RPS6KA3, YWHAG* |
| MYO16 | other | 3.20 × 10^-3^ | ***AFF3****, CALML5, CAMK2D,* ***CAMK2G****, CAMK4, CEP164, CEP70, EGFR, GNAQ, GRM5,* ***NCALD****, PDGFRB, PIK3CA, PIK3CG, PLA2G5, PLA2G6, PLCB1, PLK4, PPP2R1A, PPP3CA, PRKCB, PRKX, PTEN, RAF1, RAP1A, RPS6KA2, RPS6KA3, YWHAG* |
| NYAP2 | other | 3.20 × 10^-3^ | ***AFF3****, CALML5, CAMK2D,* ***CAMK2G****, CAMK4, CEP164, CEP70, EGFR, GNAQ, GRM5,* ***NCALD****, PDGFRB, PIK3CA, PIK3CG, PLA2G5, PLA2G6, PLCB1, PLK4, PPP2R1A, PPP3CA, PRKCB, PRKX, PTEN, RAF1, RAP1A, RPS6KA2, RPS6KA3, YWHAG* |
| ZGPAT | transcription regulator | 3.20 × 10^-3^ | *EGFR, PTEN* |
| hormone | chemical drug | 3.30 × 10^-3^ | *CAMK2B, CDK6, CEP70, EGFR, GNAQ, GRIA1, GRIN2A, GRIN2B, GRM1, GRM5, HSP90AA1, PLCB1, PLK4, PPP2R1A, PRKX, PTEN, RAF1* |
| SESN2 | enzyme | 3.30 × 10^-3^ | ***AFF3****, CALML5,* ***CAMK2G****, CAMK4, CDK1, CEP70, EGFR, GRIA1, GRM5, HSP90AA1, ITPR2, PIK3CA, PIK3CG, PLA2G5, PLA2G6, PLK4, PPP2R1A, PRKCA, PRKCB, PRKX, PTEN, RAF1, RAP1A, RPS6KA3, YWHAG* |
| MDL 28170 | chemical toxicant | 3.30 × 10^-3^ | *GRIA1, GRM1* |
| miR-486-5p (and other miRNAs w/seed CCUGUAC) | mature microRNA | 3.30 × 10^-3^ | ***AFF3****, PTEN* |
| RPS6KA1 | kinase | 3.50 × 10^-3^ | *ADCY2, ADCY9, AKAP9, CALML5, CAMK2A, CAMK2D,* ***CAMK2G****, CAMK4, CEP70, E2F3, EGFR, GNAQ, GRIN2A, GRM1, GRM5, HSP90AA1, ITPR1,* ***NCALD****, PDGFRB, PIK3CA, PLA2G6, PLCB1, PLK4, PPP2R1A, PPP3CC, PRKCA, PRKX, RPS6KA3, TUBGCP5, YWHAG* |
| SH3GLB1 | enzyme | 3.50 × 10^-3^ | ***AFF3****, CALML5, CAMK2A, CAMK2B, CAMK2D, CAMK4, CDK1, CDK6, CEP70, GNAQ, GRIN2A, GRIN2B, GRM1, GRM5, ITPR2, PIK3CG, PLA2G5, PLA2G6, PLCB1, PLK4, PPP2R1A, PPP3CA, PRKCB, PRKX, PTEN, RAP1A, RPS6KA3* |
| mometasone furoate | chemical drug | 3.70 × 10^-3^ | *CDK1, EGFR, GRIA1, GRIN2A, GRIN2B, GRM1, GRM5, PTEN, RAF1* |
| mir-145 | microRNA | 3.70 × 10^-3^ | *CDK6, E2F3, EGFR* |
| BLOC1S5 | other | 3.70 × 10^-3^ | *GRIN2A, GRIN2B* |
| phentolamine | chemical drug | 3.80 × 10^-3^ | *CALML5, CAMK2A, CAMK2B, CAMK4, CDK6, CEP70, GNAQ, GRIA1, GRM5, HSP90AA1, ITPR1, PDGFRB, PIK3CA, PIK3CG, PLA2G5, PLCB1, PLK4, PPP2R1A, PPP3CA, PPP3CC, PRKCB, PRKX, RAF1, TUBGCP5* |
| alisertib | chemical drug | 3.80 × 10^-3^ | *NUMA1* |
| UBXN2B | other | 3.80 × 10^-3^ | *NUMA1* |
| AIMP2 | other | 4.00 × 10^-3^ | *CAMK2A, CAMK2B, CAMK2D, CAMK4, CDK1, CEP164, E2F3, EGFR, GRIA1, GRIN2A, GRIN2B, GRM1, GRM5, HSP90AA1, ITPR1, PDGFRB, PIK3CA, PIK3CG, PLA2G5, PPP3CA, PRKCA, PTEN, RAF1, RPS6KA2* |
| 1, 10-phenanthroline | chemical toxicant | 4.00 × 10^-3^ | ***AFF3****, CAMK2A, CAMK2B, CAMK2D,* ***CAMK2G****, CDK1, CEP164, CEP70,* ***GAB3****, GNAQ, GRIA1, GRM5, HSP90AA1, ITPR2, NUMA1, PDGFRB, PLA2G6, PLCB1, PLK4, PPP2R1A, PPP3CA, PRKCA, PRKX, PTEN, RAF1, RAP1A, RPS6KA2, RPS6KA3, YWHAG* |
| miconazole | chemical drug | 4.00 × 10^-3^ | *EGFR, GRIA1, GRIN2A, GRIN2B, GRM1, GRM5, PDGFRB, PTEN, RAF1* |
| ASCL1 | transcription regulator | 4.00 × 10^-3^ | *CDK1, CDK6, EGFR, GRM5, PTEN* |
| CDC37 | kinase | 4.00 × 10^-3^ | *CDK6, RAF1* |
| RGS19 | other | 4.10 × 10^-3^ | *ADCY2, ADCY9,* ***AFF3****, CAMK2A, CAMK2D,* ***CAMK2G****, CEP164, CEP70, EGFR, GNAQ,* ***NCALD****, PDGFRB, PIK3CG, PLA2G5, PLK4, PPP2R1A, PPP3CA, PRKCA, PRKCB, PRKX, RAF1, RAP1A, RPS6KA2, RPS6KA3, YWHAG* |
| fluocinolone acetonide | chemical drug | 4.10 × 10^-3^ | *GRIA1, GRIN2A, GRIN2B, GRM1, GRM5, PRKCB, PTEN, RAF1* |
| drospirenone | chemical drug | 4.30 × 10^-3^ | *CAMK2A, CAMK2B, CDK6, CEP70, EGFR, GNAQ, GRIA1, GRIN2A, GRIN2B, GRM1, GRM5, HSP90AA1, PLCB1, PLK4, PPP2R1A, PRKCA, PRKX, RAF1* |
| FGF3 | growth factor | 4.40 × 10^-3^ | *ADCY2, ADCY9,* ***AFF3****, CAMK2A, CAMK2B, CAMK2D,* ***CAMK2G****, CDK1, CDK6, CEP164, CEP70, EGFR,* ***GAB3****, GNAQ, GRM5, HSP90AA1, ITPR2, PDGFRB, PLA2G5, PLA2G6, PLCB1, PLK4, PPP3CA, PRKCA, PRKCB, PRKX, PTEN, RAF1, RPS6KA2, RPS6KA3, YWHAG* |
| mir-486 | microRNA | 4.50 × 10^-3^ | ***AFF3****, PTEN* |
| CTHRC1 | other | 4.60 × 10^-3^ | *AKAP9, CAMK2B, CAMK2D, CAMK4, CDK1, CDK6, CEP164, CEP70, GNAQ, GRIN2A, GRIN2B, ITPR1, ITPR2, PDGFRB, PIK3CA, PIK3CG, PLA2G5, PLA2G6, PLCB1, PLK4, PPP2R1A, PPP3CA, PRKCA, PRKX, RPS6KA2* |
| trichloroethylene | chemical toxicant | 4.60 × 10^-3^ | ***AFF3****, CAMK2A, CAMK2B, CDK6, CEP70, E2F3, GNAQ, GRIN2A, GRIN2B, GRM1, GRM5, ITPR2, NUMA1, PIK3CA, PIK3CG, PLA2G5, PLCB1, PLK4, PPP2R1A, PRKX, PTEN, RAP1A, RPS6KA3* |
| SLC30A3 | transporter | 4.60 × 10^-3^ | *GRIN2A, GRIN2B* |
| deoxycorticosterone acetate | chemical drug | 4.70 × 10^-3^ | ***AFF3****, CALML5, CAMK2A, CAMK2D, CAMK4, E2F3, EGFR, GNAQ, GRM5, HSP90AA1, ITPR2, PDGFRB, PIK3CA, PPP2R1A, PPP3CA, PRKCA, PTEN, RAP1A, RPS6KA3, YWHAG* |
| cytochrome C | group | 4.80 × 10^-3^ | *CAMK4, CDK1, CDK6, ITPR1, PTEN* |
| JARID2 | transcription regulator | 4.80 × 10^-3^ | *CDK1, PTEN* |
| tubulin (complex) | complex | 5.00 × 10^-3^ | ***AFF3****, CAMK2A, CAMK2D, CAMK4, CDK6, CEP164, CEP70, EGFR, GNAQ, GRIN2A, GRIN2B, GRM1, GRM5, ITPR2, PIK3CG, PLA2G6, PLCB1, PLK4, PPP2R1A, PPP3CA, PPP3CC, PRKX, RPS6KA2, RPS6KA3* |
| cation | chemical - other | 5.00 × 10^-3^ | *EGFR, ITPR1, ITPR2, PPP2R1A* |
| Esr1-Estrogen-Sp1 | complex | 5.20 × 10^-3^ | *CAMK2B, CDK1, CDK6, CEP70, EGFR, GNAQ, GRM1, HSP90AA1, ITPR1, PDGFRB, PLCB1, PLK4, PPP2R1A, PRKX, PTEN, RAF1* |
| Epha dimer | complex | 5.40 × 10^-3^ | ***AFF3****, CALML5, CAMK2A, CAMK2B, CAMK2D, CEP164, CEP70, GNAQ, GRIN2A, GRIN2B, GRM1, GRM5, PIK3CG, PLA2G5, PLCB1, PLK4, PPP2R1A, PRKCB, PRKX, RAF1, RAP1A, RPS6KA2, RPS6KA3, YWHAG* |
| buprenorphine | chemical drug | 5.60 × 10^-3^ | *ADCY2, ADCY9,* ***AFF3****, CAMK2A, CAMK4, EGFR, GNAQ, GRIN2A, GRIN2B, HSP90AA1, PDGFRB, PIK3CG, PLCB1, PPP3CA, PRKCB, RAP1A, RPS6KA3* |
| DDC | enzyme | 5.90 × 10^-3^ | *ADCY2, ADCY9, CAMK2B, CAMK2D, CDK6, CEP164, CEP70, EGFR, GNAQ, GRM1, GRM5, ITPR2, PIK3CA, PIK3CG, PLA2G5, PLA2G6, PLCB1, PLK4, PPP2R1A, PPP3CA, PRKCA, PRKCB, PRKX, PTEN, RAF1, RPS6KA2* |
| terfenadine | chemical drug | 5.90 × 10^-3^ | ***AFF3****, CAMK2A, CAMK4, CDK1, EGFR, GNAQ, GRIA1, GRIN2A, GRIN2B, HSP90AA1, ITPR2, PDGFRB, PIK3CG, PLCB1, PRKCB, RAF1, RAP1A, RPS6KA3* |
| barbiturate | chemical drug | 5.90 × 10^-3^ | *EGFR, GNAQ, GRIN2B, HSP90AA1, ITPR1, PRKCA, RAP1A* |
| voltage-gated calcium channel | complex | 5.90 × 10^-3^ | *ITPR1, ITPR2* |
| 4-nitroquinoline-1-oxide | chemical toxicant | 5.90 × 10^-3^ | *CDK1, CDK6* |
| PAIP2 | translation regulator | 5.90 × 10^-3^ | *CAMK2A* |
| Crk-DOCK 180-Paxillin | complex | 6.00 × 10^-3^ | *CAMK2B, CAMK2D, CAMK4, CDK1, CDK6, CEP164, CEP70, GNAQ, GRIN2A, GRIN2B, ITPR1, ITPR2, PDGFRB, PIK3CG, PLA2G5, PLA2G6, PLCB1, PLK4, PPP2R1A, PPP3CA, PRKCA, PRKX, RAP1A, RPS6KA2* |
| aripiprazole | chemical drug | 6.00 × 10^-3^ | *ADCY2, ADCY9, CAMK2A, CAMK4, CDK6, EGFR, GRIN2A, GRM1, GRM5, ITPR1, NUMA1, PIK3CA, PIK3CG, PLA2G5, PLA2G6, PLCB1, TUBGCP5* |
| CREBBP | transcription regulator | 6.00 × 10^-3^ | *CAMK2B, CAMK2D,* ***CAMK2G****, GRIN2A, GRM1* |
| BID | other | 6.10 × 10^-3^ | ***AFF3****,* ***CAMK2G****, CAMK4, CDK1, CDK6, CEP164, CEP70, E2F3, EGFR, GNAQ, ITPR1, ITPR2,* ***NCALD****, PLA2G5, PLA2G6, PLCB1, PLK4, PPP3CA, PRKCB, PRKX, PTEN, RAF1, RAP1A, RPS6KA2, RPS6KA3* |
| CBP-ICSBP-IRF-1-PU.1 | complex | 6.20 × 10^-3^ | *CALML5, CAMK2A, CAMK2B, CAMK2D,* ***CAMK2G****, CAMK4, CDK1, CDK6, CEP164, CEP70, E2F3, GNAQ, GRIN2A, GRIN2B, GRM1, GRM5, PIK3CG, PLCB1, PLK4, PPP2R1A, PPP3CA, PRKCB, PRKX, RAF1, RPS6KA2, RPS6KA3* |
| alisertib | chemical drug | 6.20 × 10^-3^ | *NUMA1, PTEN* |
| GRASP | other | 6.20 × 10^-3^ | *GRM1* |
| OPHN1 | other | 6.20 × 10^-3^ | *GRM1* |
| SH3GL1 | other | 6.20 × 10^-3^ | *GRM1* |
| DNAJB6 | transcription regulator | 6.30 × 10^-3^ | ***AFF3****, AKAP9, CAMK2A, CAMK2B, CAMK2D, CAMK4, CDK1, CDK6, CEP164, CEP70, E2F3, GNAQ, GRIN2A, GRM1, GRM5, ITPR2, PDGFRB, PIK3CG, PLCB1, PLK4, PPP2R1A, PPP3CA, PRKX, PTEN, RAF1, RPS6KA2, RPS6KA3* |
| (2-(2', 3'-dihydrobenzo(1, 4)dioxin-6'-yl)-2-hydroxy-1-pyrrolidin-1-ylmethylethyl)nonanoic acid amide | chemical reagent | 6.40 × 10^-3^ | *CAMK2A, CAMK2B, CAMK2D, CDK1, CEP70, E2F3, GNAQ, GRM1, GRM5, HSP90AA1, ITPR1, NUMA1, PDGFRB, PIK3CA, PIK3CG, PLA2G5, PLCB1, PLK4, PPP2R1A, PRKCB, PRKX, RAP1A, RPS6KA3* |
| CEACAM6 | other | 6.40 × 10^-3^ | *CAMK4, CDK1, EGFR, PDGFRB, PTEN* |
| CLCN3 | ion channel | 6.40 × 10^-3^ | *CAMK4, EGFR, PTEN* |
| SET complex | complex | 6.60 × 10^-3^ | *CAMK2D,* ***CAMK2G****, CAMK4, CDK1, CDK6, CEP164, CEP70, E2F3, GNAQ, GRIA1, GRIN2A, GRIN2B, GRM1, PDGFRB, PIK3CG, PLA2G5, PLCB1, PLK4, PPP2R1A, PPP3CA, PRKCA, PRKCB, PRKX, RPS6KA2* |
| CEACAM6 | other | 6.60 × 10^-3^ | *AKAP9, CALML5, CAMK2A, CAMK2B, CAMK4, CDK1, CEP164, CEP70, E2F3, EGFR, GRIN2A, GRIN2B, GRM1, GRM5, ITPR2, PDGFRB, PLA2G5, PLCB1, PLK4, PPP2R1A, PPP3CA, PRKX, PTEN, RAP1A, RPS6KA2, YWHAG* |
| romidepsin | biologic drug | 6.70 × 10^-3^ | *EGFR, RAF1, RAP1A* |
| poly(ADP-ribose) | chemical - endogenous mammalian | 6.80 × 10^-3^ | *CAMK2B, CDK1, CDK6, CEP70, E2F3, EGFR, GNAQ, GRIA1, HSP90AA1, ITPR1, ITPR2, PLCB1, PLK4, PPP2R1A, PRKX, PTEN* |
| PRKG2 | kinase | 6.90 × 10^-3^ | ***AFF3****, CALML5, CAMK2A, CAMK4, CDK1, CEP70, EGFR, GNAQ, GRM1, ITPR2, PIK3CA, PLA2G6, PLCB1, PLK4, PPP2R1A, PRKCA, PRKX, PTEN, RAP1A, RPS6KA3, YWHAG* |
| 5'-guanylylimidodiphosphate | chemical reagent | 6.90 × 10^-3^ | *EGFR, HSP90AA1, ITPR1, ITPR2, PTEN* |
| CDC6 | other | 6.90 × 10^-3^ | *CEP192* |
| CCNQ | other | 7.00 × 10^-3^ | *RAF1* |
| PLCB4 | enzyme | 7.00 × 10^-3^ | *PLCB1* |
| SKI | transcription regulator | 7.10 × 10^-3^ | ***AFF3****, CAMK2A, CAMK2B, CAMK2D, CDK1, CDK6, CEP164, CEP192, CEP70, EGFR, GNAQ, GRM5, HSP90AA1,* ***NCALD****, NUMA1, PDGFRB, PIK3CA, PLA2G5, PLA2G6, PLCB1, PLK4, PPP2R1A, PPP3CA, PRKCA, PRKX, PTEN, RAP1A, RPS6KA2, RPS6KA3* |
| KIR3DL1 | transmembrane receptor | 7.10 × 10^-3^ | ***AFF3****, CAMK2A, CAMK2D,* ***CAMK2G****, CEP164, CEP70, EGFR, GNAQ, GRM5, ITPR2,* ***NCALD****, PIK3CG, PLA2G6, PLCB1, PLK4, PPP2R1A, PPP3CA, PRKCB, PRKX, PTEN, RAF1, RAP1A, RPS6KA2, RPS6KA3* |
| COMT | enzyme | 7.10 × 10^-3^ | *CAMK2A, CAMK4, CDK1, GRIA1, GRIN2B, GRM1, HSP90AA1* |
| U73122 | chemical reagent | 7.10 × 10^-3^ | *E2F3, ITPR2, PLCB1* |
| DLGAP3 | other | 7.10 × 10^-3^ | *GRM5* |
| RIC8A | other | 7.20 × 10^-3^ | *GNAQ* |
| tubulin (complex) | complex | 7.20 × 10^-3^ | *GNAQ* |
| STRADA | kinase | 7.30 × 10^-3^ | *AKAP9, CAMK2D, CAMK4, CEP164, CEP70, E2F3, GNAQ, GRIA1, GRIN2A, GRIN2B, GRM5, ITPR2,* ***NCALD****, PDGFRB, PIK3CG, PLA2G5, PLA2G6, PLCB1, PLK4, PPP2R1A, PPP3CA, PRKX, PTEN, RAF1, RPS6KA2* |
| methysergide | chemical drug | 7.30 × 10^-3^ | *CALML5, CAMK2A, CAMK4, CDK1, E2F3, EGFR, GRIA1, GRIN2A, GRM1, PIK3CA, PRKCA, PTEN, YWHAG* |
| PPP3CA | phosphatase | 7.30 × 10^-3^ | ***AFF3****, CAMK2A, CAMK2D,* ***CAMK2G****, CAMK4, CEP164, E2F3, GNAQ, GRIA1, GRIN2A, GRIN2B, GRM1, GRM5, ITPR1, PIK3CA, PLA2G5, PLA2G6, PRKCB, PTEN, RAF1, RPS6KA2, RPS6KA3, YWHAG* |
| SIN3A | transcription regulator | 7.30 × 10^-3^ | *CDK1, CDK6, GNAQ, GRIN2B, GRM1, HSP90AA1, ITPR1, PDGFRB, PRKCA, PTEN, RAP1A* |
| trans-hydroxytamoxifen | chemical drug | 7.40 × 10^-3^ | *ADCY9, CAMK2B, CDK1, CDK6, CEP70, EGFR, GNAQ, HSP90AA1, PLCB1, PLK4, PPP2R1A, PRKX, PTEN* |
| 26s Proteasome | complex | 7.40 × 10^-3^ | *CDK1, EGFR, GRIA1, PRKCA, PTEN* |
| ADAM10 | peptidase | 7.40 × 10^-3^ | *GRIN2A, GRIN2B* |
| TP53BP2 | other | 7.70 × 10^-3^ | *ADCY2, ADCY9,* ***AFF3****, CALML5, CAMK2A, CEP70, E2F3, EGFR, GNAQ, GRIN2A, GRIN2B, GRM1, GRM5, ITPR1, ITPR2, PIK3CA, PLA2G5, PLA2G6, PLCB1, PLK4, PPP2R1A, PPP3CA, PRKCB, PRKX, PTEN, RAF1, RAP1A, RPS6KA3* |
| 1, 2-dithiol-3-thione | chemical reagent | 7.80 × 10^-3^ | *ADCY2,* ***CAMK2G****, CDK1, CEP70, EGFR, GNAQ, GRIA1, GRM1, GRM5, HSP90AA1, ITPR2, PLA2G5, PLCB1, PLK4, PPP2R1A, PRKCA, PRKCB, PRKX, PTEN, RAF1, RAP1A* |
| miR-103-3p (and other miRNAs w/seed GCAGCAU) | mature microRNA | 7.80 × 10^-3^ | *CDK6, PIK3CG* |
| OPRL1 | G-protein coupled receptor | 7.80 × 10^-3^ | *PPP3CA* |
| GFRA4 | transmembrane receptor | 7.90 × 10^-3^ | ***AFF3****, AKAP9, CALML5, CAMK2A, CAMK2D,* ***CAMK2G****, CAMK4, CEP164, CEP70, EGFR, GNAQ, GRM5, HSP90AA1,* ***NCALD****, PIK3CG, PLA2G5, PLA2G6, PLCB1, PLK4, PPP2R1A, PPP3CA, PRKCB, PRKX, RAF1, RAP1A, RPS6KA2, RPS6KA3* |
| nitroaspirin | chemical drug | 7.90 × 10^-3^ | *CAMK4, GRIA1, GRIN2A, GRIN2B, PIK3CG, PTEN* |
| DNMT3L | transcription regulator | 7.90 × 10^-3^ | *CAMK2A, CAMK4, CDK1, ITPR2, PRKCB* |
| Thrombospondin | group | 8.00 × 10^-3^ | ***AFF3****, CALML5, CAMK2A, CAMK2B, CAMK2D, CDK1, CDK6, CEP164, EGFR, GRIA1, GRIN2A, GRIN2B, GRM1, GRM5, HSP90AA1, ITPR1, ITPR2, PDGFRB, PIK3CA, PIK3CG, PLA2G5, PLCB1, PLK4, PPP3CC, PTEN, RAP1A, RPS6KA2, RPS6KA3, TUBGCP5* |
| CLSTN1 | other | 8.00 × 10^-3^ | *GRIN2B* |
| KIF17 | transporter | 8.00 × 10^-3^ | *GRIN2B* |
| chlorpyrifos | chemical toxicant | 8.10 × 10^-3^ | *ADCY2,* ***AFF3****, CALML5, CAMK2A, CAMK4, CDK6, CEP70, E2F3, GNAQ, GRIA1, GRIN2A, GRM1, GRM5, PIK3CA, PLA2G6, PLCB1, PLK4, PPP2R1A, PPP3CC, PRKCB, PRKX, RAF1, RPS6KA3* |
| latrepirdine | chemical drug | 8.20 × 10^-3^ | *ADCY2, ADCY9, CALML5, CAMK2A, CAMK2D, CDK6, E2F3, GRIA1, GRIN2A, GRIN2B, GRM5, ITPR1, ITPR2, PLA2G5, PLA2G6, PLCB1, PPP2R1A, TUBGCP5, YWHAG* |
| picropodophyllin | chemical drug | 8.20 × 10^-3^ | *CDK1, CDK6* |
| CBP-ICSBP-IRF-1-PU.1 | complex | 8.30 × 10^-3^ | *CAMK2B, CAMK2D,* ***CAMK2G****, CDK1, CDK6, E2F3, GRIN2A, GRM1, PIK3CG* |
| MECP2 | transcription regulator | 8.30 × 10^-3^ | *CAMK2A, CAMK2B, CDK1, CDK6, GRIA1, GRIN2A, PIK3CG* |
| NT5C2 | phosphatase | 8.40 × 10^-3^ | ***AFF3****, CALML5, CAMK2A, CAMK2B, CAMK2D, CAMK4, CEP164, E2F3, GNAQ, GRIA1, GRIN2A, GRIN2B, GRM1, ITPR2, NUMA1, PIK3CA, PIK3CG, PLA2G5, PLA2G6, PLCB1, RAF1, RPS6KA2, RPS6KA3, YWHAG* |
| RPS6KA5 | kinase | 8.40 × 10^-3^ | ***AFF3****, CALML5, CAMK2A, CAMK2B, CAMK2D,* ***CAMK2G****, CAMK4, CEP70, E2F3, GNAQ, GRIN2A, GRM1, GRM5, HSP90AA1, ITPR1, ITPR2, MNAT1, NUMA1, PDGFRB, PIK3CA, PLA2G6, PLCB1, PLK4, PPP2R1A, PRKCA, PRKX, PTEN, RPS6KA3, TUBGCP5* |
| Corticosteroid-GCR | complex | 8.50 × 10^-3^ | *ADCY9,* ***AFF3****, CALML5, CAMK2A, CAMK2B,* ***CAMK2G****, CAMK4, CDK1, CDK6, CEP164, E2F3, GNAQ, GRIA1, GRIN2A, GRIN2B, GRM1, GRM5, HSP90AA1, ITPR1, PLA2G5, PPP3CA, PTEN, RAF1, RPS6KA2* |
| pazopanib | chemical drug | 8.60 × 10^-3^ | *AKAP9, CAMK2A,* ***CAMK2G****, CDK1, CEP164, CEP70, E2F3, EGFR,* ***GAB3****, GNAQ, GRIN2A, GRM1, GRM5, HSP90AA1, NUMA1, PIK3CA, PIK3CG, PLA2G5, PLA2G6, PLCB1, PLK4, PRKCB, PRKX, PTEN, RAP1A, RPS6KA2, RPS6KA3* |
| DNMT3B | enzyme | 8.70 × 10^-3^ | *CAMK2A, CAMK4, CDK1, ITPR2* |
| thioproperazine | chemical drug | 8.90 × 10^-3^ | *ADCY2, ADCY9, CAMK2A, CAMK4, CDK6, E2F3, EGFR, GRIN2A, GRM1, GRM5, ITPR1, PDGFRB, PLA2G5, PLCB1, PPP2R1A, PRKCA, PTEN, TUBGCP5* |
| tryptamine | chemical - endogenous mammalian | 8.90 × 10^-3^ | *CAMK2A, EGFR, GNAQ, GRIA1, GRIN2A, GRM1, HSP90AA1, ITPR1, PRKCA, PRKCB, PTEN, RAP1A* |
| SCNN1A | ion channel | 8.90 × 10^-3^ | *ITPR1, PIK3CA, PLA2G5, PTEN* |
| SGK1 | kinase | 8.90 × 10^-3^ | *CDK6, GRIN2A, GRIN2B* |
| miR-7a-5p (and other miRNAs w/seed GGAAGAC) | mature microRNA | 8.90 × 10^-3^ | *EGFR, RAF1* |
| miR-211-3p (miRNAs w/seed CAGGGAC) | mature microRNA | 8.90 × 10^-3^ | *CDK6* |
| SCNN1B | ion channel | 9.00 × 10^-3^ | *ITPR1, PIK3CA, PLA2G5, PTEN* |
| Cyclin B | group | 9.10 × 10^-3^ | ***AFF3****, CAMK2B, CAMK2D, CDK6, CEP164, CEP192, CEP70, GNAQ, GRIN2A, GRIN2B, HSP90AA1, ITPR2, NUMA1, PDGFRB, PIK3CA, PLA2G5, PLCB1, PLK4, PPP2R1A, PPP3CA, PRKCA, PRKCB, PRKX, PTEN, RAF1, RAP1A, RPS6KA2, RPS6KA3* |
| ellagic acid | chemical - endogenous non-mammalian | 9.10 × 10^-3^ | *ADCY2, ADCY9,* ***AFF3****, AKAP9, CALML5,* ***CAMK2G****, CDK1, CDK6, CEP70, E2F3, GNAQ, GRIA1, GRIN2A, GRIN2B, GRM5, ITPR1, NUMA1, PIK3CG, PLA2G5, PLA2G6, PLCB1, PLK4, PPP2R1A, PPP3CC, PRKCB, PRKX, PTEN, RPS6KA3, YWHAG* |
| SLV-313 | chemical drug | 9.20 × 10^-3^ | *ADCY2, ADCY9, CAMK2A, CAMK4, E2F3, GRIA1, GRIN2A, GRM5, ITPR1, PDGFRB, PIK3CG, PPP2R1A, PRKCA, PTEN, YWHAG* |
| ESR1 | ligand-dependent nuclear receptor | 9.20 × 10^-3^ | *CAMK2B, CDK1, CDK6, CEP70, EGFR, GNAQ, HSP90AA1, PLCB1, PLK4, PPP2R1A, PRKX, PTEN* |
| rhesus theta-defensin 2 | chemical - endogenous mammalian | 9.20 × 10^-3^ | *EGFR, GRIN2A, GRIN2B* |
| rhesus theta-defensin 3 | chemical - endogenous mammalian | 9.20 × 10^-3^ | *EGFR, GRIN2A, GRIN2B* |
| miR-34a-5p (and other miRNAs w/seed GGCAGUG) | mature microRNA | 9.20 × 10^-3^ | *CDK1, CDK6, E2F3* |
| CDKN3 | phosphatase | 9.20 × 10^-3^ | *CDK1* |
| GRID2 | ion channel | 9.20 × 10^-3^ | *CDK1* |
| terameprocol | chemical drug | 9.20 × 10^-3^ | *CDK1* |
| rhesus theta-defensin 1 | chemical - endogenous mammalian | 9.40 × 10^-3^ | *EGFR, GRIN2A, GRIN2B* |
| leukotriene C4 | chemical - endogenous mammalian | 9.50 × 10^-3^ | *CAMK2B, CAMK2D, CAMK4, CDK6, CEP164, CEP70, GNAQ, GRIA1, GRIN2A, GRIN2B, GRM1, GRM5, ITPR1, ITPR2, PIK3CG, PLA2G5, PLA2G6, PLCB1, PLK4, PPP2R1A, PPP3CA, PRKCA, PRKX, PTEN, RAF1, RPS6KA2* |
| DNM1L | enzyme | 9.50 × 10^-3^ | *EGFR, GRIA1, PDGFRB, PPP3CA* |
| DTNBP1 | other | 9.60 × 10^-3^ | *GRIN2A, GRIN2B* |
| omeprazole | chemical drug | 9.80 × 10^-3^ | *ADCY2, CALML5, CAMK2A, CAMK2B, CAMK4, CDK1, CEP164, CEP70, E2F3, EGFR, GRIN2A, GRM1, HSP90AA1, ITPR2, PDGFRB, PIK3CA, PIK3CG, PLA2G6, PLK4, PPP3CA, PRKCB, PRKX, PTEN, RAF1, RPS6KA2* |
| IMPDH2 | enzyme | 9.90 × 10^-3^ | ***AFF3****, AKAP9, CALML5, CAMK2A, CAMK2B, CAMK2D, CAMK4, CDK6, CEP70, GNAQ, GRIN2A, GRIN2B, GRM1, GRM5, ITPR2, PIK3CG, PLA2G5, PLA2G6, PLCB1, PLK4, PPP2R1A, PPP3CA, PRKCB, PRKX, RPS6KA3, YWHAG* |
| YAP1 | transcription regulator | 9.90 × 10^-3^ | *CDK6, EGFR, PTEN* |

^1^Master regulators are molecules that indirectly control multiple genes in a pathway within the Ingenuity Pathway Analysis.

^2^Molecule type of the master regulator as defined by the Ingenuity Pathway Analysis.

^3^Network bias corrected *P* - value calculated by Ingenuity Pathway Analysis.

^4^ List of the positional candidate genes from the genome-wide association analysis (in **bold**) and leading edge genes from the gene-set enrichment analysis-SNP regulated by the master regulators.
