# Supplementary material for: Genomic Analysis of Spontaneous Abortion in Holstein Heifers and Primiparous Cows
Source: Genes (Basel). 2019 Nov 21;10(12):954. doi: 10.3390/genes10120954 (PMC6969913; doi:10.3390/genes10120954)
Supplement: Supplementary file 1 [file genes-10-00954-s001.zip › Supplemental Tables/Supplemental table 5- Combined Population Upstream Regulators.docx]

**Table S5:** Upstream regulators identified by Ingenuity Pathway Analysis with inputs of positional candidates and leading edge genes associated with spontaneous abortion in the combined Holstein heifer and cow population.

| **Upstream Regulator^1^** | **Molecule Type^2^** | ***P* –value^3^** | **Positional Candidate and Leading Edge Genes^4^** |
| --- | --- | --- | --- |
| camptothecin | chemical drug | 1.44 × 10^-7^ | *AKAP9, CAMK2B,* ***CAMK2G****, CDK1, CDK6, EGFR, GNAQ, ITPR1, MNAT1, NUMA1, PIK3CA, PIK3CG, PPP3CA, PTEN* |
| HTT | transcription regulator | 5.81 × 10^-6^ | *CAMK2A, CAMK2B, CAMK4, GRIA1, GRIN2A, GRIN2B, GRM1, ITPR1, PIK3CA, PLCB1, PPP3CA, PRKCB, YWHAG* |
| FSH | complex | 1.47 × 10^-5^ | *ADCY9,* ***CAMK2G****, CAMK4, CDK6, EGFR, GRIN2A, ITPR1, PPP2R1A, PRKX, PTEN* |
| SLITRK5 | other | 1.47 × 10^-5^ | *GRIA1, GRIN2A, GRIN2B* |
| maslinic acid | chemical - endogenous non-mammalian | 1.47 × 10^-5^ | ***CAMK2G****, CDK6, ITPR2, PRKCB, RPS6KA2, YWHAG* |
| HOMER2 | other | 6.00 × 10^-5^ | *GRIN2A, GRM1, GRM5* |
| NR3C1 | ligand-dependent nuclear receptor | 9.44 × 10^-5^ | *GRIA1, GRIN2A, GRIN2B, GRM1, GRM5, PPP3CA, PPP3CC, PRKCB, PTEN, RAF1, YWHAG* |
| BDNF | growth factor | 1.18 × 10^-4^ | *CAMK2A, GNAQ, GRIA1, GRIN2A, GRIN2B, ITPR1, PTEN, YWHAG* |
| mir-132 | microRNA | 1.18 × 10^-4^ | *GRIA1, GRIN2A, GRIN2B, PTEN* |
| caspase | group | 1.20 × 10^-4^ | *CDK1, CDK6, EGFR, PTEN* |
| TP53 | transcription regulator | 1.20 × 10^-4^ | *AKAP9, CAMK2B, CAMK2D, CDK1, CEP164, EGFR, GNAQ, HSP90AA1, PDGFRB, PIK3CG, PPP3CA, PRKCA, PRKCB, PTEN, RAF1, RPS6KA2, YWHAG* |
| APP | other | 1.21 × 10^-4^ | *CAMK2D, CDK1, CDK6, EGFR, GRIA1, GRIN2A, GRIN2B, HSP90AA1, PDGFRB, PPP2R1A, PRKCA, PRKCB* |
| ADAM10 | peptidase | 2.46 × 10^-4^ | *CAMK2A, EGFR, GNAQ, GRIN2A, GRIN2B* |
| FAS | transmembrane receptor | 2.69 × 10^-4^ | *AKAP9, CAMK2B,* ***CAMK2G****, ITPR1, NUMA1, PIK3CA, PIK3CG, PRKCB* |
| GRIN1 | ion channel | 3.39 × 10^-4^ | *CAMK2A, GRIN2A, GRIN2B* |
| miR-132-3p (and other miRNAs w/seed AACAGUC) | mature microRNA | 4.04 × 10^-4^ | *GRIA1, GRIN2A, GRIN2B* |
| SP1 | transcription regulator | 5.95 × 10^-4^ | *CDK1, CDK6, EGFR, GRIA1, GRM1, ITPR1, PDGFRB, PRKCA, PRKCB* |
| NMDA Receptor | complex | 7.99 × 10^-4^ | *CAMK2A, GRIA1, GRIN2A* |
| CPEB3 | translation regulator | 1.12 × 10^-3^ | *EGFR, GRIA1* |
| BAIAP2 | kinase | 1.12 × 10^-3^ | *GRIN2A, GRIN2B* |
| epalrestat | chemical drug | 1.12 × 10^-3^ | *PDGFRB, PRKCB* |
| morphine | chemical drug | 1.25 × 10^-3^ | *EGFR, GRIA1, GRIN2A, GRIN2B, PTEN* |
| HRAS | enzyme | 1.25 × 10^-3^ | *CDK1, EGFR, HSP90AA1, PDGFRB, PRKCA, PRKCB, PTEN, RAF1, RPS6KA2* |
| phenylephrine | chemical drug | 1.31 × 10^-3^ | *E2F3, EGFR, ITPR2, PRKCB* |
| Lh | complex | 1.62 × 10^-3^ | *ADCY9,* ***CAMK2G****, EGFR, ITPR1, PPP2R1A, PRKX* |
| 26s Proteasome | complex | 1.63 × 10^-3^ | *CDK1, EGFR, GRIA1, PRKCA, PTEN* |
| D-2-amino-5-phosphonovaleric acid | chemical reagent | 1.63 × 10^-3^ | *CAMK2B, GRIN2B* |
| BOK | other | 1.63 × 10^-3^ | *ITPR1, ITPR2* |
| DLG3 | kinase | 1.63 × 10^-3^ | *GRIN2A, GRIN2B* |
| CREBBP | transcription regulator | 1.81 × 10^-3^ | *CAMK2B, CAMK2D,* ***CAMK2G****, CAMK4, GRIN2A, GRM1, PDGFRB* |
| ESR1 | ligand-dependent nuclear receptor | 2.44 × 10^-3^ | *CAMK2B, CDK1, CDK6, CEP70, EGFR, GNAQ, HSP90AA1, PLCB1, PLK4, PPP2R1A, PRKX, PTEN* |
| DNMT3B | enzyme | 2.56 × 10^-3^ | *CAMK2A, CAMK4, CDK1, ITPR2, PRKCB* |
| TGFB1 | growth factor | 2.56 × 10^-3^ | *ADCY9,* ***CAMK2G****, CDK1, GRIA1, GRIN2A, GRIN2B, HSP90AA1, ITPR1, ITPR2, PDGFRB, PLCB1, PRKCA, PTEN, RAP1A* |
| tretinoin | chemical - endogenous mammalian | 3.09 × 10^-3^ | *CAMK4, CDK6, EGFR, GRIA1, ITPR2, MNAT1, PDGFRB, PIK3CG, PPP3CA, PRKCA, PRKCB, PTEN, TUBGCP5* |
| ADCY5 | enzyme | 3.26 × 10^-3^ | *ADCY2, ADCY9* |
| ingenol mebutate | chemical drug | 3.26 × 10^-3^ | *PRKCA, PRKCB* |
| CRNDE | other | 3.30 × 10^-3^ | *E2F3, EGFR, PDGFRB* |
| cyclosporin A | biologic drug | 3.45 × 10^-3^ | *EGFR, HSP90AA1, ITPR1, ITPR2, PRKCA, PTEN, RPS6KA2* |
| EDN1 | cytokine | 3.91 × 10^-3^ | *CDK1, EGFR, ITPR2, PRKCA, PRKCB* |
| ganetespib | chemical drug | 3.91 × 10^-3^ | *CDK1, EGFR* |
| HTR2A | G-protein coupled receptor | 3.91 × 10^-3^ | *EGFR, GRM1* |
| flupenthixol | chemical drug | 3.91 × 10^-3^ | *GRIN2A, GRIN2B* |
| beta-estradiol | chemical - endogenous mammalian | 4.46 × 10^-3^ | *ADCY9, CAMK2A, CAMK4, CDK1, E2F3, EGFR, GRIA1, GRIN2A, GRM5, PDGFRB, PPP3CA, PRKCB, PTEN, RAP1A* |
| PHLPP2 | enzyme | 4.65 × 10^-3^ | *PRKCA, PRKCB* |
| COMT | enzyme | 4.65 × 10^-3^ | *CAMK2A, CAMK4* |
| MDL 28170 | chemical toxicant | 4.65 × 10^-3^ | *GRIA1, GRM1* |
| dihematoporphyrin ether | chemical drug | 4.65 × 10^-3^ | *EGFR, RAF1* |
| U73122 | chemical reagent | 4.66 × 10^-3^ | *E2F3, ITPR2, PLCB1* |
| YAP1 | transcription regulator | 4.80 × 10^-3^ | *CDK1, CDK6, EGFR, PTEN* |
| mir-145 | microRNA | 4.80 × 10^-3^ | *CDK6, E2F3, EGFR* |
| cholecalciferol | chemical - endogenous mammalian | 4.80 × 10^-3^ | *EGFR, PDGFRB, PRKCB, PRKX* |
| MECP2 | transcription regulator | 4.80 × 10^-3^ | *CAMK2A, CAMK2B, GRIA1, GRIN2A* |
| 8,9-epoxyeicosatrienoic acid | chemical - endogenous mammalian | 4.80 × 10^-3^ | *EGFR, PIK3CG* |
| voltag × 10^-^gated calcium channel | complex | 4.80 × 10^-3^ | *ITPR1, ITPR2* |
| miR-142-3p (and other miRNAs w/seed GUAGUGU) | mature microRNA | 4.80 × 10^-3^ | *ADCY9, PRKCA* |
| PDYN | transporter | 4.80 × 10^-3^ | *GRM1, GRM5* |
| SLC30A3 | transporter | 4.80 × 10^-3^ | *GRIN2A, GRIN2B* |
| curcumin | chemical drug | 4.80 × 10^-3^ | *CDK1, CDK6, EGFR, GRIN2B, PDGFRB, PTEN* |
| NGF | growth factor | 4.80 × 10^-3^ | *CDK1, EGFR, GRIN2A, GRIN2B, PTEN* |
| testosterone | chemical - endogenous mammalian | 4.85 × 10^-3^ | *CAMK4, CDK6, EGFR, GRIA1, PRKCB, RAF1* |
| HDAC4 | transcription regulator | 5.38 × 10^-3^ | *CAMK2A, PPP3CA, PRKCA, PRKCB* |
| docetaxel | chemical drug | 5.46 × 10^-3^ | *CDK1, CDK6, EGFR, PRKCA* |
| ZGPAT | transcription regulator | 5.47 × 10^-3^ | *EGFR, PTEN* |
| USP8 | peptidase | 5.47 × 10^-3^ | *EGFR, GRIA1* |
| MYB | transcription regulator | 5.53 × 10^-3^ | *CAMK2B, CDK1, ITPR1, PPP3CA* |
| G2535 | chemical reagent | 6.38 × 10^-3^ | *EGFR, PTEN* |
| adaphostin | chemical drug | 6.38 × 10^-3^ | *CDK6, RAF1* |
| miR-34a-5p (and other miRNAs w/seed GGCAGUG) | mature microRNA | 6.39 × 10^-3^ | *CDK1, CDK6, E2F3* |
| PSEN1 | peptidase | 6.85 × 10^-3^ | *CAMK2D, EGFR, GRIN2A, GRIN2B, HSP90AA1, PDGFRB* |
| Pln | other | 6.90 × 10^-3^ | *PRKCA, PRKCB, RAP1A* |
| CAV1 | transmembrane receptor | 7.07 × 10^-3^ | *GRIN2A, GRIN2B, GRM1, PTEN* |
| N-methyl-D-aspartate | chemical drug | 7.07 × 10^-3^ | *GRIA1, GRIN2A, GRM1* |
| LDL | complex | 7.40 × 10^-3^ | *CAMK2B, CDK1, ITPR1, ITPR2, PTEN* |
| PDGFRB | kinase | 8.06 × 10^-3^ | *EGFR, PDGFRB* |
| CDC37 | kinase | 8.06 × 10^-3^ | *CDK6, RAF1* |
| CD247 | transmembrane receptor | 8.15 × 10^-3^ | *HSP90AA1, ITPR1, PLA2G5* |
| PLN | transporter | 8.83 × 10^-3^ | *PRKCA, PRKCB, RAP1A* |
| diacylglycerol | chemical - endogenous mammalian | 9.03 × 10^-3^ | *PRKCA, PRKCB* |
| SGK1 | kinase | 9.19 × 10^-3^ | *CDK6, GRIN2A, GRIN2B* |
| sulindac | chemical drug | 9.19 × 10^-3^ | *CDK1, E2F3, EGFR* |
| caffeine | chemical drug | 9.19 × 10^-3^ | *CDK1, EGFR, PRKCA* |
| mir-15 | microRNA | 9.49 × 10^-3^ | *CDK6, EGFR, RAF1* |
| 3,3'-diindolylmethane | chemical drug | 9.79 × 10^-3^ | *CDK6, EGFR, PTEN* |
| FANCC | other | 9.86 × 10^-3^ | *CAMK2B, CDK1, PRKCB* |
| ANXA7 | ion channel | 9.86 × 10^-3^ | *ITPR1, ITPR2, PTEN* |
| romidepsin | biologic drug | 9.86 × 10^-3^ | *EGFR, RAF1, RAP1A* |

^1^Upstream regulators directly control multiple genes in a pathway within the Ingenuity Pathway Analysis.

^2^Molecule type of the upstream regulator as defined by the Ingenuity Pathway Analysis.

^3^Network bias corrected *P*-value calculated by Ingenuity Pathway Analysis.

^4^List of the positional candidate genes from the genome-wide association analysis (in **bold**) and leading edge genes from the gene-set enrichment analysis-SNP regulated by the upstream regulator.
