# Supplementary material for: Genomic Analysis of Spontaneous Abortion in Holstein Heifers and Primiparous Cows
Source: Genes (Basel). 2019 Nov 21;10(12):954. doi: 10.3390/genes10120954 (PMC6969913; doi:10.3390/genes10120954)
Supplement: Supplementary file 1 [file genes-10-00954-s001.zip › Supplemental Tables/Supplemental table 3- Cow population Master Regulators.docx]

**Table S3:** Master regulators of positional candidate genes and leading edge genes associated with spontaneous abortion in the Holstein cow population.

| **Master Regulator^1^** | **Molecule Type^2^** | ***P*-value^3^** | **Positional Candidate and Leading Edge Genes^4^** |
| --- | --- | --- | --- |
| Type 2 Osm Receptor | complex | 1.00 × 10^-4^ | ***AFF3****, CHDH, CTH, DDHD1, ENPP2, LIPE, NEU3, PAFAH1B1, PAFAH1B3, PEX13, PLA2G2A, SMPDL3A* |
| TH1 Cytokine | group | 5.00 × 10^-4^ | ***AFF3****, CHDH, CTH, DDHD1, ENPP2,* ***GAB3****, LIPE,* ***NCALD****, NCEH1, NEU3, PAFAH1B1, PAFAH2, PEX13, PLA2G2A, PLCB1, SMPDL3A* |
| ALDH2 | enzyme | 5.00 × 10^-4^ | *CTH, PSPH* |
| TRIB3 | kinase | 6.00 × 10^-4^ | *CTH, PSPH* |
| CAB39 | enzyme | 7.00 × 10^-4^ | ***AFF3****, CHDH, CTH, ENPP2, LIPE,* ***NCALD****, NCEH1, PAFAH1B1, PAFAH1B3, PLA2G2A, PLCB1, SARDH, SMPDL3A* |
| PLCB4 | enzyme | 9.00 × 10^-4^ | *PLCB1* |
| PLCD1 | enzyme | 9.00 × 10^-4^ | *PLCB1* |
| IL12RB2 | transmembrane receptor | 1.10 × 10^-3^ | *CTH, ENPP2, PEX13, SMPDL3A* |
| PAFAH1B2 | enzyme | 1.10 × 10^-3^ | *PAFAH1B1* |
| PAFAH1B3 | enzyme | 1.10 × 10^-3^ | *PAFAH1B1* |
| prostaglandin A2 | chemical - endogenous non-mammalian | 1.20 × 10^-3^ | ***AFF3****, CHDH, CTH, DAO, LIPE,* ***NCALD****, NCEH1, PAFAH1B3, PAFAH2, PEX13, PLA2G2A, PLCB1, PSPH, SARDH* |
| entinostat | chemical drug | 1.50 × 10^-3^ | ***AFF3****, CHDH, CTH, DAO, ENPP2,* ***NCALD****, NCEH1, NEU3, PAFAH1B1, PAFAH1B3, PAFAH2, PEX13, PLA2G2A, PSPH* |
| 20-hydroxyeicosatetraenoic acid | chemical - endogenous mammalian | 1.50 × 10^-3^ | *CTH, LIPE, PLA2G2A, SARDH* |
| IL6 | cytokine | 1.60 × 10^-3^ | ***AFF3****, CHDH, CTH, ENPP2, LIPE,* ***NCALD****, NEU3, PAFAH1B1, PEX13, PLA2G2A, PLCB1, SARDH, SMPDL3A* |
| propyl gallate | chemical toxicant | 1.70 × 10^-3^ | *PLA2G2A* |
| sphingosin × 10^-^1-phosphate | chemical - endogenous mammalian | 1.90 × 10^-3^ | *ENPP2, PLA2G2A* |
| WSX1-gp130 | complex | 2.00 × 10^-3^ | *CHDH, CTH, DDHD1, ENPP2, LIPE,* ***NCALD****, NEU3, PAFAH1B1, PAFAH1B3, PEX13, SMPDL3A* |
| RBM3 | other | 2.10 × 10^-3^ | ***AFF3****, CTH, LIPE, PAFAH1B1, PLA2G2A, PSPH, SARDH, SMPDL3A* |
| HLA-B | transmembrane receptor | 2.20 × 10^-3^ | ***AFF3****, CTH, ENPP2, LIPE, PAFAH1B1, PLA2G2A, PSPH, SARDH, SMPDL3A* |
| 8(S)-hydroxyeicosatetraenoic acid | chemical - endogenous mammalian | 2.20 × 10^-3^ | *CTH, LIPE, PLA2G2A, SARDH* |
| NMB | other | 2.30 × 10^-3^ | ***AFF3****, DAO,* ***GAB3****, LIPE,* ***NCALD****, NCEH1, NEU3, PAFAH1B1, PAFAH1B3, PLCB1, PSPH, SARDH, SMPDL3A* |
| EIF2A | translation regulator | 2.40 × 10^-3^ | *CTH, LIPE, PLA2G2A, PSPH* |
| EBI3 | cytokine | 2.50 × 10^-3^ | ***AFF3****, CHDH, DDHD1, ENPP2,* ***NCALD****, NCEH1, NEU3, PAFAH1B1, PAFAH1B3, PAFAH2, PEX13, PLCB1, PSPH, SMPDL3A* |
| AG490 | chemical - kinase inhibitor | 2.50 × 10^-3^ | *ENPP2, PLA2G2A* |
| MYD88 | other | 2.60 × 10^-3^ | *CHDH, CTH, DAO, DDHD1, ENPP2,* ***GAB3****,* ***NCALD****, NCEH1, NEU3, PAFAH1B3, PAFAH2, PLA2G2A, SARDH, SMPDL3A* |
| EBF1-FOXO1-TCF3 | complex | 2.60 × 10^-3^ | ***AFF3****, CTH, DAO, ENPP2, LIPE, PLA2G2A, PSPH, SMPDL3A* |
| Ifn | group | 2.90 × 10^-3^ | ***AFF3****, CHDH, ENPP2, LIPE,* ***NCALD****, NCEH1, NEU3, PAFAH1B3, PEX13, PLA2G2A, PLCB1, PNLIPRP2, PSPH, SMPDL3A* |
| PRKD1 | kinase | 2.90 × 10^-3^ | *CHDH, CTH, DAO, ENPP2,* ***GAB3****, LIPE,* ***NCALD****, NCEH1, NEU3, PAFAH1B3, PAFAH2, PEX13, PLA2G2A, SMPDL3A* |
| DNAJ | group | 3.10 × 10^-3^ | *ENPP2* |
| HDAC5 | transcription regulator | 3.10 × 10^-3^ | ***AFF3****, DAO, ENPP2, PLA2G2A* |
| SC144 | chemical reagent | 3.10 × 10^-3^ | *ENPP2* |
| 2, 4-dinitrobenzenesulfonic acid | chemical reagent | 3.20 × 10^-3^ | *CTH* |
| ARFRP1 | enzyme | 3.50 × 10^-3^ | *LIPE* |
| saquinavir | chemical drug | 3.50 × 10^-3^ | *LIPE* |
| PPARD | ligand-dependent nuclear receptor | 3.60 × 10^-3^ | ***AFF3****, CHDH, CTH, DAO, ENPP2, LIPE,* ***NCALD****, NEU3, PAFAH2, PEX13, PLA2G2A, SARDH* |
| FR 49175 | chemical toxicant | 3.60 × 10^-3^ | *PAFAH1B3* |
| triamcinolone acetonide | chemical drug | 3.80 × 10^-3^ | *CTH, PEX13, PSPH* |
| CSF2 receptor | complex | 3.90 × 10^-3^ | *CHDH, CTH, DDHD1, ENPP2, LIPE, PAFAH1B1, PAFAH1B3, PEX13, SMPDL3A* |
| oleoylethanolamide | chemical - endogenous mammalian | 4.10 × 10^-3^ | ***AFF3****, CHDH, CTH,* ***NCALD****, NCEH1, NEU3, PAFAH2, PEX13, PLA2G2A, PSPH, SARDH* |
| Pro-inflammatory Cytokine | group | 4.30 × 10^-3^ | *CHDH, CTH, DAO, DDHD1, ENPP2,* ***GAB3****, LIPE,* ***NCALD****, NCEH1, NEU3, PAFAH1B1, PAFAH1B3, PAFAH2, PLA2G2A, PSPH, SMPDL3A* |
| zopolrestat | chemical drug | 4.40 × 10^-3^ | ***AFF3****, CHDH, ENPP2,* ***GAB3****, LIPE, PAFAH1B1, PAFAH1B3, PEX13, PLA2G2A, PLCB1, PSPH, SARDH, SMPDL3A* |
| rolipram | chemical drug | 4.50 × 10^-3^ | ***AFF3****, CTH, ENPP2,* ***GAB3****, LIPE,* ***NCALD****, NEU3, PAFAH1B3, PAFAH2, PEX13, PLA2G2A, PSPH, SMPDL3A* |
| edelfosine | chemical drug | 4.60 × 10^-3^ | ***AFF3****, CHDH, ENPP2, NCEH1, PAFAH1B1, PAFAH1B3, PAFAH2, PEX13, PLCB1, PSPH, SARDH, SMPDL3A* |
| nebivolol | chemical drug | 4.60 × 10^-3^ | ***AFF3****, CTH,* ***NCALD****, NCEH1, PAFAH1B3, PAFAH2, PLCB1, PNLIPRP2, PSPH, SARDH, SMPDL3A* |
| AMP | chemical - endogenous mammalian | 4.60 × 10^-3^ | *PNLIPRP2* |
| MEF2C | transcription regulator | 4.70 × 10^-3^ | ***AFF3****, CTH, DAO, ENPP2, LIPE,* ***NCALD****, PEX13, PLA2G2A, SMPDL3A* |
| UCP1 | transporter | 5.00 × 10^-3^ | ***AFF3****, CTH, ENPP2, LIPE, NCEH1, PAFAH1B1, PAFAH1B3, PLA2G2A, PLCB1, PSPH, SMPDL3A* |
| tyrphostin 25 | chemical - kinase inhibitor | 5.10 × 10^-3^ | ***AFF3****, CHDH, ENPP2,* ***NCALD****, NCEH1, NEU3, PAFAH1B1, PAFAH1B3, PEX13, PLA2G2A, PLCB1, SARDH* |
| NR0B2 | ligand-dependent nuclear receptor | 5.20 × 10^-3^ | ***AFF3****, CHDH, CTH, ENPP2, LIPE,* ***NCALD****, NCEH1, NEU3, PAFAH2, PEX13, PLA2G2A, PLCB1, PNLIPRP2, SARDH* |
| MK-1642 | chemical reagent | 5.20 × 10^-3^ | *LIPE* |
| IL18 | cytokine | 5.40 × 10^-3^ | *CHDH, CTH, DAO, ENPP2,* ***GAB3****, LIPE,* ***NCALD****, NCEH1, NEU3, PAFAH2, PEX13, PLA2G2A, PNLIPRP2, SMPDL3A* |
| AMPK | complex | 5.40 × 10^-3^ | ***AFF3****, CTH, ENPP2, LIPE, NCEH1, PAFAH1B1, PAFAH1B3, PLA2G2A, PLCB1, SMPDL3A* |
| SOCS | group | 5.60 × 10^-3^ | *CHDH, CTH, DDHD1, ENPP2, LIPE,* ***NCALD****, NEU3, PAFAH1B1, PAFAH1B3, PLCB1, SARDH* |
| berberine | chemical drug | 5.60 × 10^-3^ | ***AFF3****, CHDH, CTH, ENPP2, PAFAH1B3, PEX13, PLA2G2A, SARDH, SMPDL3A* |
| IL23A | cytokine | 5.70 × 10^-3^ | *CHDH, DDHD1, ENPP2,* ***NCALD****, NCEH1, NEU3, PAFAH1B1, PAFAH1B3, PAFAH2, PEX13, PNLIPRP2, PSPH, SMPDL3A* |
| momelotinib | chemical drug | 5.70 × 10^-3^ | *CHDH, CTH, DDHD1, ENPP2, LIPE, PAFAH1B1, PAFAH1B3, PEX13, SMPDL3A* |
| filgotinib | chemical drug | 5.70 × 10^-3^ | *CHDH, CTH, DDHD1, ENPP2, LIPE, PAFAH1B1, PAFAH1B3, PEX13, SMPDL3A* |
| SAR-20347 | chemical - kinase inhibitor | 5.80 × 10^-3^ | *CHDH, CTH, DDHD1, ENPP2, LIPE, PAFAH1B1, PAFAH1B3, PEX13, SMPDL3A* |
| MitoBloCK-6 | chemical reagent | 5.90 × 10^-3^ | *PSPH* |
| OPRD1 | G-protein coupled receptor | 6.00 × 10^-3^ | ***AFF3****, CHDH, DAO,* ***GAB3****,* ***NCALD****, NCEH1, PAFAH1B1, PAFAH1B3, PLCB1, PSPH, SARDH, SMPDL3A* |
| nordihydroguaiaretic acid | chemical drug | 6.00 × 10^-3^ | *PLA2G2A* |
| ibuprofen | chemical drug | 6.20 × 10^-3^ | ***AFF3****, CHDH, CTH, DAO, ENPP2,* ***GAB3****, LIPE, NEU3, PAFAH1B3, PAFAH2, PLA2G2A, SARDH* |
| C5AR1 | G-protein coupled receptor | 6.30 × 10^-3^ | *CHDH, DAO, ENPP2,* ***GAB3****, LIPE,* ***NCALD****, NCEH1, NEU3, PAFAH1B1, PAFAH1B3, PAFAH2, PEX13, PSPH* |
| evodiamine | chemical - endogenous non-mammalian | 6.40 × 10^-3^ | *LIPE* |
| aleglitazar | chemical drug | 6.50 × 10^-3^ | *CTH, LIPE, PLA2G2A, SARDH* |
| tesaglitazar | chemical drug | 6.50 × 10^-3^ | *CTH, LIPE, PLA2G2A, SARDH* |
| isohumulone | chemical - endogenous non-mammalian | 6.50 × 10^-3^ | *CTH, LIPE, PLA2G2A, SARDH* |
| DUT | enzyme | 6.50 × 10^-3^ | *CTH, LIPE, PLA2G2A, SARDH* |
| berberine | chemical drug | 6.60 × 10^-3^ | ***AFF3****, CHDH, CTH, DAO, DDHD1, ENPP2,* ***GAB3****, NEU3, PAFAH1B3, PEX13, PLA2G2A, SARDH, SMPDL3A* |
| monooleylphosphatidic acid | chemical - endogenous mammalian | 6.60 × 10^-3^ | *ENPP2* |
| GPR39 | G-protein coupled receptor | 6.80 × 10^-3^ | *LIPE* |
| AMPK | complex | 6.90 × 10^-3^ | ***AFF3****, CTH, ENPP2,* ***GAB3****, LIPE,* ***NCALD****, NCEH1, PAFAH1B1, PAFAH1B3, PLA2G2A, PLCB1, PNLIPRP2, SMPDL3A* |
| farglitazar | chemical drug | 6.90 × 10^-3^ | *CTH, LIPE, PLA2G2A, SARDH* |
| 13-hydroxyoctadecadienoic acid | chemical - endogenous mammalian | 6.90 × 10^-3^ | *CTH, LIPE, PLA2G2A, SARDH* |
| 9-hydroxyoctadecadienoic acid | chemical - endogenous mammalian | 6.90 × 10^-3^ | *CTH, LIPE, PLA2G2A, SARDH* |
| SRPK1 | kinase | 7.10 × 10^-3^ | *CHDH, CTH, ENPP2,* ***GAB3****, LIPE,* ***NCALD****, NCEH1, NEU3, PEX13, PLCB1, PNLIPRP2, PSPH, SMPDL3A* |
| PSAP | enzyme | 7.10 × 10^-3^ | ***AFF3****, CHDH,* ***GAB3****, LIPE,* ***NCALD****, NCEH1, PAFAH1B1, PAFAH1B3, PLCB1, PSPH, SARDH, SMPDL3A* |
| MEDAG | other | 7.10 × 10^-3^ | *LIPE* |
| mir-17 | microRNA | 7.30 × 10^-3^ | ***AFF3****, DAO, DDHD1, ENPP2,* ***NCALD****, NEU3, PAFAH2, PEX13, PLA2G2A, PLCB1, PSPH, SARDH, SMPDL3A* |
| baricitinib | chemical drug | 7.30 × 10^-3^ | *CHDH, CTH, DDHD1, ENPP2, LIPE, PAFAH1B1, PAFAH1B3, PEX13, SMPDL3A* |
| PPARÎ±-RXRÎ± | complex | 7.30 × 10^-3^ | *CTH, ENPP2, NEU3, PLA2G2A, SARDH* |
| AXL | kinase | 7.40 × 10^-3^ | ***AFF3****, CTH, DAO,* ***GAB3****, LIPE,* ***NCALD****, NCEH1, NEU3, PAFAH1B1, PAFAH1B3, PAFAH2, PEX13, PLCB1, PSPH* |
| nelfinavir | chemical drug | 7.40 × 10^-3^ | *LIPE* |
| 5(S)-HETE | chemical - endogenous mammalian | 7.60 × 10^-3^ | ***AFF3****, CHDH, ENPP2,* ***GAB3****, LIPE,* ***NCALD****, NCEH1, PAFAH1B1, PAFAH1B3, PEX13, PLCB1, SARDH, SMPDL3A* |
| CBP-CREB-CRTC2 | complex | 7.70 × 10^-3^ | ***AFF3****, CTH, LIPE,* ***NCALD****, NCEH1, PAFAH1B1, PLCB1, PNLIPRP2, PSPH, SARDH* |
| tosylphenylalanyl chloromethyl ketone | chemical - protease inhibitor | 7.80 × 10^-3^ | ***AFF3****, CHDH, DAO, ENPP2,* ***NCALD****, NCEH1, NEU3, PAFAH1B3, PAFAH2, PEX13, PSPH* |
| 12-(3-adamantan-1-yl-ureido) dodecanoic acid | chemical reagent | 7.80 × 10^-3^ | *CTH, LIPE, PLA2G2A, SARDH* |
| ZFP36 | transcription regulator | 8.10 × 10^-3^ | *CHDH, ENPP2,* ***GAB3****, LIPE, NCEH1, NEU3, PAFAH1B3, PAFAH2, PLA2G2A, PLCB1, PSPH, SARDH, SMPDL3A* |
| pioglitazone | chemical drug | 8.20 × 10^-3^ | *CTH, DAO, ENPP2, LIPE,* ***NCALD****, NCEH1, NEU3, PAFAH1B1, PEX13, PLA2G2A, PLCB1, PSPH* |
| propranolol | chemical drug | 8.30 × 10^-3^ | *CHDH, CTH, DAO, ENPP2,* ***GAB3****, LIPE,* ***NCALD****, NCEH1, NEU3, PAFAH1B3, PEX13, PLA2G2A* |
| Laminin1 | complex | 8.60 × 10^-3^ | ***AFF3****, CHDH, CTH, NCEH1, NEU3, PLCB1, PSPH, SARDH, SMPDL3A* |
| di(2-ethylhexyl) phthalate | chemical toxicant | 8.80 × 10^-3^ | *CTH, ENPP2, LIPE, PLA2G2A, SARDH* |
| PPARA | ligand-dependent nuclear receptor | 9.00 × 10^-3^ | ***AFF3****, CTH, DAO, LIPE, NCEH1, NEU3, PEX13, PLA2G2A, PSPH, SARDH, SMPDL3A* |
| L-serine | chemical - endogenous mammalian | 9.10 × 10^-3^ | *PSPH* |
| choline fenofibrate | chemical drug | 9.20 × 10^-3^ | *CTH, LIPE, PLA2G2A, SARDH* |
| PECAM1 | other | 9.50 × 10^-3^ | ***AFF3****, CHDH, ENPP2,* ***NCALD****, NCEH1, NEU3, PAFAH1B1, PAFAH1B3, PAFAH2, PEX13, PLCB1, SARDH* |
| Gsk3 | group | 9.60 × 10^-3^ | *CTH, DAO, DDHD1,* ***GAB3****,* ***NCALD****, NEU3, PAFAH1B1, PAFAH1B3, PAFAH2, PLA2G2A, PNLIPRP2, PSPH, SARDH, SMPDL3A* |
| CMKLR1 | G-protein coupled receptor | 9.60 × 10^-3^ | *LIPE* |
| CXCL3 | cytokine | 9.80 × 10^-3^ | *CTH, ENPP2, LIPE,* ***NCALD****, NCEH1, NEU3, PAFAH1B1, PAFAH1B3, PAFAH2, PEX13, PLA2G2A, PSPH* |
| clofibric acid | chemical drug | 9.80 × 10^-3^ | *CTH, LIPE, PLA2G2A, SARDH* |
| methyl-beta-cyclodextrin | chemical drug | 9.90 × 10^-3^ | ***AFF3****, CHDH, ENPP2,* ***GAB3****, LIPE,* ***NCALD****, NCEH1, NEU3, PAFAH1B3, PAFAH2, PEX13, PLA2G2A* |
| PRKAR2B | kinase | 9.90 × 10^-3^ | ***AFF3****, CTH, LIPE,* ***NCALD****, NCEH1, PAFAH1B1, PLA2G2A, PLCB1, PSPH, SARDH, SMPDL3A* |

^1^Master regulators are molecules that indirectly control multiple genes in a pathway within the Ingenuity Pathway Analysis.

^2^Molecule type of the master regulator as defined by the Ingenuity Pathway Analysis.

^3^Network bias corrected *P* - value calculated by Ingenuity Pathway Analysis.

^4^ List of the positional candidate genes from the genome-wide association analysis (in **bold**) and leading edge genes from the gene-set enrichment analysis-SNP regulated by the master regulators.
