# Supplementary material for: Genomic Analysis of Spontaneous Abortion in Holstein Heifers and Primiparous Cows
Source: Genes (Basel). 2019 Nov 21;10(12):954. doi: 10.3390/genes10120954 (PMC6969913; doi:10.3390/genes10120954)
Supplement: Supplementary file 1 [file genes-10-00954-s001.zip › Supplemental Tables/Supplemental table 1- Heifer Population Master Regulators.docx]

**Table S1:** Master regulators of positional candidate genes and leading-edge genes associated with spontaneous abortion in the Holstein heifer population.

| **Master Regulator^1^** | **Molecule Type^2^** | ***P*-value^3^** | **Positional Candidate and Leading-edge Genes^4^** |
| --- | --- | --- | --- |
| PDGF (family) | group | 2.00 × 10^-4^ | ***ACOT12****, ASCC3, AURKA, BABAM2, BANP,* ***CAMK2G****, CENPH, EYA2,* ***GAB3****, HMG20B, MCM3, POLE3, PRKCB,* ***RBL2****, RRS1, USP15, VPS72* |
| CAPN1 | peptidase | 2.00 × 10^-4^ | ***ACOT12****,* ***AFF3****, ASCC3, AURKA, BABAM2, BANP,* ***CAMK2G****, CENPH, HMG20B, MCM3,* ***PTPN14****,* ***RBL2****, RRS1, USP15, VPS72* |
| erlotinib | chemical drug | 3.00 × 10^-4^ | ***ACOT12****,* ***AFF3****, ASCC3, AURKA, BABAM2, BANP,* ***CAMK2G****, CENPH, EYA2, HMG20B, MCM3, POLE3,* ***PTPN14****,* ***RBL2****, RRS1, USP15* |
| canertinib | chemical drug | 4.00 × 10^-4^ | ***ACOT12****,* ***AFF3****, ASCC3, AURKA, BABAM2,* ***CAMK2G****, CENPH, EYA2, HMG20B, MCM3, POLE3, PRKCB,* ***PTPN14****,* ***RBL2****, USP15* |
| fulvestrant | chemical drug | 4.00 × 10^-4^ | *ASCC3, AURKA, MCM3,* ***RBL2*** |
| TNFRSF4 | transmembrane receptor | 5.00 × 10^-4^ | ***ACOT12****,* ***AFF3****, ASCC3, BABAM2, BANP,* ***CAMK2G****, CENPH, EYA2, HMG20B, POLE3, PRKCB,* ***RBL2****, RRS1, USP15* |
| AEE 788 | chemical drug | 6.00 × 10^-4^ | ***ACOT12****,* ***AFF3****, ASCC3, AURKA, BABAM2,* ***CAMK2G****, CENPH, EYA2, HMG20B, MCM3, POLE3, PRKCB,* ***RBL2****, USP15, VPS72* |
| PI 3-kinase inhibitor | chemical drug | 6.00 × 10^-4^ | ***AFF3****, AURKA, BABAM2, BANP, EYA2,* ***GAB3****, HMG20B, MCM3, PRKCB,* ***PTPN14****,* ***RBL2****, USP15, VPS72* |
| CUL5 | ion channel | 6.00 × 10^-4^ | ***ACOT12****,* ***AFF3****, ASCC3, AURKA, BABAM2,* ***CAMK2G****, CENPH, EYA2, HMG20B, MCM3, POLE3, PRKCB,* ***RBL2****, RRS1, USP15* |
| PTPRB | phosphatase | 7.00 × 10^-4^ | ***ACOT12****, ASCC3, AURKA, BABAM2, BANP,* ***CAMK2G****, CENPH, EYA2,* ***GAB3****, HMG20B, MCM3, POLE3, PRKCB,* ***RBL2****, USP15, VPS72* |
| AREG | growth factor | 7.00 × 10^-4^ | ***AFF3****, ASCC3, AURKA, BABAM2, BANP,* ***CAMK2G****, EYA2,* ***GAB3****, MCM3, POLE3,* ***PTPN14****,* ***RBL2****, RRS1, USP15, VPS72* |
| BMI1 | transcription regulator | 7.00 × 10^-4^ | *AURKA, MCM3* |
| SDC1 | enzyme | 8.00 × 10^-4^ | ***ACOT12****, AURKA, BABAM2, BANP,* ***CAMK2G****, CENPH, EYA2, HMG20B, PRKCB,* ***PTPN14****,* ***RBL2****, USP15, VPS72* |
| WNK2 | kinase | 9.00 × 10^-4^ | ***ACOT12****,* ***AFF3****, BABAM2, BANP,* ***CAMK2G****, CENPH, EYA2, HMG20B, PRKCB,* ***RBL2****, RRS1, USP15, VPS72* |
| Evans Blue | chemical toxicant | 9.00 × 10^-4^ | ***ACOT12****,* ***AFF3****, ASCC3, AURKA, BABAM2, BANP,* ***CAMK2G****, EYA2,* ***GAB3****, HMG20B, PRKCB, RRS1, USP15, VPS72* |
| SB-431542 | chemical reagent | 1.00 × 10^-3^ | ***ACOT12****,* ***AFF3****, AURKA, BABAM2, BANP,* ***CAMK2G****, CENPH, EYA2,* ***GAB3****, HMG20B, PRKCB,* ***PTPN14****,* ***RBL2****, USP15* |
| PTPRJ | phosphatase | 1.00 × 10^-3^ | ***ACOT12****, ASCC3, AURKA, BABAM2,* ***CAMK2G****, CENPH, EYA2,* ***GAB3****, MCM3, POLE3, PRKCB,* ***RBL2****, RRS1, USP15, VPS72* |
| GABPB1 | transcription regulator | 1.00 × 10^-3^ | *AURKA,* ***RBL2*** |
| dacomitinib | chemical drug | 1.10 × 10^-3^ | ***ACOT12****, ASCC3, AURKA, BABAM2, BANP,* ***CAMK2G****, CENPH, EYA2, HMG20B, MCM3, POLE3, PRKCB,* ***RBL2****, RRS1, USP15* |
| CRKL | kinase | 1.20 × 10^-3^ | ***ACOT12****,* ***AFF3****, ASCC3, AURKA, BANP,* ***CAMK2G****, EYA2,* ***GAB3****, HMG20B, PRKCB,* ***PTPN14****, RNF20, USP15, VPS72* |
| putrescine | chemical - endogenous mammalian | 1.20 × 10^-3^ | ***ACOT12****,* ***AFF3****, AURKA, BABAM2, BANP,* ***CAMK2G****, EYA2,* ***GAB3****, PRKCB,* ***PTPN14****,* ***RBL2*** |
| CSF | group | 1.20 × 10^-3^ | *AURKA, CENPH,* ***GAB3****, MCM3, PRKCB* |
| NRG (family) | group | 1.30 × 10^-3^ | *ASCC3, AURKA, BANP,* ***CAMK2G****, EYA2,* ***GAB3****, HMG20B, MCM3, POLE3,* ***RBL2****, RNF20, RRS1, USP15* |
| neratinib | chemical drug | 1.30 × 10^-3^ | ***ACOT12****,* ***AFF3****, ASCC3, AURKA, BABAM2,* ***CAMK2G****, CENPH, EYA2, HMG20B, MCM3, POLE3, PRKCB,* ***RBL2****, USP15* |
| GABPA | transcription regulator | 1.30 × 10^-3^ | *AURKA,* ***RBL2*** |
| Igf | group | 1.40 × 10^-3^ | ***ACOT12****, ASCC3, AURKA, BABAM2, BANP,* ***CAMK2G****, CENPH, EYA2,* ***GAB3****, HMG20B, POLE3, PRKCB,* ***RBL2****, RRS1, USP15, VPS72* |
| Nrg1 | other | 1.40 × 10^-3^ | ***ACOT12****,* ***AFF3****, ASCC3, AURKA, BABAM2, BANP,* ***CAMK2G****, CENPH, EYA2, MCM3,* ***PTPN14****,* ***RBL2****, RRS1, USP15* |
| NRG3 | growth factor | 1.40 × 10^-3^ | ***ACOT12****, ASCC3, AURKA, BABAM2, BANP,* ***CAMK2G****, CENPH, EYA2, HMG20B, MCM3, PRKCB,* ***RBL2****, RRS1, USP15* |
| PPP1CA | phosphatase | 1.40 × 10^-3^ | ***AFF3****, AURKA, BABAM2, BANP,* ***CAMK2G****, CENPH, EYA2,* ***GAB3****, HMG20B, MCM3, PRKCB,* ***PTPN14****,* ***RBL2*** |
| dihematoporphyrin ether | chemical drug | 1.40 × 10^-3^ | ***ACOT12****,* ***AFF3****, BABAM2, BANP,* ***CAMK2G****, CENPH, EYA2, HMG20B, MCM3, PRKCB,* ***RBL2****, RRS1, USP15* |
| NRG4 | growth factor | 1.50 × 10^-3^ | ***AFF3****, ASCC3, AURKA, BABAM2, BANP,* ***CAMK2G****, EYA2,* ***GAB3****, HMG20B, MCM3, PRKCB,* ***RBL2****, RRS1, USP15* |
| phytosphingosine | chemical - endogenous mammalian | 1.60 × 10^-3^ | ***AFF3****, AURKA, BABAM2, BANP,* ***CAMK2G****, EYA2,* ***GAB3****, HMG20B, PRKCB, USP15* |
| SAICAR | chemical - endogenous mammalian | 1.70 × 10^-3^ | ***ACOT12****,* ***AFF3****, AURKA, BABAM2,* ***CAMK2G****, EYA2,* ***GAB3****, HMG20B, MCM3, POLE3, PRKCB,* ***PTPN14****,* ***RBL2****, USP15, VPS72* |
| Metalloprotease | group | 1.70 × 10^-3^ | ***ACOT12****,* ***AFF3****, AURKA, BABAM2, BANP,* ***CAMK2G****, CENPH, EYA2,* ***GAB3****, HMG20B, POLE3, PRKCB,* ***PTPN14****,* ***RBL2****, RRS1* |
| FGF3 | growth factor | 1.70 × 10^-3^ | ***ACOT12****,* ***AFF3****, AURKA, BABAM2, BANP,* ***CAMK2G****, CENPH, EYA2,* ***GAB3****, HMG20B, MCM3, PRKCB,* ***RBL2****, USP15, VPS72* |
| ERBB3 | kinase | 1.80 × 10^-3^ | *ASCC3, AURKA, BABAM2, BANP,* ***CAMK2G****, CENPH,* ***GAB3****, HMG20B, MCM3, POLE3, PRKCB,* ***RBL2****, RRS1, USP15, VPS72* |
| atropine | chemical drug | 1.80 × 10^-3^ | ***ACOT12****,* ***AFF3****, AURKA, BABAM2, BANP,* ***CAMK2G****, CENPH, EYA2, HMG20B, MCM3, PRKCB,* ***RBL2****, RRS1, USP15* |
| midazolam | chemical drug | 1.80 × 10^-3^ | ***ACOT12****,* ***AFF3****, AURKA, BABAM2, BANP,* ***CAMK2G****, CENPH,* ***GAB3****, HMG20B, PRKCB, USP15* |
| ANK2 | other | 1.90 × 10^-3^ | ***ACOT12****,* ***AFF3****, AURKA, BABAM2, BANP,* ***CAMK2G****, CENPH, EYA2,* ***GAB3****, HMG20B, PRKCB, RRS1, USP15* |
| ODC1 | enzyme | 2.00 × 10^-3^ | ***ACOT12****,* ***AFF3****, ASCC3, BABAM2,* ***CAMK2G****, CENPH, EYA2, HMG20B, PRKCB,* ***PTPN14****,* ***RBL2****, RNF20, RRS1, USP15, VPS72* |
| RASD1 | enzyme | 2.00 × 10^-3^ | ***ACOT12****,* ***AFF3****, AURKA, BABAM2, BANP,* ***CAMK2G****, CENPH,* ***GAB3****, HMG20B, PRKCB,* ***PTPN14****, RRS1, USP15* |
| PIK3CD | kinase | 2.10 × 10^-3^ | ***AFF3****, ASCC3, AURKA, BABAM2, BANP,* ***CAMK2G****, CENPH,* ***GAB3****, HMG20B, MCM3,* ***PTPN14****,* ***RBL2****, RRS1* |
| RBL1 | transcription regulator | 2.10 × 10^-3^ | *MCM3,* ***RBL2*** |
| PHB | transcription regulator | 2.20 × 10^-3^ | ***ACOT12****, AURKA, BABAM2, BANP, CENPH, EYA2, HMG20B, MCM3, PRKCB,* ***RBL2****, RRS1, USP15, VPS72* |
| AP20187 | chemical reagent | 2.30 × 10^-3^ | ***ACOT12****,* ***AFF3****, ASCC3, AURKA, BABAM2, BANP,* ***CAMK2G****, CENPH, EYA2,* ***GAB3****, HMG20B, PRKCB, RRS1, USP15* |
| CDH2 | other | 2.40 × 10^-3^ | ***ACOT12****,* ***AFF3****, ASCC3, AURKA, BABAM2, BANP,* ***CAMK2G****, CENPH, EYA2,* ***GAB3****, PRKCB,* ***RBL2****, RRS1, USP15* |
| binimetinib | chemical drug | 2.40 × 10^-3^ | ***ACOT12****,* ***AFF3****, AURKA, BABAM2, BANP,* ***CAMK2G****, CENPH, EYA2,* ***GAB3****, HMG20B, PRKCB, RRS1, USP15, VPS72* |
| golvatinib | chemical drug | 2.40 × 10^-3^ | ***ACOT12****, ASCC3, AURKA, BABAM2, BANP,* ***CAMK2G****, CENPH, EYA2, HMG20B, PRKCB, RRS1, USP15, VPS72* |
| GRPR | G-protein coupled receptor | 2.50 × 10^-3^ | ***ACOT12****,* ***AFF3****, AURKA, BANP,* ***CAMK2G****, EYA2,* ***GAB3****, HMG20B, PRKCB, RNF20, RRS1, USP15, VPS72* |
| TGM2 | enzyme | 2.60 × 10^-3^ | ***ACOT12****, ASCC3, AURKA, BABAM2, BANP,* ***CAMK2G****, CENPH, EYA2, HMG20B, MCM3, POLE3,* ***PTPN14****,* ***RBL2****, USP15, VPS72* |
| NMB | other | 2.60 × 10^-3^ | ***AFF3****, ASCC3, AURKA, BABAM2, BANP,* ***CAMK2G****, EYA2,* ***GAB3****, HMG20B, PRKCB, RRS1, USP15, VPS72* |
| FGF6 | growth factor | 2.60 × 10^-3^ | ***ACOT12****,* ***AFF3****, AURKA, BABAM2, BANP,* ***CAMK2G****, CENPH,* ***GAB3****, HMG20B, PRKCB, RRS1, USP15, VPS72* |
| MC1R | G-protein coupled receptor | 2.70 × 10^-3^ | ***AFF3****, AURKA, BABAM2, BANP,* ***CAMK2G****, EYA2,* ***GAB3****, HMG20B, RRS1, USP15* |
| pelitinib | chemical drug | 2.70 × 10^-3^ | ***ACOT12****, ASCC3, AURKA, BABAM2, BANP,* ***CAMK2G****, CENPH, HMG20B, MCM3, POLE3, PRKCB,* ***RBL2****, RRS1, USP15* |
| EPO | cytokine | 2.70 × 10^-3^ | ***ACOT12****,* ***AFF3****, ASCC3, AURKA,* ***CAMK2G****,* ***GAB3****, HMG20B, MCM3, PRKCB,* ***PTPN14****,* ***RBL2****, RNF20, RRS1, VPS72* |
| afatinib | chemical drug | 3.00 × 10^-3^ | ***ACOT12****, ASCC3, AURKA, BABAM2, BANP,* ***CAMK2G****, CENPH, EYA2, HMG20B, MCM3, POLE3,* ***RBL2****, RRS1, USP15* |
| PPP1R17 | other | 3.00 × 10^-3^ | ***ACOT12****,* ***AFF3****, AURKA, BABAM2, BANP,* ***CAMK2G****, CENPH,* ***GAB3****, HMG20B,* ***PTPN14****, USP15* |
| Calcineurin A | group | 3.00 × 10^-3^ | ***ACOT12****,* ***AFF3****, AURKA, BABAM2, BANP,* ***CAMK2G****,* ***GAB3****, HMG20B, MCM3,* ***RBL2****, RRS1, USP15* |
| CFLAR | other | 3.30 × 10^-3^ | ***AFF3****, AURKA, BANP,* ***CAMK2G****, EYA2,* ***GAB3****, HMG20B,* ***PTPN14****, RNF20, RRS1, USP15* |
| NOS3 | enzyme | 3.60 × 10^-3^ | *ASCC3, AURKA, BABAM2,* ***CAMK2G****, CENPH,* ***GAB3****, HMG20B, MCM3,* ***RBL2****, RNF20, RRS1, USP15, VPS72* |
| nilotinib | chemical drug | 3.70 × 10^-3^ | ***ACOT12****, ASCC3, AURKA, BABAM2, BANP,* ***CAMK2G****, CENPH, EYA2,* ***GAB3****, HMG20B,* ***PTPN14****, RRS1, VPS72* |
| RGS5 | other | 3.70 × 10^-3^ | ***AFF3****, AURKA, BABAM2, BANP,* ***CAMK2G****, EYA2,* ***GAB3****, HMG20B, RRS1, USP15* |
| YWHAE | other | 3.70 × 10^-3^ | ***ACOT12****,* ***AFF3****, ASCC3, BABAM2, BANP,* ***CAMK2G****, CENPH, EYA2, PRKCB,* ***RBL2****, RRS1, USP15, VPS72* |
| EREG | growth factor | 3.70 × 10^-3^ | ***ACOT12****, AURKA, BABAM2, BANP,* ***CAMK2G****, CENPH, EYA2, MCM3, POLE3, PRKCB,* ***RBL2****, RNF20, RRS1, USP15* |
| nocodazole | chemical reagent | 3.70 × 10^-3^ | *AURKA, PRKCB* |
| DIAPH1 | other | 4.20 × 10^-3^ | ***ACOT12****, AURKA, BABAM2, BANP,* ***CAMK2G****, CENPH, EYA2,* ***GAB3****, HMG20B, USP15* |
| GJA1 | transporter | 4.30 × 10^-3^ | ***ACOT12****,* ***AFF3****, ASCC3, BABAM2, BANP,* ***CAMK2G****, CENPH,* ***GAB3****, HMG20B, MCM3, PRKCB,* ***PTPN14****,* ***RBL2****, USP15, VPS72* |
| tyrphostin AG 1024 | chemical - kinase inhibitor | 4.30 × 10^-3^ | ***ACOT12****,* ***AFF3****, ASCC3, AURKA, BABAM2, BANP,* ***CAMK2G****, CENPH,* ***GAB3****, POLE3,* ***RBL2****, RRS1, USP15, VPS72* |
| Gd3+ | chemical - endogenous mammalian | 4.30 × 10^-3^ | ***ACOT12****,* ***AFF3****, AURKA, BABAM2, BANP,* ***CAMK2G****, CENPH,* ***GAB3****, HMG20B,* ***PTPN14****, USP15* |
| veratridine | chemical toxicant | 4.30 × 10^-3^ | ***ACOT12****, AURKA, BABAM2, BANP,* ***CAMK2G****, CENPH, EYA2,* ***GAB3****, HMG20B, USP15* |
| VEZF1 | transcription regulator | 4.40 × 10^-3^ | *USP15* |
| 4-methylnitrosoamino-1-(3-pyridinyl)-1-butanone | chemical toxicant | 4.50 × 10^-3^ | ***ACOT12****,* ***AFF3****, ASCC3, AURKA, BABAM2, BANP,* ***CAMK2G****,* ***GAB3****, PRKCB,* ***PTPN14****,* ***RBL2****, RRS1, USP15, VPS72* |
| 2-acetylaminofluorene | chemical toxicant | 4.70 × 10^-3^ | ***AFF3****, ASCC3, AURKA, BABAM2, BANP,* ***CAMK2G****, CENPH, EYA2, HMG20B, MCM3, PRKCB,* ***RBL2****, RRS1, USP15* |
| CDK14 | kinase | 4.80 × 10^-3^ | *AURKA, MCM3* |
| TRAF2-TRAF3 | complex | 4.90 × 10^-3^ | *AURKA, HMG20B, MCM3, POLE3,* ***PTPN14****,* ***RBL2****, RRS1* |
| calpeptin | chemical reagent | 5.10 × 10^-3^ | ***ACOT12****,* ***AFF3****, AURKA, BABAM2, BANP,* ***CAMK2G****,* ***GAB3****, HMG20B, MCM3, PRKCB,* ***PTPN14****,* ***RBL2****, USP15* |
| [Nle4, D-Phe7] -Alpha-MSH | biologic drug | 5.10 × 10^-3^ | ***ACOT12****,* ***AFF3****, AURKA, BABAM2, BANP,* ***CAMK2G****, CENPH, EYA2,* ***GAB3****, HMG20B, PRKCB, RRS1, USP15* |
| DL-threo-dihydrosphingosine | chemical - kinase inhibitor | 5.40 × 10^-3^ | ***AFF3****, AURKA, BABAM2, BANP,* ***CAMK2G****, CENPH,* ***GAB3****, HMG20B, PRKCB,* ***PTPN14****,* ***RBL2****, USP15* |
| ZHX2 | transcription regulator | 5.40 × 10^-3^ | ***ACOT12****,* ***AFF3****, AURKA, BABAM2, BANP,* ***CAMK2G****, CENPH, EYA2,* ***GAB3****, HMG20B, PRKCB, RRS1, USP15* |
| ergosterol-5, 8-peroxide | chemical - endogenous mammalian | 5.50 × 10^-3^ | ***ACOT12****,* ***AFF3****, AURKA, BABAM2, BANP,* ***CAMK2G****, CENPH, EYA2,* ***GAB3****, HMG20B, PRKCB, RRS1, USP15* |
| MAGED1 | transcription regulator | 5.50 × 10^-3^ | ***AFF3****, BABAM2, BANP, CENPH, EYA2, HMG20B, RNF20, RRS1, USP15* |
| AKAP12 | transporter | 5.70 × 10^-3^ | *AURKA, BABAM2, BANP,* ***CAMK2G****, EYA2,* ***GAB3****, HMG20B, MCM3,* ***PTPN14****,* ***RBL2****, VPS72* |
| LAMA2 | other | 5.70 × 10^-3^ | *AURKA,* ***CAMK2G****, EYA2, HMG20B, MCM3, PRKCB,* ***RBL2****, RRS1, USP15, VPS72* |
| N-ethylmaleimide | chemical reagent | 5.90 × 10^-3^ | ***ACOT12****,* ***AFF3****, ASCC3,* ***CAMK2G****, CENPH, HMG20B, POLE3, PRKCB,* ***PTPN14****,* ***RBL2****, RNF20, USP15, VPS72* |
| PD173074 | chemical reagent | 6.10 × 10^-3^ | ***ACOT12****, ASCC3, BABAM2, BANP,* ***CAMK2G****, CENPH, EYA2, HMG20B, MCM3,* ***PTPN14****,* ***RBL2****, RRS1, USP15* |
| CCL20 | cytokine | 6.30 × 10^-3^ | ***ACOT12****,* ***AFF3****, ASCC3, AURKA, BABAM2, BANP,* ***CAMK2G****, CENPH, EYA2,* ***GAB3****, HMG20B, POLE3, PRKCB, USP15* |
| APBB3 | other | 6.50 × 10^-3^ | ***AFF3****, AURKA,* ***CAMK2G****, EYA2, HMG20B, PRKCB,* ***RBL2****, RRS1, USP15* |
| manumycin A | chemical reagent | 6.70 × 10^-3^ | ***ACOT12****,* ***AFF3****, ASCC3, AURKA, BABAM2, BANP,* ***CAMK2G****, CENPH, EYA2,* ***GAB3****, HMG20B,* ***RBL2****, RRS1, VPS72* |
| sodium selenite | chemical drug | 6.70 × 10^-3^ | ***ACOT12****, ASCC3, AURKA, BANP,* ***CAMK2G****, CENPH, EYA2, HMG20B, MCM3, PRKCB,* ***RBL2****, RNF20, RRS1, USP15* |
| PTPN18 | phosphatase | 6.70 × 10^-3^ | ***ACOT12****,* ***AFF3****, AURKA, BANP, EYA2,* ***GAB3****, HMG20B, MCM3, PRKCB,* ***RBL2****, RNF20, RRS1, USP15* |
| saikosaponin A | chemical reagent | 6.70 × 10^-3^ | ***ACOT12****, AURKA, BABAM2,* ***CAMK2G****, CENPH,* ***GAB3****, HMG20B, RRS1, USP15* |
| GDF15 | growth factor | 6.80 × 10^-3^ | ***AFF3****, AURKA, BABAM2,* ***CAMK2G****,* ***GAB3****, HMG20B, MCM3, POLE3, PRKCB,* ***RBL2****, USP15, VPS72* |
| tyrphostin AG825 | chemical - kinase inhibitor | 6.80 × 10^-3^ | ***ACOT12****, AURKA, BABAM2, BANP,* ***CAMK2G****, CENPH, EYA2, HMG20B, MCM3, PRKCB,* ***RBL2****, RRS1, USP15* |
| MXD1 | transcription regulator | 6.90 × 10^-3^ | ***RBL2*** |
| Pkg | group | 7.00 × 10^-3^ | ***ACOT12****, ASCC3, AURKA, BANP,* ***CAMK2G****, CENPH,* ***GAB3****, MCM3, PRKCB,* ***PTPN14****, RNF20, RRS1, VPS72* |
| 24R, 25-dihydroxyvitamin D3 | chemical - endogenous mammalian | 7.10 × 10^-3^ | ***ACOT12****,* ***AFF3****, AURKA, BABAM2, BANP,* ***CAMK2G****, CENPH, EYA2,* ***GAB3****, HMG20B,* ***PTPN14****, USP15, VPS72* |
| ERRFI1 | other | 7.20 × 10^-3^ | ***ACOT12****, ASCC3, AURKA, BANP,* ***CAMK2G****, CENPH, EYA2,* ***GAB3****, HMG20B, MCM3, POLE3,* ***RBL2****, RRS1, USP15* |
| EPHB2 | kinase | 7.20 × 10^-3^ | ***AFF3****, ASCC3, AURKA, BABAM2, BANP,* ***CAMK2G****, CENPH,* ***GAB3****, HMG20B, POLE3, RRS1* |
| DUSP2 | phosphatase | 7.20 × 10^-3^ | ***AFF3****, AURKA, BABAM2, BANP,* ***CAMK2G****, EYA2,* ***GAB3****, HMG20B, USP15* |
| PCM1 | other | 7.20 × 10^-3^ | *MCM3* |
| GIPC1 | other | 7.30 × 10^-3^ | ***ACOT12****,* ***AFF3****, AURKA, BABAM2, BANP,* ***CAMK2G****, CENPH, EYA2,* ***GAB3****, HMG20B, PRKCB, RRS1, USP15* |
| ADRA2C | G-protein coupled receptor | 7.50 × 10^-3^ | ***ACOT12****,* ***AFF3****, AURKA, BABAM2, BANP,* ***CAMK2G****, CENPH, EYA2,* ***GAB3****, HMG20B, PRKCB, RRS1, USP15* |
| prosaptide d1 | biologic drug | 7.50 × 10^-3^ | ***ACOT12****,* ***AFF3****, AURKA, BABAM2, BANP,* ***CAMK2G****, CENPH, EYA2,* ***GAB3****, HMG20B, PRKCB, RRS1, USP15* |
| PTPRC | phosphatase | 7.60 × 10^-3^ | *ASCC3, AURKA, BABAM2, EYA2,* ***GAB3****, MCM3, POLE3,* ***PTPN14****,* ***RBL2****, RRS1, USP15, VPS72* |
| EPGN | growth factor | 7.60 × 10^-3^ | ***ACOT12****,* ***AFF3****, AURKA, BABAM2, BANP,* ***CAMK2G****, CENPH, EYA2,* ***GAB3****, HMG20B, POLE3, PRKCB, RRS1* |
| AURKAIP1 | enzyme | 7.60 × 10^-3^ | *AURKA* |
| tozasertib | chemical drug | 7.60 × 10^-3^ | *AURKA* |
| phytohemagglutinin | chemical drug | 7.70 × 10^-3^ | *ASCC3, BABAM2, BANP,* ***CAMK2G****, CENPH, EYA2, HMG20B, MCM3, PRKCB,* ***PTPN14****,* ***RBL2****, RRS1, USP15, VPS72* |
| dactolisib | chemical drug | 7.70 × 10^-3^ | ***AFF3****, ASCC3, AURKA, BABAM2, BANP, CENPH, MCM3,* ***PTPN14****,* ***RBL2****, RRS1, VPS72* |
| KLRC2 | transmembrane receptor | 7.70 × 10^-3^ | ***ACOT12****,* ***AFF3****, AURKA, BANP,* ***CAMK2G****, CENPH, EYA2,* ***GAB3****, HMG20B, PRKCB, RNF20, RRS1, USP15* |
| PPP2R2C | phosphatase | 7.80 × 10^-3^ | ***AFF3****, AURKA, BABAM2,* ***CAMK2G****, CENPH,* ***GAB3****, HMG20B,* ***PTPN14****,* ***RBL2****, RRS1* |
| cicletanine | chemical drug | 7.80 × 10^-3^ | *PRKCB* |
| nardostachys chinensis extract | chemical reagent | 7.80 × 10^-3^ | *PRKCB* |
| 3, 4-dideoxyglucoson × 10^-^3-ene | chemical - endogenous mammalian | 7.80 × 10^-3^ | *PRKCB* |
| BMS-214662 | chemical drug | 7.80 × 10^-3^ | *PRKCB* |
| enzastaurin | chemical drug | 7.80 × 10^-3^ | *PRKCB* |
| ginsenoside Rh2 | chemical - endogenous non-mammalian | 7.90 × 10^-3^ | ***AFF3****, BABAM2, BANP, EYA2, HMG20B,* ***PTPN14****,* ***RBL2****, RNF20, RRS1, USP15* |
| PD158780 | chemical - kinase inhibitor | 8.10 × 10^-3^ | ***ACOT12****, ASCC3, AURKA, BABAM2, BANP,* ***CAMK2G****, CENPH, EYA2,* ***GAB3****, HMG20B, POLE3, RRS1, USP15, VPS72* |
| TRAF2 | enzyme | 8.20 × 10^-3^ | *AURKA,* ***PTPN14*** |
| DUSP22 | phosphatase | 8.40 × 10^-3^ | ***ACOT12****, AURKA, BABAM2, BANP,* ***CAMK2G****, EYA2,* ***GAB3****, HMG20B, RNF20* |
| MET | kinase | 8.50 × 10^-3^ | ***ACOT12****, ASCC3, AURKA,* ***CAMK2G****, EYA2,* ***GAB3****, HMG20B, POLE3,* ***PTPN14****,* ***RBL2****, RNF20, RRS1, VPS72* |
| des-Arg(10)-kallidin | chemical reagent | 8.60 × 10^-3^ | ***ACOT12****,* ***AFF3****, BANP,* ***CAMK2G****, CENPH, EYA2, HMG20B, PRKCB,* ***PTPN14****,* ***RBL2****, USP15* |
| SULF1 | enzyme | 8.70 × 10^-3^ | ***ACOT12****,* ***AFF3****, AURKA, BABAM2, BANP,* ***CAMK2G****, CENPH, EYA2,* ***GAB3****, HMG20B, RRS1, USP15* |
| NTF4 | growth factor | 8.80 × 10^-3^ | ***ACOT12****,* ***AFF3****, AURKA, BABAM2, BANP,* ***CAMK2G****, CENPH, EYA2,* ***GAB3****, HMG20B, PRKCB, RRS1, USP15* |
| CST5 | other | 8.80 × 10^-3^ | ***CAMK2G****, RRS1, STN1* |
| AGA | enzyme | 8.80 × 10^-3^ | *MCM3* |
| Eph Receptor | group | 8.90 × 10^-3^ | ***AFF3****, AURKA, BABAM2, BANP,* ***CAMK2G****, CENPH,* ***GAB3****, POLE3, USP15* |
| L-685, 458 | chemical - protease inhibitor | 9.20 × 10^-3^ | ***ACOT12****,* ***AFF3****, AURKA, BANP,* ***CAMK2G****, CENPH, EYA2, HMG20B, MCM3, PRKCB,* ***RBL2****, RRS1, USP15* |
| ADRA2B | G-protein coupled receptor | 9.20 × 10^-3^ | ***ACOT12****,* ***AFF3****, AURKA, BABAM2, BANP,* ***CAMK2G****, CENPH, EYA2,* ***GAB3****, HMG20B, PRKCB, RRS1, USP15* |
| quetiapine | chemical drug | 9.20 × 10^-3^ | ***ACOT12****,* ***AFF3****, AURKA, BANP,* ***CAMK2G****, CENPH, EYA2,* ***GAB3****, HMG20B, PRKCB, USP15* |
| phosphorothioate oligodeoxynucleotide | chemical - other | 9.30 × 10^-3^ | ***ACOT12****,* ***AFF3****, AURKA, BABAM2, BANP,* ***CAMK2G****, CENPH, EYA2,* ***GAB3****, HMG20B, PRKCB,* ***PTPN14****, USP15* |
| cobimetinib | chemical drug | 9.50 × 10^-3^ | ***ACOT12****,* ***AFF3****, BABAM2, BANP,* ***CAMK2G****, CENPH, EYA2, HMG20B, PRKCB, RRS1, USP15, VPS72* |
| cediranib | chemical drug | 9.60 × 10^-3^ | ***ACOT12****,* ***AFF3****, BANP,* ***CAMK2G****, CENPH, EYA2, HMG20B, PRKCB,* ***RBL2****, RRS1, USP15, VPS72* |
| BAP1 | peptidase | 9.60 × 10^-3^ | *MCM3* |
| quinacrine | chemical drug | 9.80 × 10^-3^ | ***ACOT12****,* ***AFF3****, AURKA, BABAM2, BANP,* ***CAMK2G****, CENPH,* ***GAB3****, HMG20B, MCM3,* ***PTPN14****,* ***RBL2****, USP15, VPS72* |
| GRK3 | kinase | 9.80 × 10^-3^ | ***ACOT12****,* ***AFF3****, BABAM2, BANP,* ***CAMK2G****, CENPH, EYA2, HMG20B, PRKCB, VPS72* |
| parathyroid hormon × 10^-^related protein | biologic drug | 9.80 × 10^-3^ | ***ACOT12****,* ***AFF3****, BANP,* ***CAMK2G****, CENPH, EYA2, HMG20B, PRKCB,* ***RBL2****, RRS1, USP15, VPS72* |
| pegdinetanib | biologic drug | 9.90 × 10^-3^ | ***ACOT12****,* ***AFF3****, BANP,* ***CAMK2G****, CENPH, EYA2, HMG20B, PRKCB,* ***RBL2****, RRS1, USP15, VPS72* |

^1^Master regulators are molecules that indirectly control multiple genes in a pathway within the Ingenuity Pathway Analysis.

^2^Molecule type of the master regulator as defined by the Ingenuity Pathway Analysis.

^3^Network bias corrected *P* - value calculated by Ingenuity Pathway Analysis.

^4^ List of the positional candidate genes from the genome-wide association analysis (in **bold**) and leading-edge genes from the gene-set enrichment analysis-SNP regulated by the upstream regulator.
